# Tailoring the molecular structure of crosslinked polymers for pervaporation desalination

**DOI:** 10.1038/s41467-020-15038-w

**Published:** 2020-03-19

**Authors:** Yun Long Xue, Jin Huang, Cher Hon Lau, Bing Cao, Pei Li

**Affiliations:** 1 College of Materials Science and Engineering, Beijing University of Chemical Technology, 100029 Beijing, China; 2 College of Engineering & Computer Science, Wright State University, 3640 Colonel Glenn Highway, Dayton, OH 45435 USA; 3 School of Engineering, The University of Edinburgh, Robert Stevenson Road, The King’s Buildings, Edinburgh, EH9 3FB Scotland, UK

**Keywords:** Polymers, Polymers, Computational science

## Abstract

Polymer crosslinking imbues chemical stability to thin films at the expense of lower molecular transportation rates. Here in this work we deployed molecular dynamics simulations to optimise the selection of crosslinking compounds that overcome this trade-off relationship. We validated these simulations using a series of experiments and exploited this finding to underpin the development of a pervaporation (PV) desalination thin-film composite membrane with water fluxes reaching 234.9 ± 8.1 kg m^−2^ h^−1^ and salt rejection of 99.7 ± 0.2 %, outperforming existing membranes for pervaporation and membrane distillation. Key to achieving this state-of-the-art desalination performance is the spray coating of 0.73 μm thick crosslinked dense, hydrophilic polymers on to electrospun nanofiber mats. The desalination performances of our polymer nanocomposites are harnessed here in this work to produce freshwater from brackish water, seawater and brine solutions, addressing the key environmental issue of freshwater scarcity.

## Abstract (web summary)

Polymer crosslinking in desalination membranes adds stability on the cost of molecular transportation rates through the membrane. Here the authors tailor crosslinking of desalination membranes to overcome the stability and transport trade-off, and demonstrate a pervaporation desalination thin-film composite membrane with high water flux.

## Introduction

Desalination is a mature technology for addressing freshwater scarcity by removing salt (sodium chloride (NaCl)) from seawater. Common polymer membrane technologies deployed in desalination include seawater reverse osmosis (SWRO)^1,2^, membrane distillation (MD)^3^, and pervaporation (PV)^4–7^. SWRO is the preferred technology due to low energy cost for producing one cubic meter of clean water (2–4 kw h m^−3^) when compared to thermally driven processes such as MD, multi-stage flashing, or multi-effect distillation where energy costs are twice as for SWRO (3–5.5 kw h m^−3^) for creating a phase change in water to drive separations^2,8^. However, SWRO can only process seawater with NaCl content between 3 and 4 wt.% and generates large amounts of brine solution (NaCl content >5 wt.%) that are difficult to treat^9^. Meanwhile, the use of hydrophobic carbonaceous membranes with water flux reaching 179 kg m^−2^ h^−1^ in MD can inhibit brine generation^3^, but such membranes are susceptible to fouling, affecting separation performance stability^6,10–12^. Alternatively, PV, a thermally driven process with energy costs that are identical to the higher end of SWRO energy costs, can utilize low grade (waste heat from industrial processes) or renewable (from solar or geothermal energy) heat to drive water separations from seawater at 40–75 °C, while the use of hydrophilic polymers in PV can overcome the detriments of fouling plaguing MD membranes^5^. Such membranes typically exist as thin-film composites (TFCs) where hydrophilic polymers are deposited as dense selective layers on to porous supports. However, the dense selective layer is also the Achilles heel of PV membranes for desalination as the water flux of such membranes is typically 2–3-fold lower than MD^13^.

To address this limitation of PV membranes, we developed a three-prong strategy to tailor the material and chemical properties of a hydrophilic polymer, polyvinyl alcohol (PVA), to produce an ultrapermeable TFC nanostructure. This strategy included (1) optimization of a sulfonic acid-loaded crosslinking compound via molecular dynamics simulation with experimental verification, (2) stability and water affinity enhancement of PVA to transform ubiquitous materials into porous supports that provide minimal transport resistance for the targeted molecules, and (3) scalable fabrication of thin PVA layers that can withstand the rigors of PV. During PV desalination, water concentration across most parts of the dense layer is close to sorption equilibrium at the feed side but not at the bottom edge. Therefore, to increase water permeation, we incorporated functional groups that enable facilitated water transport via polymer crosslinking, especially at the “dry” region^14^. This strategy was based on a hypothesis underpinned by the incorporation of additional water diffusion routes via adjacent carrier–water complexes^14,15^.

Here, in this work, we validated this hypothesis by fabricating TFC membranes comprising crosslinked, dense PVA selective layers using four crosslinkers (sulfosuccinic acid (SSA), an aliphatic molecule; 4-sulfophthanlic acid (SPTA), an aromatic molecule; poly(acrylic acid-co-2-acrylamido-2-methyl propane sulfonic acid) (P(AA-AMPS)), an aliphatic polymer; and poly(acrylic acid-co-sulfonated styrene (P(AASS)), an aliphatic polymer with aromatic side chains) and three types of porous substrates (chlorinated polyvinyl chloride (CPVC) ultrafiltration membrane, alumina disc, and electrospun polyacrylonitrile (PAN) nanofiber mats). Molecular dynamic simulations were deployed here to elucidate the effects of crosslinking via chemical compounds that contain water–carrier complexes on the excellent PV desalination performances and mechanical properties of crosslinked PVA selective layers produced here. Among all the PVA films studied here, P(AA-AMPS) crosslinked PVA showed the highest mechanical strength. The deposition of this composite as a selective layer on nanofibrous PAN supports via spray coating yielded TFC membranes with desalination performances that outperformed current PV desalination^6,16^ (by a factor of 3–20-fold), state-of-the-art vacuum^3^, and direct contact MD membranes^17^.

## Results

### Optimizing crosslinking in selective layer

**Fig. 1:**
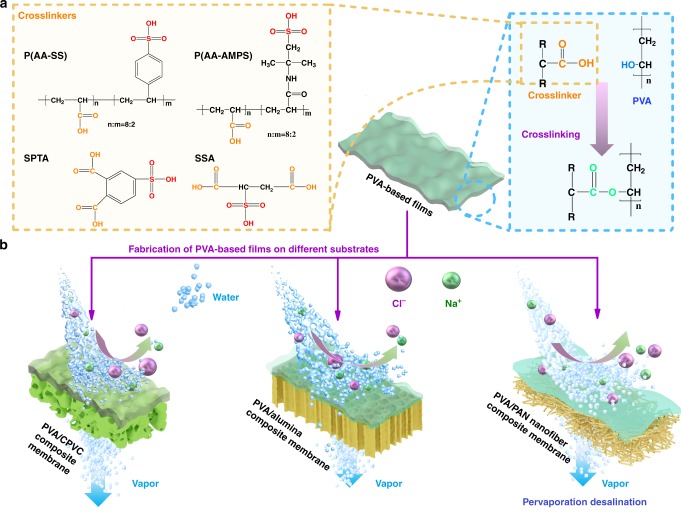
Structures of the crosslinkers and composite PV membranes. **a** The molecular structures of crosslinkers and the reaction scheme for crosslinking PVA films. **b** The diagrams of water transport from a NaCl feed solution through the PVA-based TFC membranes using porous substrates, including CPVC ultrafiltration membrane, alumina disc, and PAN nanofiber mats.

**Fig. 2:**
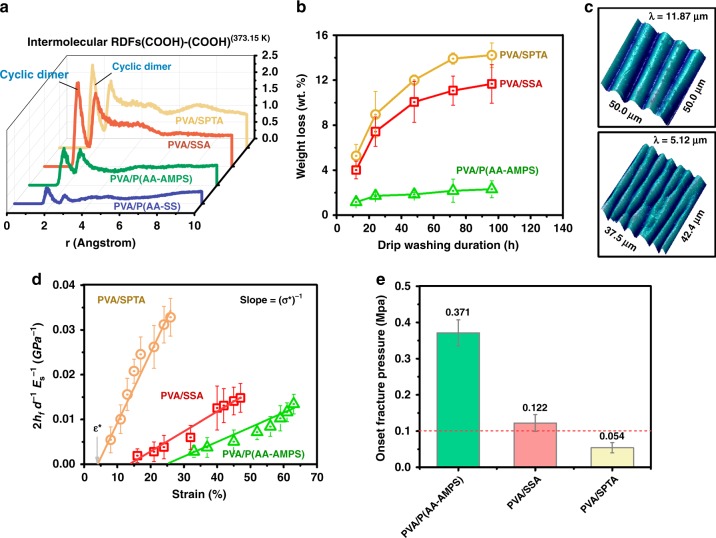
Characterization of the crosslinked PVA films. **a** The radial distribution functions (RDFs) of the COOH/COOH groups in the PVA/crosslinkers blending cells at 373.15 K. **b** The weight losses of the free-standing crosslinked PVA thick films during the drip-washing test at 80 °C for 4 days. **c** The wrinkle patterns of the crosslinked PVA thin films observed using AFM. **d** The crack density ($2h_{\rm{f}}\;d^{ - 1}E_{\rm{s}}^{ - 1}$) plotted against the applied strain (*ε*, %) for the water-swollen crosslinked PVA thin films. **e** The onset fracture pressures of the PVA/PAN nanofiber composite membranes. (lines with double caps are error bars, mean ± s.d. for *n* = 3).

The choice of crosslinking compound is crucial for determining the suitability of dense PVA films for PV desalination. Here we deployed molecular dynamics simulations to reveal the interactions between four different types of crosslinking compounds comprising sulfonic acid groups with PVA chains. These compounds included SSA, SPTA, P(AA-AMPS), and P(AASS) (the crosslinkers’ structures can be found in Fig. 1a and Supplementary Table 1). The sulfonic acid groups of crosslinkers were key to increasing water flux despite a higher crosslinking degree^14^. Through these molecular dynamics simulations, we were also able to reveal the thermodynamic compatibilities between the crosslinkers, i.e., the solubility parameter (*δ*, Supplementary Fig. 3) and heat of mixing (*ΔH*, Supplementary Fig. 5), with the PVA matrix. Monomeric crosslinkers SSA and SPTA were not as compatible to PVA, i.e., high *ΔH* and large *δ* values, when compared to the polymeric compounds. The monomeric crosslinkers, however, preferred to interact with each other, forming carboxylic dimers with strong hydrogen bonds that were difficult to break apart by the relatively weaker interactions between the PVA hydroxyl groups and the carboxylic acid groups from the crosslinking compounds, even at the crosslinking temperature of 100 °C (Fig. 2a and Supplementary Fig. 6). Other than affecting their miscibility with PVA, it was also difficult to drive the crosslinking with PVA chains via esterification, creating essentially blended nanocomposites rather than crosslinked polymers. Longer chains and asymmetrical structures minimized the formation of carboxylic dimers between polymeric crosslinkers (P(AA-AMPS) and P(AASS)), enabling esterification reactions that underpin crosslinking with PVA chains.

We validated these simulations through drip-washing and water immersion experiments performed on 48 ± 5 μm free-standing PVA films crosslinked with SSA, SPTA, and P(AA-AMPS) (Supplementary Table 4). The possible crosslinking and grafting mechanisms with proposed molecular structures of the crosslinked PVA films are shown in Supplementary Fig. 7. PVA films crosslinked with monomeric compounds—SSA and SPTA–demonstrated poor hydrostability in drip-washing tests as weight loss was 5–6-fold higher than the 2.3 ± 0.7 wt.% loss in P(AA-AMPS)-crosslinked PVA films (Fig. 2b and Supplementary Fig. 10a). Moreover, the C=O groups from Fourier transform infrared spectroscopy (FTIR) spectra of SSA- and SPTA-crosslinked PVA films was obviously decreasing during the drip-washing test (Supplementary Fig. 10c, d) that was reflective of their poor hydrostability and low crosslinking degree (Supplementary Table 4). We also determined the Young’s modulus (*E*_f_), fracture strength (σ*), and onset fracture strain (ε*) of crosslinked PVA films swollen with water via strain-induced wrinkling^18,19^ and cracking^18,20^ tests (Fig. 2c, d, Supplementary Figs. 13–15). The mechanical properties of P(AA-AMPS)-crosslinked PVA films were far more superior than those of SSA- and SPTA-crosslinked films (Supplementary Table 6). The film with the best mechanical properties—*E*_f_, σ*, and ε*—enabled a TFC membrane the highest fracture pressure during operation, i.e., rupturing of the selective layer at the highest possible transmembrane pressure.

Using this finding, we deposited 1.7-μm-thick PVA films crosslinked by SSA, SPTA, and P(AA-AMPS) on to PAN nanofibrous mats (Supplementary Fig. 37b–d) and characterized these TFCs in an ultrafiltration cell (Supplementary Fig. 36). A gradual increase in transmembrane pressure led to the rupture of SSA-crosslinked PVA films at 0.122 ± 0.023 MPa, SPTA-crosslinked PVA films at 0.054 ± 0.014 MPa, and P(AA-AMPS)-crosslinked PVA films at 0.371 ± 0.036 MPa (Fig. 2e and Supplementary Fig. 37a). Only P(AA-AMPS)-crosslinked PVA films maintained structural integrity and survived a vacuum pull on the permeate side during PV test.

### Characterization of porous support layer

**Fig. 3:**
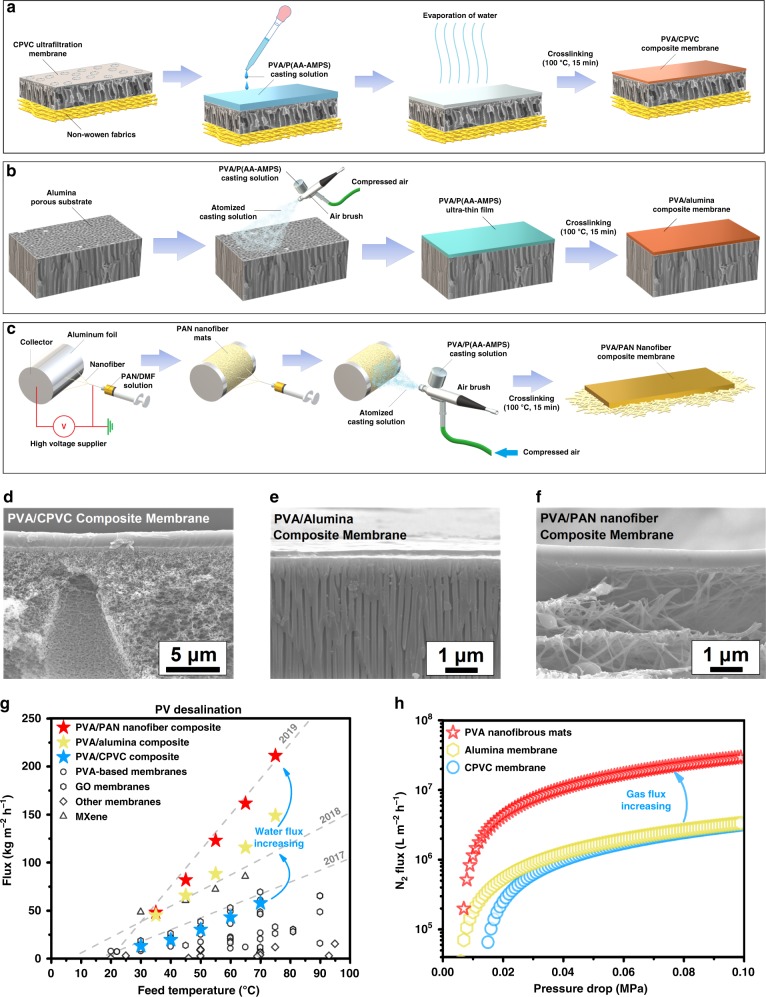
Preparation protocols and desalination performance of PV membranes. Preparation protocols and cross-sectional morphologies of composite membranes: **a**, **d** PVA/CPVC; **b**, **e** PVA/Alumina; **c**, **f** PVA/PAN nanofiber mats. **g** Comparison of desalination performances of our PV membranes with the reported data (Supplementary Table 9). **h** Relationships between N_2_ fluxes (L m^−2^ h^−1^) and pressure drops (MPa) of the porous substrates used in this work.

Pores play a critical role in reducing resistance within the support layer for the transportation of gaseous water^21,22^ while optimizing the deposition of the selective layer. To strike a balance between high fluxes, mechanical stability, and compatibility with the selective layer, we investigated the porosity and pore size of three different types of support layers—a conventional support in the form of a CPVC ultrafiltration membrane produced from nonsolvent-induced phase separation (NIPS; Fig. 3a and Supplementary Figs. 21 and 22), a commercially available alumina membrane (Anodisc 25^TM^, Whatman^TM^, Germany, Fig. 3b and Supplementary Fig. 29b, c) and an electrospun PAN nanofibrous mat (Fig. 3c and Supplementary Fig. 34). The alumina support possessed the smallest average diameter at 17 nm and the highest surface porosity at 47.1%, while the average pore size diameter of the nanofibrous support was the largest at 149 nm and the NIPS support had the lowest surface porosity of 7.2%.

### PV desalination performance

To determine the effect of P(AA-AMPS) crosslinker concentration on the desalination performances of films studied here, we deposited PVA films comprising 10–30 wt.% of P(AA-AMPS) crosslinkers on to NIPS support layers (Supplementary Fig. 25)—the easiest to fabricate among all three support types. The desalination performances of these films were determined using an aqueous solution comprising 3.5 wt.% NaCl as feed at 70 °C. The salt rejection of these films reached 99.9 ± 0.1%, while water fluxes increased from 48.6 ± 2.3 to 57.9 ± 1.8 kg m^−2^ h^−1^ as P(AA-AMPS) content increased from 10 to 30 wt.% (Supplementary Fig. 27). The increase in P(AA-AMPS) content also enhanced crosslinking densities, lowered hydrophilicities, and reduced water swelling^14,23^. According to the solution–diffusion mechanism, such a combination will reduce water flux. Contrarily, here in this work, we observed the exact opposite, as water flux increased by 19% with a 20% in P(AA-AMPS) content. This was ascribed to higher concentrations of sulfonic acid functional groups within the PVA matrix that enabled facilitated transport of water molecules^14^.

Based on this finding, we formulated a coating solution comprising PVA/P(AA-AMPS) (70/30 w/w) and spray coated this solution on to alumina (Fig. 3e and Supplementary Fig. 29a) and PAN nanofibrous support layers (Fig. 3f and Supplementary Fig. 35). By controlling the spraying duration from 4 to 8 s, we were able to produce PVA films with thicknesses between 72 ± 15 and 387 ± 32 nm, respectively, on alumina supports (Supplementary Fig. 31). Benefitting from the low transport resistance afforded by these ultrathin films, the water fluxes of these TFC membranes reached 97.1 ± 7.3 to 148.1 ± 7.7 kg m^−2^ h^−1^ with an NaCl rejection rate of >99.6 ± 0.4% when separating a 3.5 wt.% NaCl solution at 75 °C (Fig. 3g and Supplementary Fig. 32a). The water fluxes of these spray-coated P(AA-AMPS)-crosslinked PVA/alumina TFC membranes were 10–70% higher than state-of-the-art MXene/PAN composite PV desalination membranes (Supplementary Fig. 32b).

Attributing to the larger surface pores in electrospun nanofibrous mats, we were only able to produce defect-free P(AA-AMPS)-crosslinked films of 730–3210 nm (Supplementary Fig. 38), at least double the thickness of PVA films spray coated on alumina (387 nm). This was due to polymer intrusion into the larger surface pores of nanofibrous supports during deposition. However, larger surface pores were beneficial for enhancing the water fluxes of these nanocomposites reaching 211.4 ± 11.3 kg m^−2^ h^−1^ with NaCl rejection of 99.8 ± 0.2% (Fig. 3g and Supplementary Fig. 39b). The relative water flux increase in these PVA/nanofiber membranes was not as significant as those on alumina supports. This was due to the thicker selective layers. Compared to PVA films with identical thicknesses that were deposited on CPVC supports, the water flux of PVA/nanofiber composites were 240% higher (Fig. 3g).

Clearly, this significant increase in water flux was attributed to the lower vapor transport resistance of the PAN nanofibrous support. The support layer with the largest average pore diameter, i.e., the nanofibrous mats, possessed the highest N_2_ flux, while the close average pore sizes of both the CPVC (23 nm) and alumina (17 nm) attributed to similar N_2_ fluxes (Fig. 3h and Supplementary Fig. 41a; N_2_ fluxes tested by gas permeation cell, Supplementary Fig. 40). Assuming that these support layers were deployed in vacuum MD at 75 °C without pore wetting, the nanofibrous support would have a theoretical water flux of 6230 kg m^−2^ h^−1^, 21–28 times more than the water fluxes of the CPVC (226 kg m^−2^ h^−1^) and alumina supports (290 kg m^−2^ h^−1^), respectively. This was similar to pure water flux of nanofibrous mats produced for microfiltration^24^ and was well explained by Poiseuille’s Law where flux was directly proportional to the second power of the pore radius (Supplementary Eq. 16). This also underpinned the superior PV desalination performances of TFCs fabricated here in this work when compared to state-of-the-art PV membranes (Fig. 3g). The combination of the mechanically stable P(AA-AMPS)-crosslinked PVA coating layer and electrospun PAN nanofibrous support was ideal for producing a TFC structure that could withstand the rigors of PV desalination without sacrificing separation performances.

### Comparison of desalination performance between PV and MD

**Fig. 4:**
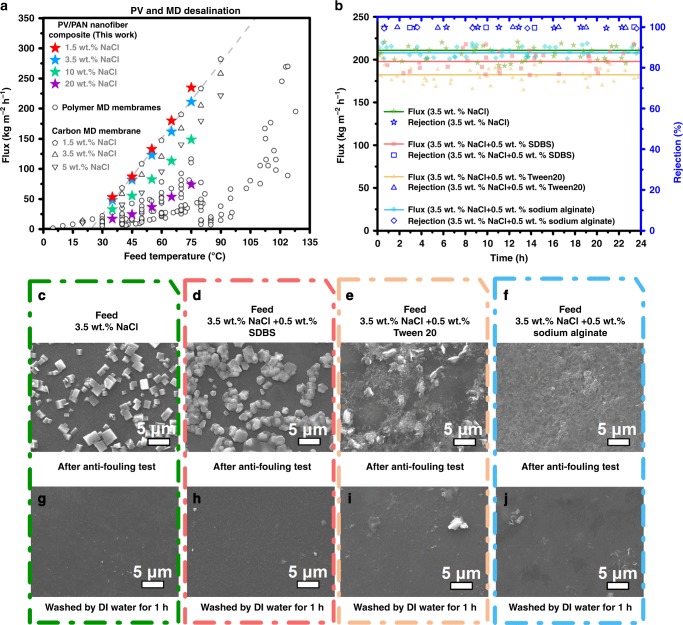
Desalination properties of brine and polluted salt solutions. **a** Comparison of the water fluxes of PVA/PAN nanofiber composite membranes (in this study) to the reported membrane distillation (MD) data (Supplementary Fig. 42b). **b** The desalination properties of the PVA/nanofiber composite membrane when treating a 3.5 wt.% NaCl solution and a 3.5 wt.% NaCl solution with 0.5 wt.% Tween 20 or sodium dodecyl benzene sulfonate (SDBS) or sodium alginate acting as an organic foulant in feed solution. **c**–**f** Surface images of PVA/P(AA-AMPS) coating layers after antifouling tests and **g**–**j** after washing by DI water for 1 h.

PV membranes are often compared with MD membranes for desalination^5^. This is because both processes are thermally driven and require identical equipment set-ups. Hence, we compared the desalination performances of our P(AA-AMPS)-crosslinked PVA/PAN nanofiber composite membranes with those of cutting-edge MD membranes (Fig. 4a). In the temperature range of 35–75 °C and across salt concentrations of 1.5–20 wt.%, our P(AA-AMPS)-crosslinked PVA/PAN nanofiber TFCs outperformed all materials deployed in MD. This was attributed to the fouling resistance of our material choice—a hydrophilic polymer in the form of PVA. MD membranes are prone to fouling from high salt concentrations and organic foulants as hydrophobic materials are required to prevent liquid entry into the pores^11,25^. For example, the pure water flux of a nanoporous carbonaceous material deployed in MD reach 400 kg m^−2^ h^−1^. However, the presence of 1.5 wt.% NaCl (similar to salt content in brackish water) reduced the water flux by 64%^3^. Meanwhile, the water flux of P(AA-AMPS)-PVA/PAN nanofiber membranes studied here was only reduced by 8%, reaching 234.9 ± 8.1 kg m^−2^ h^−1^ (Supplementary Fig. 42a).

### Long-term and anti-fouling performance of PV membrane

Using 3.5 wt.% NaCl solutions that contained 0.5 wt.% organic pollutants (commonly cleaning agents for protein^26,27^: Tween 20 (a non-ionic surfactant) or sodium dodecyl benzene sulfonate (SDBS, an anionic surfactant) or sodium alginate (main bio-foulant in membrane water treatment^28^), we determined the anti-fouling properties of P(AA-AMPS)-crosslinked PVA/PAN nanofiber TFCs studied here (Fig. 4b). Without these pollutants in the feed solution, we were able to collect 1.67 L of freshwater (salt rejection 99.8 ± 0.2%) using a PVA/PAN nanofiber membrane with an area of 3.28 × 10^-4^ m^2^ over 24 h using a 3.5 wt.% NaCl solution at 75 °C. Tween 20, SDBS, and sodium alginate reduced the amount of freshwater collected by 15%, 7% and 1%, respectively, with NaCl rejection >99.7 ± 0.2% over the 24-h test periods. The hydrophilicity of PVA underpinned its excellent anti-fouling properties that consequently prevented the build-up of organic foulants and salt scaling within the support layers. The small decrease in water flux could be attributed to the formation of a cake layer on the selective layer surface. However, this cake layer could be easily washed away with water, fully recovering lost water flux (Fig. 4c–j).

## Discussion

Here, in this work, we overcome the detrimental effect of polymer crosslinking on molecular transportation rates across a thin polymer film by optimizing the molecular structure and the amount of facilitated transport functional groups on crosslinking compounds via molecular dynamics simulations. We validated these simulation findings through a series of complimentary experiments where it was proven that PVA films crosslinked with aliphatic polymeric compounds containing sulfonic acid groups demonstrated the best hydrostability, mechanical properties, and desalination performances. We produced crosslinked PVA thin films by spray coating and tailored the structure of the porous layer via the traditional approach of NIPS and electrospinning to yield a TFC for PV desalination. The separation performance of this nanocomposite material outperformed state-of-the-art desalination membranes operated in PV or MD modes. The use of commercially available chemical compounds, polymers, and facile engineering protocols to produce crosslinked PVA TFC membranes makes it easy to scale up the production of these nanostructures for industrial applications in desalination, gas dehumidification, organic dehydration, or drug delivery.

## Methods

### Molecular simulation

Full-atom molecular models of chemicals used here—PVA, SPTA, SSA, P(AA-AMPS), and P(AASS)—were created by three-dimensional atomistic modeling methods^29^, and details of these structures can be found in Supplementary Table 1. All the orientation and initiator of polymer models studied here were set to be random, and the tacticity were atactic^30^. Molecular dynamics simulation was implemented with the Forcite Modules. PVA and crosslinker molecules were packed into a cubic simulation cell by amorphous cell calculation modules^30^, which generated polymer chains in a periodic box by sampling backbone torsion angles according to their Monte Carlo distribution^30^. This amorphous cell configuration was further equilibrated using a multistep protocol (Supplementary Fig. 1) to relax impracticable torsion angles, while PVA and crosslinkers were mixed intensively. Densities, solubility parameters (Supplementary Fig. 3), and heat of mixing (Supplementary Fig. 5) of the PVA/crosslinker systems at 0.1 MPa and 300 K were estimated based on the NPT (normal pressure and temperature) trajectory obtained from their amorphous cells; the simulation period was 5 ns. The intermolecular interactions (radial distribution functions) between -OH and -COOH at 300 and 373.15 K were evaluated using the NPT simulation at 0.1 MPa for 5 ns (Fig. 2a and Supplementary Fig. 6).

### Thick-film preparation

Six grams of PVA was dissolved in 94 mL of deionized (DI) water, stirred at 95 °C until a homogenous mixture was obtained. pH of the solution was adjusted to 1 by adding H_2_SO_4_. Then the crosslinker reagent was added to the solution. After degassing, the solution was cast into a copper ring on top of a polytetrafluoroethylene (PTFE) board. Thickness of the solution layer was maintained at 1 mm. Free-standing films (52 ± 5 μm) were obtained by evaporating the solution at 25 °C for 24 h. The films were crosslinked at 100 °C for 15 min. Using this method, the pure PVA and P(AA-AMPS) films were also prepared without thermal treatment. The thick films were used in tests including drip washing, swelling degree (SD), contact angle, tensile, FTIR, thermogravimetric analysis (TGA), and wide angle X-ray diffractometry (WAXD).

### Thin-film preparation

Films with thicknesses from 0.3 to 0.8 μm were prepared by spin-coating. The PTFE substrate was first sprayed with paint (MR. HOBBY, B-513 from GSI Creos Corporation) to prevent adhesion between the crosslinked PVA layer to the PTFE substrate. A solution of 2 wt.% PVA/crosslinker (pH = 1, H_2_SO_4_) was spin-coated on to the PTFE substrate. The paint prevented the adhesion of crosslinked PVA layers on the PTFE substrate. Briefly, the spin-coating process was achieved by dropping 2 mL of coating solution on top of a rotating PTFE substrate at 1500 rounds per minute (rpm) in 120 s. The rotation speed was increased to 2000 rpm for 300 s to dry the film.

Thin films (<100 nm) were prepared by spray coating and were used for wrinkling, cracking, atomic force microscopic (AFM), and transmission electron microscopic (TEM) tests. PVA solution was diluted to 0.4 wt.% and was sprayed on to the painted PTFE board using an airbrush. The nozzle diameter of airbrush was 0.15 mm, and the coating solution cup was 5 mL. The airbrush was operated in a translational oscillatory motion (speed: 6 cm s^−^^1^, oscillation distance: 3 cm) by a step motor and the distance between the airbrush to the painted PTFE board was fixed at 15 cm. The spraying time was 4 s to obtain a layer thickness <100 nm and the pressure of the compressed air was 0.05 bar.

The PVA/paint/PTFE structures were heated in a muffle furnace at 100 °C for 15 min to facilitate PVA crosslinking. The paint was removed from the substrate by soaking in *N*,*N*-dimethylformamide (DMF) at room temperature for 2 h. The PVA layer was removed from the substrate via immersion in anhydrous alcohol (25 °C). The film was dried before use.

The detailed preparation protocols for preparing thick films, sub-1 μm, and sub-100 nm free-standing crosslinked PVA films are shown in Supplementary Fig. 8.

### Characterization methods

Hydrostabilities of the crosslinked PVA films were determined by monitoring the weight loss (Fig. 2b) of the polymer films after soaking them in DI water bath at 80 °C to remove all soluble components using Supplementary Eq. 5. SD was calculated by Supplementary Eq. 6.

The estimation of molecular weight between the crosslink points (Mc) and the evaluation of chemical crosslinking density of crosslinked PVA-based films (mol m^−3^) can be calculated by Flory–Rehner relation, as shown in Supplementary Eqs. 7–10.

FTIR spectra of the crosslinked PVA-based films during (Supplementary Fig. 10b–d) and after (Supplementary Fig. 18a) the drip-washing tests were determined using a spectrophotometer (Nicolet IS5, Thermo Scientific, USA) in a scanning range from 400 to 4000 cm^−1^ in a reflection mode. Samples were vacuum dried before test. Every sample was scanned for 64 times, and the resolution of FTIR was 0.4 cm^−1^.

Thermal stabilities of polymer films (Supplementary Fig. 18b, c) were investigated using a TGA equipment (TA Q100, TA Instruments Inc., USA) in nitrogen atmosphere. Film samples were dried prior to the TGA characterization through a mild thermal treatment at 40 °C in vacuo. Then the dry samples were heated from an ambient temperature to 400 °C at a rate of 10 °C min^−1^ to monitor the weight loss.

WAXD of polymer films (Supplementary Fig. 18d) were carried out by a D8 Advance equipment (Bruker BioSpin, Germany) with a Cu Kα radiation wavelength of 1.54 Å. Dried PVA-based films were mounted on zero background plates and scanned over a 2*θ* range of 5–90° with a step size of 0.02° and a count time of 4 s per step.

Cross-sectional and surface morphologies of the wrinkled PVA-based films, porous substrates, and composite membranes were examined by a field-emission scanning electron microscopy (SEM, JSM-7800F, JEOL Ltd, Japan) at 15 kV. Samples were fractured in liquid nitrogen to get a smooth cross-sectional image. All samples were sputter coated by Pt (E-1030 ion Sputter, Hitachi Science Systems, Ltd., Japan) under vacuum. TEM for crosslinked PVA/P(AA-AMPS) films (Supplementary Fig. 19) was carried out using a HT7700 (HITACHI, Japan) operated at 100 kV with LaB_6_ cathodes.

Tensile tests of the free-standing PVA-based films (Supplementary Fig. 12) and polydimethylsiloxane (PDMS) substrate were conducted using a dynamic thermomechanical analysis equipment (DMA Q800, TA Instruments Inc., USA). Fully dried free-standing films and cured PDMS substrates were cut into rectangular shapes with a width of 2 ± 0.2 mm and a length of 25 ± 3 mm. Samples were fixed between two clamps for the tensile test. The sample length was 9.5 ± 0.3 mm. Temperature of the analysis chamber was controlled at 25 ± 1.2 °C. Test mode was set in controlled force at a ramping force 0.5 N min^−1^ to 17 N.

Dimension FastScan^TM^ (Bruker, CA, USA) AFM equipped with a SNL-10 type probe was used to measure the surface morphologies of the porous substrate and the PVA-based thin films used in the wrinkling tests. Samples were attached onto a vacuum fix disk. The images were captured under Tapping and ScanAsyst in air modes using a silicon tip on a nitride lever probe (Bruker, CA, USA) with a cantilever thickness of 600 nm. Cantilever resonance frequency was set in the range of 214–263 kHz with a nominal spring constant of 0.35 N m^−1^. A sampling resolution of at least 256 points per line with a scan rate of 0.5–0.6 Hz was used. The scanned sample size was set to be 50, 15, or 5 μm^2^. Bruker “NanoScope Analysis 1.80” data visualization and analysis software were used to analyze the AFM images.

### Mechanical properties of the swollen thin films

The wrinkling tests were carried out using the protocol described in the Supplementary Fig. 13c. Briefly, a fixed uniaxial tensile strain (*ε*, %) was applied to a PDMS substrate by a stretching tool (Supplementary Fig. 13b). A water-swollen PVA thin film was transferred on to the stretched PDMS substrate. Once the tensile stress was released, the thin PVA film buckled under the compressive stress transferred from the substrate. A well-defined wrinkle corresponding to the minimum energy configuration was observed^19^. The Young’s modulus (*E*_*f*_, MPa) of the thin film was calculated based on the wrinkling wavelength (*λ*, μm) using Supplementary Eq. 11^18,19^. Wrinkle patterns (Fig. 2c) were observed using an optical microscope (OM) and AFM. Thicknesses of thin films were measured by AFM (Supplementary Fig. 9) and SEM images (Supplementary Fig. 14).

The cracking tests were operated using the same stretching holder. The testing procedure was illustrated in the bottom images of Supplementary Fig. 13c. Specifically, a water-swollen thin film was placed on a PDMS substrate and slowly stretched. The film first buckled periodically at the lateral direction because of the shrinkage of the PDMS substrate and then cracked when the sample was further stretched. Details of the testing procedure to obtain the crack spacing (*d*, μm) as a function of the applied strain (*ε*, %) are available in Supplementary Fig. 15. Both the onset fracture strength (σ*, MPa) and the onset fracture strain (ε*, %) of the films were quantitatively analyzed by Supplementary Eq. 12^18,20^. The average crack spacing (*d*, μm) was determined from OM images. The relationship between the crack density ($2h_{\rm{f}}\;d^{ - 1}E_{\rm{s}}^{ - 1}$, MPa^−1^) and applied strain (*ε*, %) for the crosslinked PVA thin films was plotted in Fig. 2d and Supplementary Fig. 16. The slopes of the fitting lines ((σ*)^−1^, MPa^−1^) were used to calculate the onset fracture strength (σ*, MPa), and the intercepts to the *x*-axis referred to the onset fracture strains (ε*, %).

### Fabrication of the PVA/CPVC composite membranes

A CPVC dope consisting of CPVC/poly(styrene-co-maleic anhydride)/polyvinyl pyrrolidone/*N*, *N*-dimethylacetamide at a weight ratio of 16/4/4/76 was cast on a polyester fabric with a film thickness of 150 μm and then immersed in a DI water bath at 24 ± 1 °C to form the CPVC membrane (Supplementary Fig. 21). A series of PVA/CPVC composite membranes was prepared by coating 2.5 wt.% PVA casting solutions with different P(AA-AMPS) content on the top of CPVC porous substrate (Supplementary Fig. 24). The 2.5 wt.% PVA solution was diluted from 6 wt.% pristine PVA aqueous solution (pH = 1, H_2_SO_4_). Supplementary Table 7 lists the compositions of the PVA/P(AA-AMPS) solutions used for casting. The composite membrane was dried at 25 °C for 24 h and then heated at 100 °C in muffle furnace in air to crosslink the PVA layer. The membranes were soaked in room temperature water for 48 h to remove any soluble component and residual catalyst (H_2_SO_4_).

### Fabrication of the PVA/alumina composite membranes

The PVA coating layer on alumina membrane (Anodisc^TM^ 25, Whatman^TM^, Germany) were prepared by perpendicularly spray coating a 0.4 wt.% PVA/P(AA-AMPS) (7:3 w/w) solution (pH = 1, H_2_SO_4_) to the surface of the alumina membrane at a distance of 15 cm (Supplementary Fig. 29). The alumina membrane was affixed on to a PTFE board using scotch tape. The spraying times were controlled at 4 or 8 s. The airbrush was in a translational oscillatory motion (speed: 6 cm s^−1^, oscillation distance: 3 cm) perpendicular to the PTFE board. After spray coating, the composite membranes were heated at 100 °C for 15 min for crosslinking. At last, the membranes were soaked in water at room temperature for 48 h to remove any soluble component and residual acid (catalyst, H_2_SO_4_).

### Electrospinning of PAN nanofiber mats

Eight grams of PAN was dissolved in 92 g DMF and stirred at 50 °C for 24 h to obtain a homogeneous solution. The PAN/DMF solution was filled in a syringe equipped with a 0.7-mm spinneret. The applied electric voltage and the solution feed rate was 24 kV and 10 μL min^-1^. The spinneret had a translational oscillatory motion perpendicular to the collector rotation direction (oscillation distance was 30 cm) driven by a step motor. A rotating collector (diameter: 10 cm, width: 30 cm) covered by a glossy aluminum foil was used to collect the PAN nanofiber at a rotating speed of 180 rpm. The distance between the spinneret and the collector was 17 cm (Supplementary Fig. 33).

### Spray coating of PVA layer on PAN nanofiber mats

In all, 0.75 wt.% PVA coating solutions comprising 30 wt.% crosslinker reagents were sprayed on to PAN nanofibers in the form of a fine mist using the airbrush. The PAN nanofiber substrate was fixed on the collector rotated horizontally (rotation speed: 180 rpm). The airbrush was in a translational oscillatory motion (speed: 6 cm s^−1^) perpendicular to the collector rotation direction (oscillation distance was 30 cm) driven by a step motor (Supplementary Fig. 35). The total spray time for coating were varied from 10 to 40 s, to provide a uniform coating layer with different thickness. After evaporation of water, the PVA/PAN nanofiber composite membranes were crosslinked at 100 °C for 15 min. The membranes were then soaked in water at room temperature for 48 h to remove any soluble component and residual acid (catalyst, H_2_SO_4_) before further tests.

### PV desalination

Desalination performance of the PVA-based composite membranes were measured by a bespoke PV set-up^14^ (Supplementary Fig. 20). The effective membrane area was 3.28 cm^2^ and a feed solution comprising 3.5–20 wt.% NaCl was used. Pressure at the permeate side was maintained at 100 Pa. The permeate mass collected every 10 min for 5 times at least in a liquid nitrogen cold trap. Water flux (*J*: kg m^−2^ h^−1^) was determined using Supplementary Eq. 13. The NaCl rejections (*R*_NaCl_, %) were determined by Supplementary Eq. 14 in an indirect way. The long-term desalination and anti-fouling properties of the PVA/nanofiber composite membrane were evaluated by separating a 3.5 wt.% NaCl solution with 0.5 wt.% Tween 20 or SDBS or sodium alginate acting as an organic foulant in feed solution.

### Compression capacity of coating layers

Composite membranes were placed on top of a porous metal plate in a dead-end ultrafiltration device (HP4750, STERLITECH Corporation, USA, Supplementary Fig. 36). Water (5 cm in height) was placed on top of the composite membrane and upstream pressure was gradually increased by 0.005 MPa per 5 min until water passed to the permeate side. The onset spilled pressures were recorded to determine the compressive capacity of coating layers. The effective membrane area was 11.36 cm^2^, and the tests were operated at ambient temperature.

### Gas permeation tests for porous substrate

To determine the resistance of the porous substrates to water vapor permeation, a gas permeation cell (CAT.NO.XX4404700, MILLIPORE CORP., Japan) was used to correlate the relation between pressure build-up and gas flux, as shown in Supplementary Fig. 40. N_2_ was used as feed gas because it was easier to be operated than water vapor^22^. A detailed experimental procedure could be found in ref. ^31^. N_2_ volume flux (*Q*: L m^−2^ h^−1^) was calculated using Supplementary Eq. 15. The resistance could be estimated by calculating the slope of *Q* versus the transmembrane pressures.

## Supplementary information

Supplementary Information

## Data Availability

Data supporting the findings of this study are available within the article and the associated Supplementary Information. Any other data are available from the corresponding authors upon reasonable request.

## Code availability

The program code used for the molecular dynamics simulation is provided in Supplementary Information.
