# Supplementary material for: Tailoring the molecular structure of crosslinked polymers for pervaporation desalination
Source: Nat Commun. 2020 Mar 19;11:1461. doi: 10.1038/s41467-020-15038-w (PMC7081321; doi:10.1038/s41467-020-15038-w)
Supplement: Supplementary file 1 — Supplementary Information [file 41467_2020_15038_MOESM1_ESM.pdf]

Molecular dynamics (MD) simulation

A 3D-atomistic modeling method[1] was used to build the full-atom molecular models of polyvinyl alcohol (PVA) and the crosslinkers. Their molecular potential energies were calculated using the polymer consistent force field (PCFF)[2]. All molecular models (listed in **Supplementary Table 1)** were set to be random with atactic tacticities[3]. Note that, PVA typically had crystalline domains. We assumed 100% amorphous PVA to simplify the simulation process. Molecular dynamics simulation was carried out using the Forcite Modules. The cut-off distance of both the van der Waals (vdW) and electrostatic interactions was set at 12.5 Å[4]; the long-range vdW interactions were assessed by the tail correction; and the long-range electrostatic interactions were computed using the standard Ewald summation method[5]. Time integration was done using the standard velocity-Verlet algorithm[6] at a time interval of 1 fs. Energy minimization was implemented using a conjugate gradient method[7]. A Nose Hoover chain method was adopted to simulate the movements of polymer chains during heating and cooling processes (thermo and barostats)[8,9].

Amorphous cells construction and equilibration protocol

To estimate the mixing conditions of PVA with different crosslinkers, their molecules were packed into a cubic cell using the Amorphous Cell Calculation Modules. Polymer chains were generated in a periodic boundary box and the torsion angles of polymer backbones were sampled according to their Monte Carlo distributions[10]. Then, the configurations of the amorphous cell underwent an extensive multistep equilibration protocol to relax all impracticable torsion angles (as shown in **Supplementary Figure 1**). Details of the equilibration protocol were given below.

Step 1. Construct amorphous cells. The amorphous cells of pure components were built to calculate densities (ρ), and total solubility parameters (δ) of PVA and the 4 crosslinkers as listed in **Supplementary Table 1**. For polymers of PVA, poly acrylic acid co-2-acrylamido-2-methyl propane sulfonic acid P(AA-AMPS), or poly acrylic acid -co- sulfonated styrene P(AA-SS), their amorphous cells contained 5 molecules (N_{Chain}= 5) with a degree of polymerization (DP) varied from 30 to 300. While for small molecules including 4-sulfophthalic acid (SPTA) and Sulfosuccinic acid (SSA), their cells had 30 to 300 molecules ($N_{molecule}$=30-300). The calculated densities and solubility parameters of all molecules were independent to the DP and numbers of chains or molecules in each cell as indicated in **Supplementary Table 1**. The calculated densities and solubility parameters would be used to calculate heat of mixing of the PVA and crosslinker systems.

The PVA/crosslinker mixed cells were built using a similar method to construct the pure component cells. The PVA/SPTA mixed cell was built using the same composition of our previous work[11], where the cell had 3 PVA molecules with a DP of 180 and 43 SPTA crosslinkers. We adjusted the values of DP and number of molecules of other mixed cells to ensure a similar molar ratio of -OH (from PVA) to -COOH (from crosslinker) to that of the PVA/SPTA cell (6.27) as listed in **Supplementary Table 2**. This was to maintain a similar concentration of the -COOH crosslinking groups among different mixing systems. The initial cell density was set at 0.5 g cm^{-3} (This was to increase the chain mobility so that the two components could mixed easily). For every amorphous cell, 10 configurations were constructed.

Step 2. Mixing process. After a configuration was obtained, the position of all PVA atoms were fixed at their Cartesian positions. Then, an NVT simulation (0.1 ns, 600 K) was carried out to redistribute the crosslinker molecules using the COMPASS force field. Note that, the cell density was kept at 0.5 g cm^{-3} to facilitate the mixing of the crosslinker with PVA.

Step 3. Geometry Optimization. The mixed cell geometries were refined using the Geometry Optimization (Max iterations: 50000) to reduce the cell's potential energy. During this process, density gradually increased from 0.5 g cm^{-3} to about 0.9 g cm^{-3}. For every amorphous cell, the Geometry Optimization was carried out for all 10 configurations to find out the one with the lowest potential energy.

Step 4. Annealing process. Polymer chains at a configuration relative to the lowest energy obtained from the step 3 were relaxed via a simulative annealing process where the cell was periodically heated from 200 K to 600 K and then cooled down to 200 K. A NPT ensemble was adopted to calculate the energy change at different temperatures [3]. Then the cell geometry was optimized by Geometry Optimization (Max iterations: 40000). After repeated this process for 100 times (100 annealing cycles), The amorphous cells with the lowest energy states were obtained as shown in **Supplementary Figure 2**.

Step 5. Densities, solubility parameters and heat of mixing of the PVA/crosslinker systems. The Hansen solubility parameters were calculated using the following expression[12,13]:

$$\delta^2 = \delta_{vdW}^2 + \delta_E^2 + \delta_H^2 \quad (1)$$

where δ_{vdW}, δ_E , and δ_H were the van der Waals, electrostatic, and hydrogen bond components of the total δ, respectively. Densities (ρ) and solubility parameters (δ_{vdW}, δ_E, δ_H and δ) of PVA and crosslinkers at 0.1 MPa and 300 K were estimated based on the NPT trajectory obtained from their amorphous cells. The simulation period was 5 ns, and the values of solubility parameters were provided in **Supplementary Table 1** and **Supplementary Figure 3**.

Configurations of the PVA/crosslinker mixed cells were obtained via NPT simulation in a simulation period of 5 ns at 0.1 MPa and 300 K. Heat of mixing (ΔH, listed in **Supplementary Table 2** and **Supplementary Figure 5**) was estimated by assuming (1) zero entropy change (entropy change was trivial compared to enthalpy change) and (2) constant volume of the mixture. The thermodynamic compatibility between PVA and crosslinker could be indicated by ΔH, which was calculated using **Supplementary Equation 2**[3]:

$$\Delta H \approx \Delta E = \omega_{PVA} \frac{\delta_P^2}{\rho_{PVA}} + \omega_C \frac{\delta_C^2}{\rho_C} - E_{coh,P+C} \quad (2)$$

where

$$E_{coh,P+C} \equiv \frac{CED_{P+C}}{\rho_{P+C}} \quad (3)$$

$E_{coh,P+C}$ was the cohesive energy of cells that was the quotient of the cohesive energy density (CED_{P+C}) and cell density ρ_{P+C}. ω_{PVA} and ω_C were the mass fractions of PVA and crosslinkers; ρ_{PVA} was the density of PVA, and ρ_C was the density of crosslinker; δ_P and δ_C were the solubility parameters of PVA and crosslinker.

Step 6. Radial distribution functions (RDFs). The intermolecular interactions between -OH and -COOH (or-COOH and -COOH) at 300 K and 373.15 K (i.e. crosslinking temperature in this study) were

evaluated using the NPT simulation at 0.1 MPa for 5 ns. The results are given in **Supplementary Figure 6**.

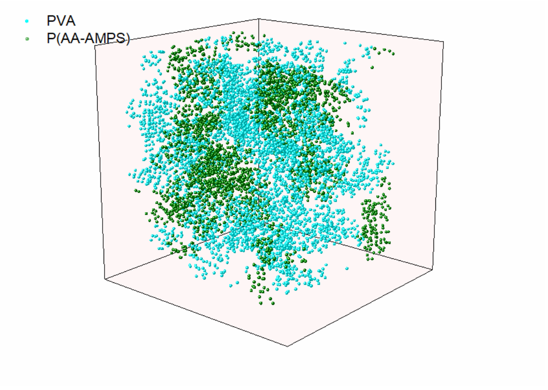

(a) After ***Step 1.*** (length = 51.38 Å)

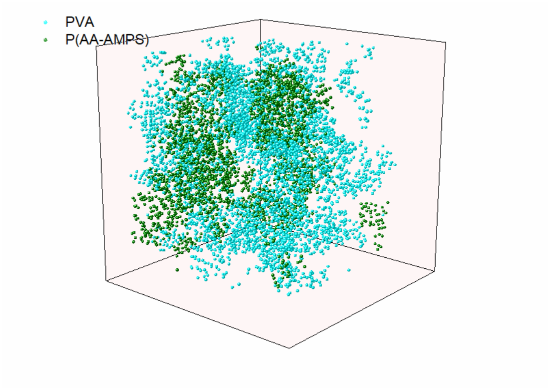

(b) After ***Step 2.*** (length = 51.38 Å)

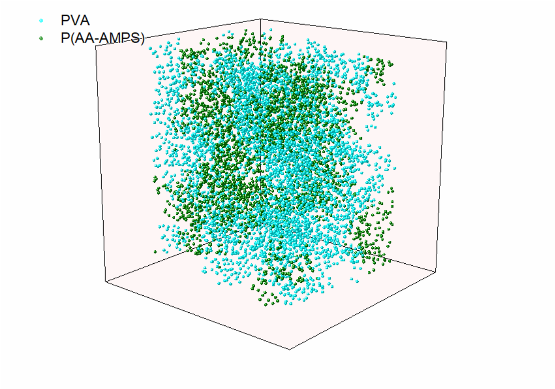

(c) After ***Step 3.*** (length = 42.87 Å)

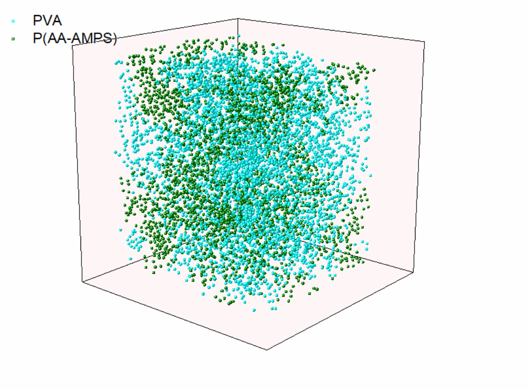

(d) After ***Step 4.*** (length = 39.78 Å)

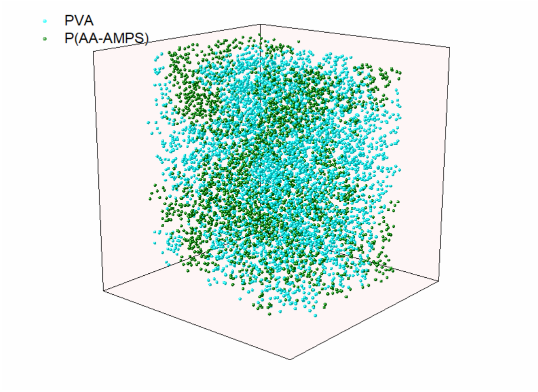

(e) After ***Step 5.*** (length = 38.13 Å)

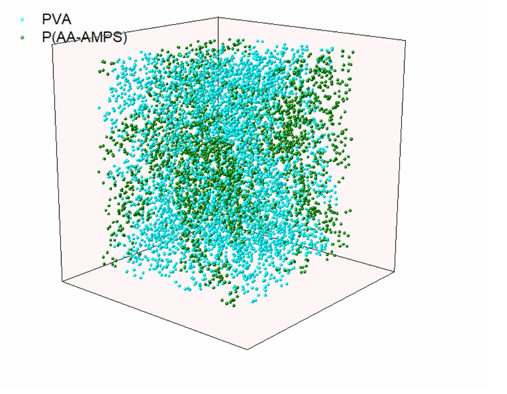

(f) After ***Step 6.*** (length = 38.77 Å)

Supplementary Figure 1: The equilibrium spatial distributions of the PVA and P(AA-AMPS) molecules in their blend cell after different simulation steps: (a) Step 1, (b) Step 2, (c) Step 3, (d) Step 4, (e) Step 5, (f) Step 6. The light blue dots represent the PVA atoms and the dark green dots represent the P(AA-AMPS) atoms.

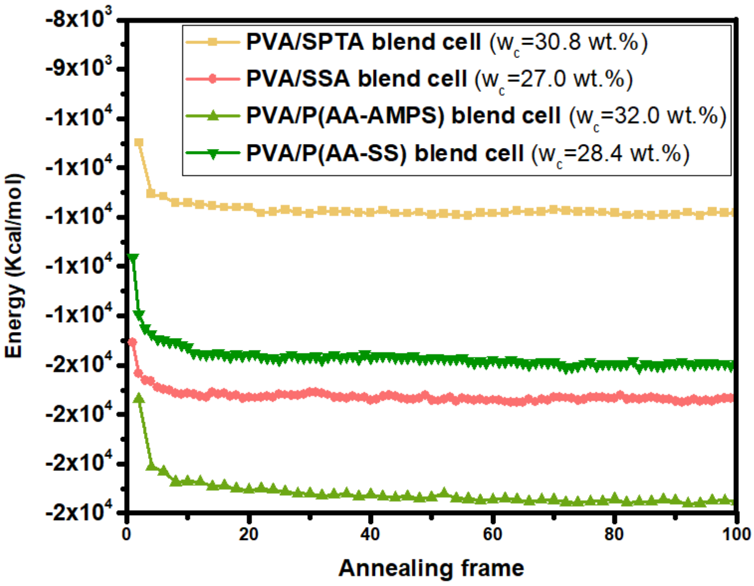

Supplementary Figure 2: Energy convergence of the PVA/crosslinker blend cells during the annealing process.

Solubility parameter (δ)

Supplementary Table 1. The calculated density (ρ), and solubility parameters (δ) of the pristine PVA and crosslinkers.

Full name and Molecule structure	N_{DP}	N_{Chain}	ρ (g cm^{-3}) (300 K)	δ (J cm^{-3})$^{0.5}$ (300 K)
Polyvinyl Alcohol (PVA) 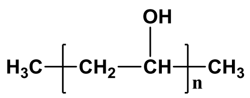	30	5	1.16±0.01	24.59±0.88
	60		1.17±0.01	25.71±0.79
	120		1.18±0.02	25.11±0.84
	180		1.17±0.02	24.44±0.49
	240		1.17±0.02	23.54±0.53
	300		1.17±0.02	24.50±0.39
Experimental data of PVA from Ref:	-	-	1.23-1.33 [a][14,15]	25.78 [a][14]<br>25-26 [a][14]
Simulation data of PVA from Ref:	50-400 [b][4]<br>145 [b][8]	1-8 [b][4]<br>1 [b][8]	1.15-1.31 [b][2,4,15]	28.5±3.1 [b][4]<br>22.5 [b][8]
Poly acrylic acid co-2-acrylamido-2-methyl propane sulfonic acid P(AA-AMPS) 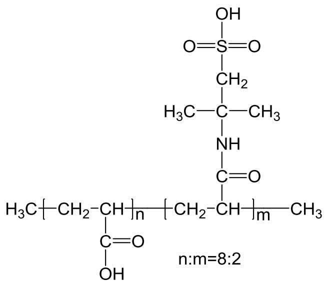	30	5	1.33±0.02	24.43±1.06
	60		1.30±0.03	22.85±1.77
	120		1.32±0.02	22.12±0.89
	180		1.29±0.02	23.76±0.62
	240		1.31±0.03	22.07±0.44
	300		1.34±0.03	23.68±0.72
Poly acrylic acid -co- sulfonated styrene P(AA-SS) 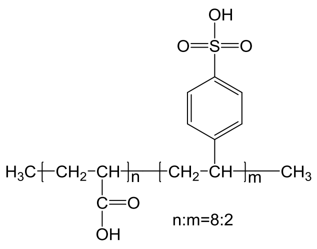	30	5	1.37±0.04	25.84±0.28
	60		1.40±0.01	23.53±0.16
	120		1.36±0.01	22.41±0.49
	180		1.35±0.05	23.75±0.74
	240		1.37±0.03	23.34±0.88
	300		1.36±0.02	24.12±0.39

To be continued

Continue

Full name and Molecule structure	N_{DP}	$N_{molecule}$	ρ (g cm^{-3}) (300 K)	δ (J cm^{-3})$^{0.5}$ (300 K)
4-sulfophthalic acid		30	1.62±0.02	36.34±0.63
(SPTA)		60	1.62±0.02	34.76±0.51
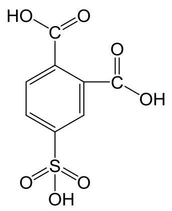		120	1.62±0.02	32.86±0.39
	-	180	1.62±0.03	36.23±0.48
		240	1.62±0.03	36.25±0.56
		300	1.63±0.02	36.37±0.53
Sulfosuccinic acid		30	1.77±0.02	35.57±0.46
(SSA)		60	1.74±0.01	34.93±0.21
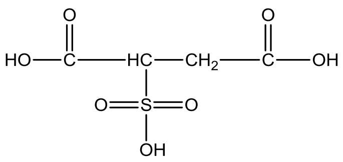		120	1.76±0.03	35.74±0.33
	-	180	1.78±0.02	36.33±0.17
		240	1.77±0.03	34.17±0.19
		300	1.75±0.02	35.43±0.25

[a] The experimental results at 298 K

[b] The MD simulation results at 300 K

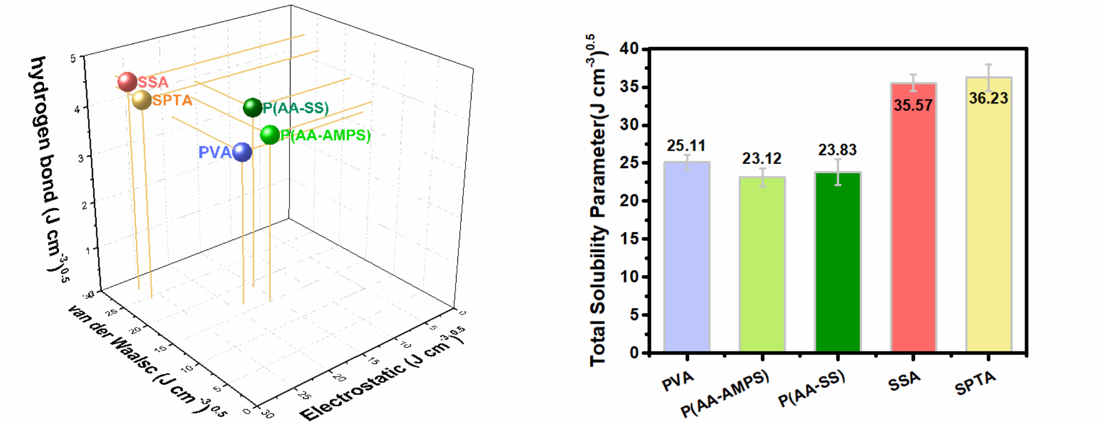

(a) (b)

Supplementary Figure 3: (a) The contribution of van der Waals (δ_{vdW}), electrostatic (δ_E), and hydrogen bond (δ_H) to the overall solubility parameter (δ) at 300 K. (b) The values of total solubility parameters obtained from the MD simulation at 300 K and 0.1MPa. (lines with double cap are error bars, mean ±s.d. for n = 6)

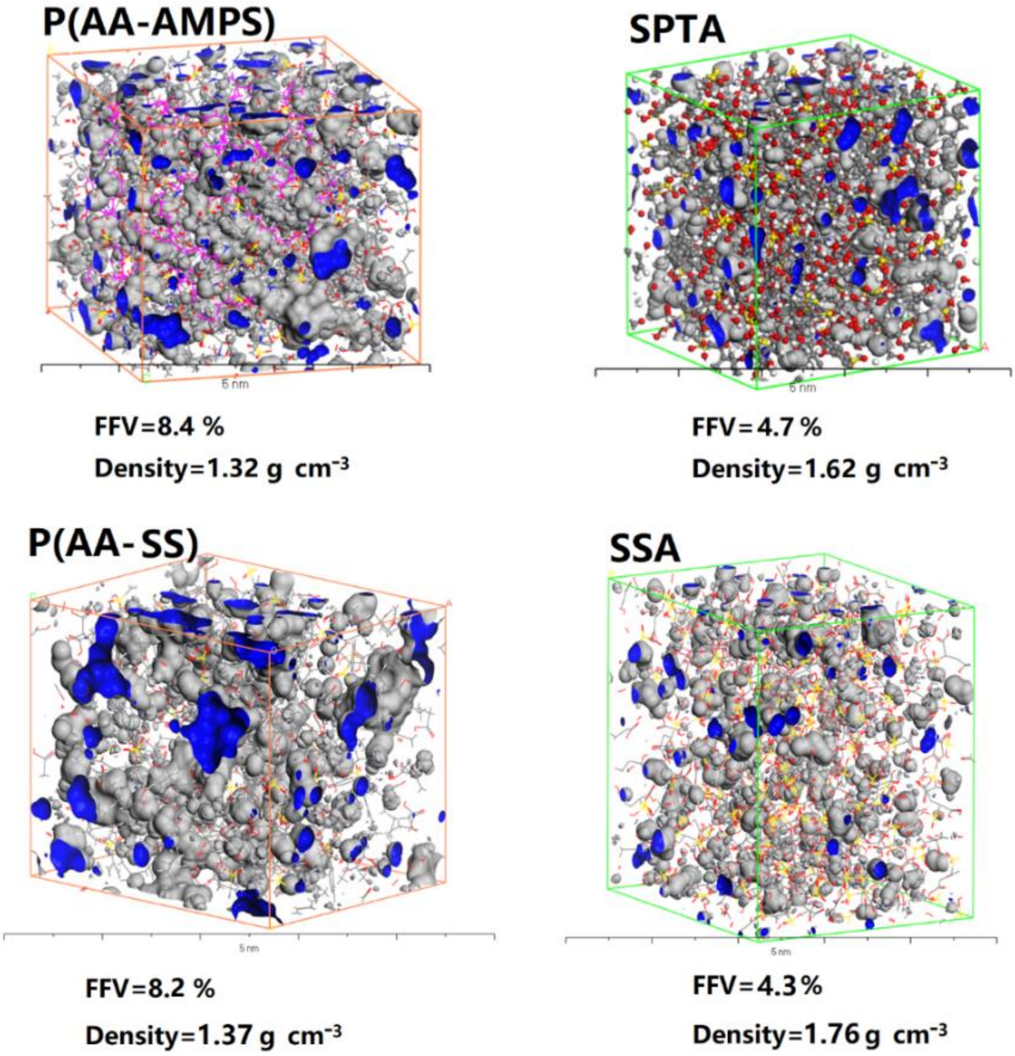

Supplementary Figure 4: The FFV and densities of the P(AA-AMPS), SPTA, P(AA-SS) and SSA at 300 K, 0.1MPa.

Supplementary note 1: The estimated value of δ_{PVA} was 25.11 ±1.01 (J cm^{-3})$^{0.5}$ that agreed well with the reported values of 25 to 28.5 (J cm^{-3})$^{0.5}$ in **Supplementary References** [4] and [14]. The predicted ρ_{PVA} were 1.16 to 1.18 g cm^{-3}, comparable to other simulation results of 1.15 to 1.31 g cm^{-3} [2,4,15], but were 10% lower than the experimental values of 1.22 to 1.33 g cm^{-3} [14,15]. This was due to that we assumed 100% amorphous polymer structure but PVA polymer typically had high-density crystalline regions in reality. The consistency between our results and the reported simulation data for PVA demonstrated the validity of the simulation protocols. **Supplementary Figure 3** showed that δ for high molecular weight crosslinkers, P(AA-AMPS) and P(AA-SS) were closer to PVA than the low molecular weight crosslinkers, SPTA and SSA. The lower δ values of the long-chain crosslinkers were due to that their large side groups (as drawn in **Supplementary Table 1**) enlarged polymer fractional free volume (FFV) (**Supplementary Figure 4**) and weakened the polar-polar interactions between -COOH from acrylic acid monomer (this would be further discussed by the results of RDFs, **Supplementary Figure 6**), whereupon reduced the cohesive energy (E_{coh}) and solubility parameter (δ).

Heat of mixing (ΔH)

Supplementary Table 2. Compositions, mass fraction of crosslinker (ω_C), mole ratio of (-OH):(-COOH), density of blend cells (ρ_{P+C}) and heat of mixing (ΔH) of the PVA/crosslinker blend cells.

Mixing conditions of PVA and crosslinkers		ω_C (wt. %)	Mole ratio of (-OH):(-COOH)	ρ_{P+C} (g cm^{-3}) (300 K)	ΔH (J g^{-1}) (300 K)
PVA+P(AA-AMPS)<br>N_{DP} of PVA = 120<br>N_{DP} of P(AA-AMPS) = 30<br>N_{Chain} of PVA = 5	N_{Chain} of P(AA-AMPS)<br>4	32.0	6.25	1.23±0.02	20.65±2.46
PVA+SPTA<br>N_{DP} of PVA = 180<br>N_{Chain} of PVA = 3	N_{SPTA} in cells<br>43	30.8	6.27	1.29±0.03	27.71±1.17
PVA+ P(AA-SS)<br>N_{DP} of PVA = 130<br>N_{DP} of P(AA-SS) = 30<br>N_{Chain} of PVA = 5	N_{Chain} of P(AA-SS)<br>4	28.4	6.45	1.23±0.03	13.86±1.94
PVA+SSA<br>N_{DP} of PVA = 180<br>N_{Chain} of PVA = 3	N_{SSA} in cells<br>45	27.0	6.13	1.29±0.02	27.82±2.47

Supplementary note 2: When a polymer mixture had strong polar interactions, such as the hydrogen bonding of the PVA/crosslinker system, miscibility was better described by the Gibbs energy of mixing (ΔG) than solubility parameter (δ) that was suitable for predicting non-polar systems [2]. However, the simulation process for calculating Gibbs energy of mixing for a polymer system would be very time-consuming owing to the large simulation time scale required for optimizing polymer configurations in the blending system. To simplify the mixing process, it was postulated that the conformational transform of the PVA/crosslinker systems was similar to the pure PVA system and the volume change during mixing was close to zero. Under this assumption, the entropy change would be trivial as compared to enthalpy change[3]. Hence, the heat of mixing (ΔH) was most indicative of the thermodynamic compatibility among different components in the blending system [3]. ΔH of all blending systems were calculated using **Supplementary Equations 2** and **3**. The results are given in **Supplementary Table 2** and **Supplementary Figure 5a**. In order to visually compared the simulation results, the atomic arrangement of the PVA/crosslinker blend cells obtained from ***Step 5*** were shown in **Supplementary Figures 5 b-e**. According to ΔH, the compatibilities of the crosslinkers to PVA decreased in an order of P(AA-SS) ＞ P(AA-AMPS) ＞ SPTA ＞ SSA (**Supplementary Figure 5a**). This trend agreed well with the solubility parameters of the 4 crosslinkers (**Supplementary Table 1)** where δ of P(AA-SS) and P(AA-AMPS) were closer to that of PVA.

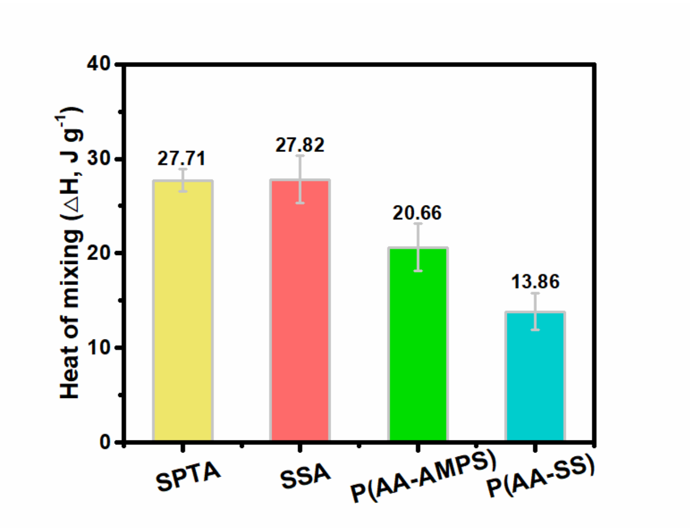

(a)

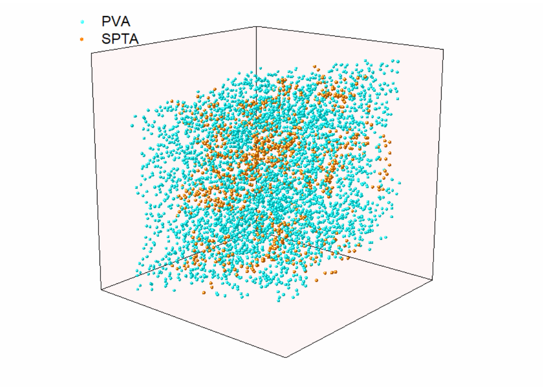

(b) ω_C of SPTA = 30.8 wt.%

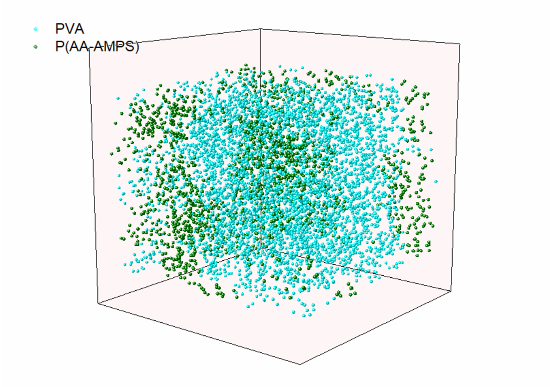

(c) ω_C of P(AA-AMPS) = 32.0 wt.%

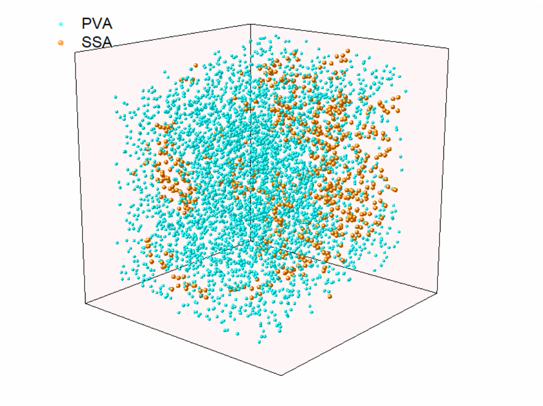

(d) ω_C of SSA = 27.0 wt.%

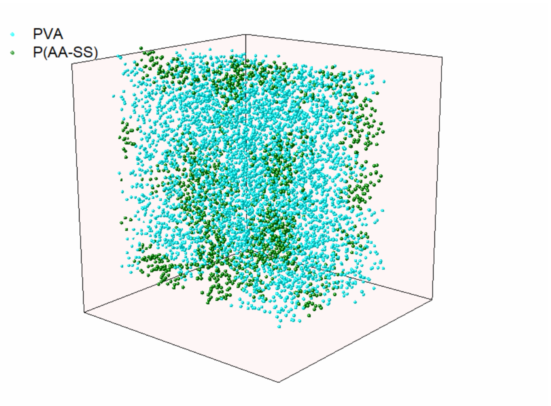

(e) ω_C of P(AA-SS) = 28.4 wt.%

Supplementary Figure 5: (a) ΔH of all the PVA/crosslinkers blend systems at 300 K. (b-e) The atomic spatial distributions of the PVA and the crosslinker molecules in the mixed cells: blue points, PVA atoms; orange points , SPTA and SSA atoms; green points, P(AA-AMPS) and P(AA-SS) atoms. (lines with double cap are error bars, mean ±s.d. for n = 3)

Radial distribution functions (RDFs)

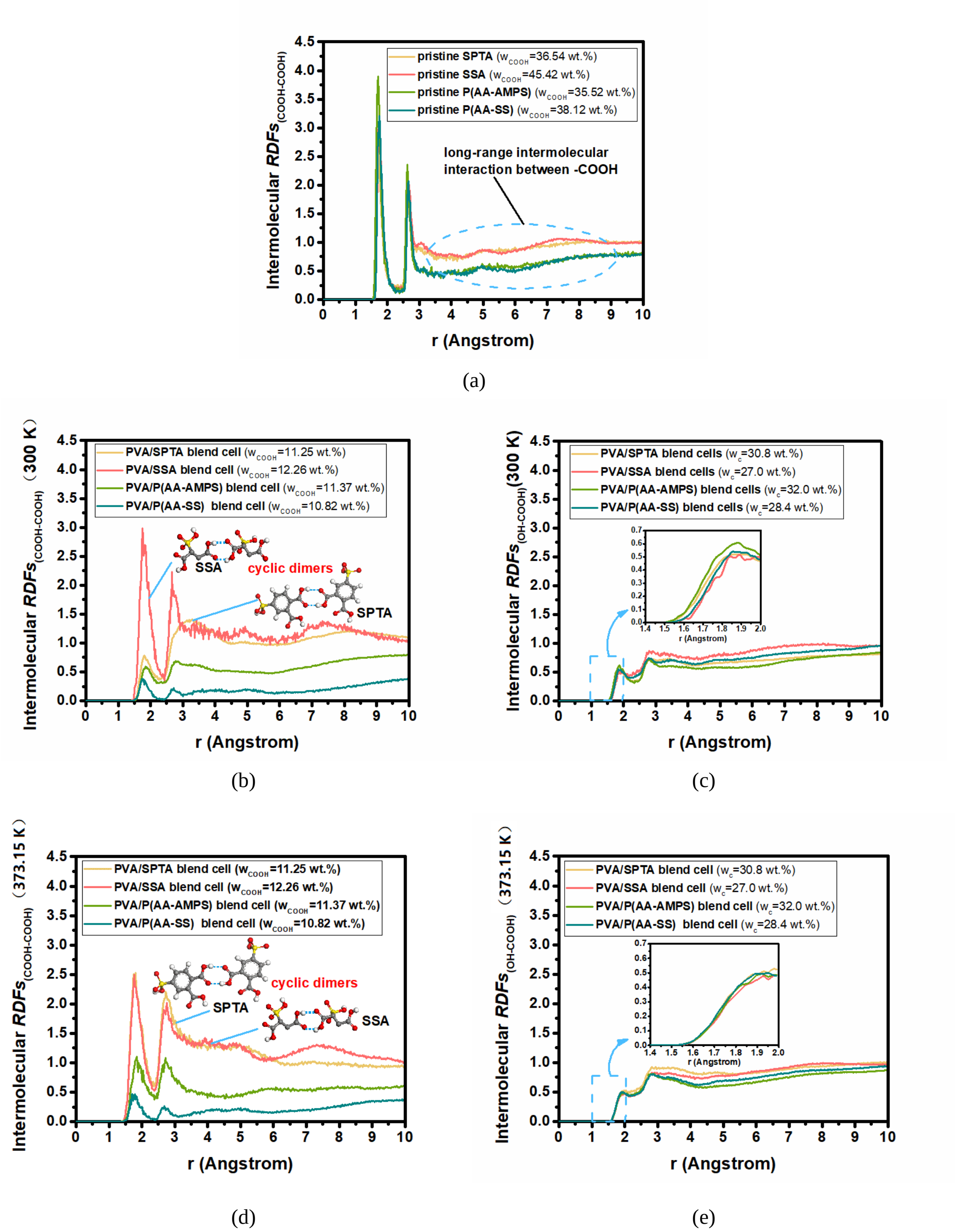

Supplementary Figure 6: The intermolecular RDFs of (a) COOH-COOH for the pure crosslinkers at 300 K, (b) COOH-COOH for the PVA/crosslinkers systems at 300 K, (c) OH-COOH for the PVA/crosslinkers systems at 300 K, (d) COOH-COOH for the PVA/crosslinkers systems at 373.15 K, and (e) OH-COOH for the PVA/crosslinkers systems at 373.15 K.

Supplementary note 3: The Radial distribution functions (RDFs) [3,11] was used to analyze the interactions of -COOH/-OH and -COOH/-COOH groups in the pure systems and PVA/crosslinker systems. For instance, $RDF_{(OH)-(COOH)}$ (**Supplementary Figure 6c**) indicated the probabilities of finding -COOH groups at varied distances (radius) from -OH, which was calculated using the following

expression:

$$RDF_{(OH)-(COOH)} = \frac{N_{(COOH)}/4\pi r^2 dr}{N/V_{cell}} \quad \textbf{(4)}$$

where V_{cell} was the cell volume; N was the total number of -COOH groups in the system; $N_{(COOH)}$ was the number of -COOH groups appeared in a spherical shell at a region form r to dr to a -OH group that located at the center of the sphere; r was the radius of spherical shell. A high value of RDFs indicated high interactions between targeting groups. **Supplementary Figure 6a** presented the intermolecular RDFs of -COOH groups in the pure systems of 4 crosslinkers. Two sharp peaks at short distances from 1.5 Å to 3.0 Å indicated strong interactions among -COOH groups. This was caused by the hydrogen bonding formed by adjacent molecules. The much lower RDFs at long distances from 3 Å to 10 Å represented the weak long-range interactions between -COOH groups. Since the P(AA-AMPS) and P(AA-SS) molecules had long alkyl chains which diluted the concentrations of -COOH, their long-range interactions of -COOH groups were weaker than those of the SPTA and SSA. According to **Supplementary Figure 6b**, the short-range interactions of -COOH groups in the PVA/P(AA-AMPS) and PVA/P(AA-SS) systems were significantly decreased because the crosslinkers were separated by the PVA molecules. However, the short-range interactions of the PVA/SPTA and PVA/SSA systems were still strong, which indicated weak miscibility between them and PVA. This phenomenon was caused by the formation of carboxyl cyclic dimers among the SPTA or SSA molecules by the -OH···O=C hydrogen bonds[16-18]. According to the Hammett theory[19], sulfonic acid, as a strong electron-withdrawing group, had an electron receptor effect that increased the dissociation degree of COOH⇔COO^-+H^+. Meanwhile, the high molecular electrostatic potential of the C=O group formed the stable intermolecular hydrogen bonds (-OH···O=C) [20] and resulted in the formation of cyclic dimers among the SPTA or SSA molecules. Because of the topological constraints from the PVA chain length, the cyclic dimer appeared to be stable enough to survive at a high temperature of 373.15 K (**Supplementary Figures 6d** and **e**) which was the crosslinking temperature selected in this study. This simulation result matched the reports that the cyclic dimers existed even in high-temperature vapors [21-23].

Materials

Supplementary Table 3. The information of chemicals used in this study

Full name	Abbreviation	Mw (g mol^{-1})	Feature	Supplier
Polyvinyl alcohol	PVA	124,000	hydrolysis degree: 99.4 %	Sigma-Aldrich (USA)
Poly acrylic acid co 2-acrylamido-2-methyl propane sulfonic acid	P(AA-AMPS)	27,000	carboxylic acid concentration: 35.52 wt. %; 30 wt. % in H_2O	Sigma-Aldrich (USA)
Sulphosuccinic acid	SSA	198.15	70 wt. % in H_2O	Sigma-Aldrich (USA)
4-sulfophthalic acid	SPTA	246.19	50 wt. % in H_2O	ACROS (China)
Sulfuric acid	H_2SO_4	98.01	Analytically pure: 98.3 wt. %	Beijing Chemical Works (China)
hydrochloric acid	HCl	36.50	Analytically pure: 36.0 wt. %	Beijing Chemical Works (China)
Ethyl alcohol		46.07	Absolute, 99.5 wt. %	Beijing Chemical Works (China)
Sodium chloride	NaCl	58.44	Analytically pure: 99.5 wt. %	Sinopharm (China)
Chlorinated poly vinyl chloride	CPVC	95,000	K(viscosity) = 71 - 72	YUANYE (China)
Poly (styrene-co-maleic anhydride)	SMA	1,600	Styrene content: 75 mol. %	Sigma-Aldrich (USA)
Poly vinyl pyrrolidone	PVP	50,000	Luvitec®, K(viscosity) = 30	BASF (Germany)
N, N-Dimethylacetamide	DMAc	87.12	Super dried, 99.8 wt. %	J&K Scientific (China)
Poly acrylonitrile	PAN	150,000		Sinopharm (China)
N,N-Dimethylformamide	DMF	73.10	Analytically pure: 98.1 wt. %	Sinopharm (China)
Sodium alginate	-	270,000	Viscosity: 200 ±20 mpa s (1 wt.%, 20 ℃)	Aladdin (China)
Tween 20	-	1227.5	Boiling point: 110 ℃	TianJin GuangFu fine chemical research institute (China)
Sodium dodecyl benzene sulfonate	SDBS	348.48	Analytically pure: 98 wt. %	Aladdin (China)

Supplementary note 4: All chemicals used in this study are listed in **Supplementary Table 3** and used as received. Deionized (DI) water was produced from a lab equipped Millipore ultrapure water system with a conductivity of 5.6 µs cm^{-1} and TOC of 1.02 mg L^{-1}.

The crosslinking/grafting reaction diagram and proposed molecular structures

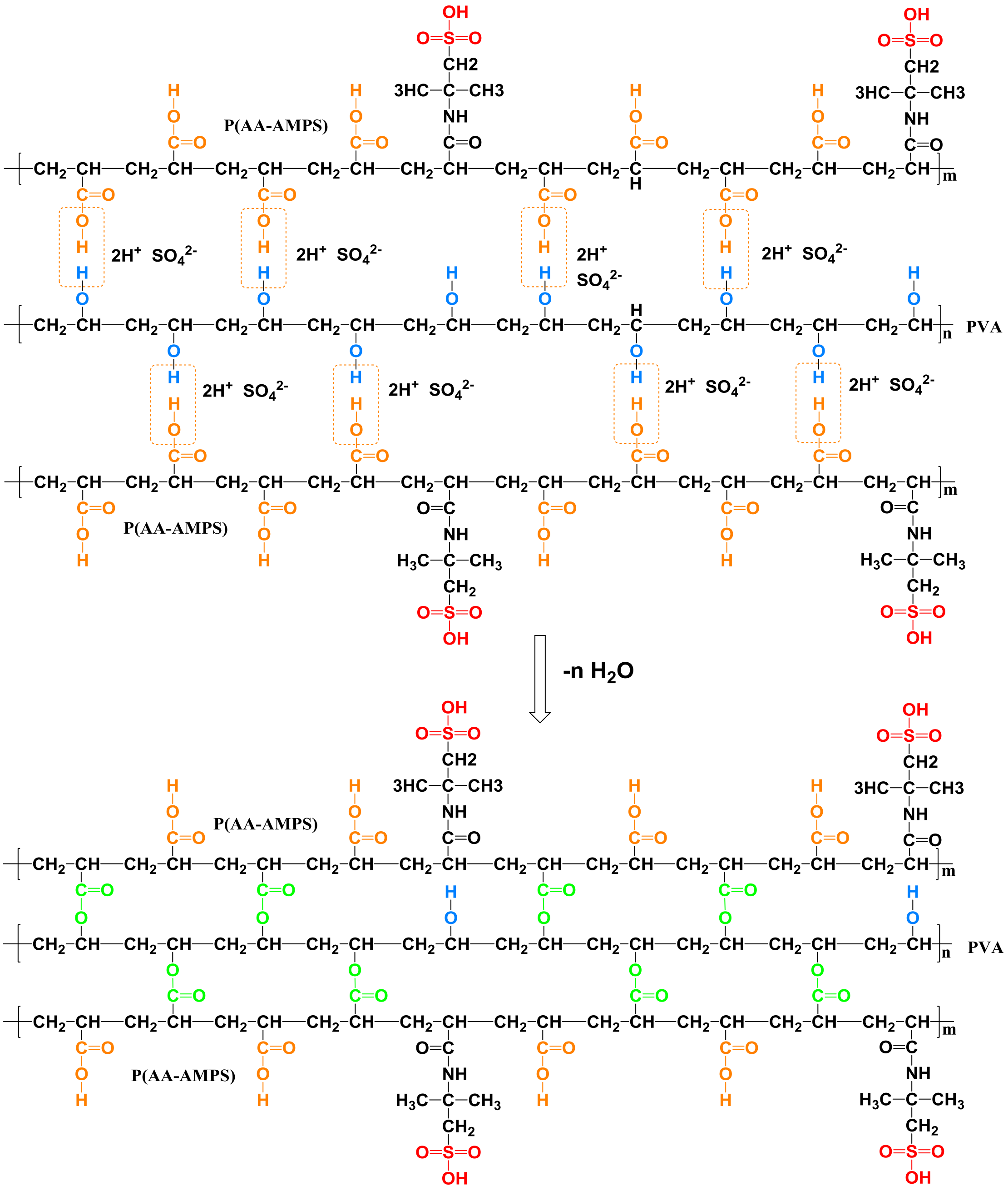

(a)

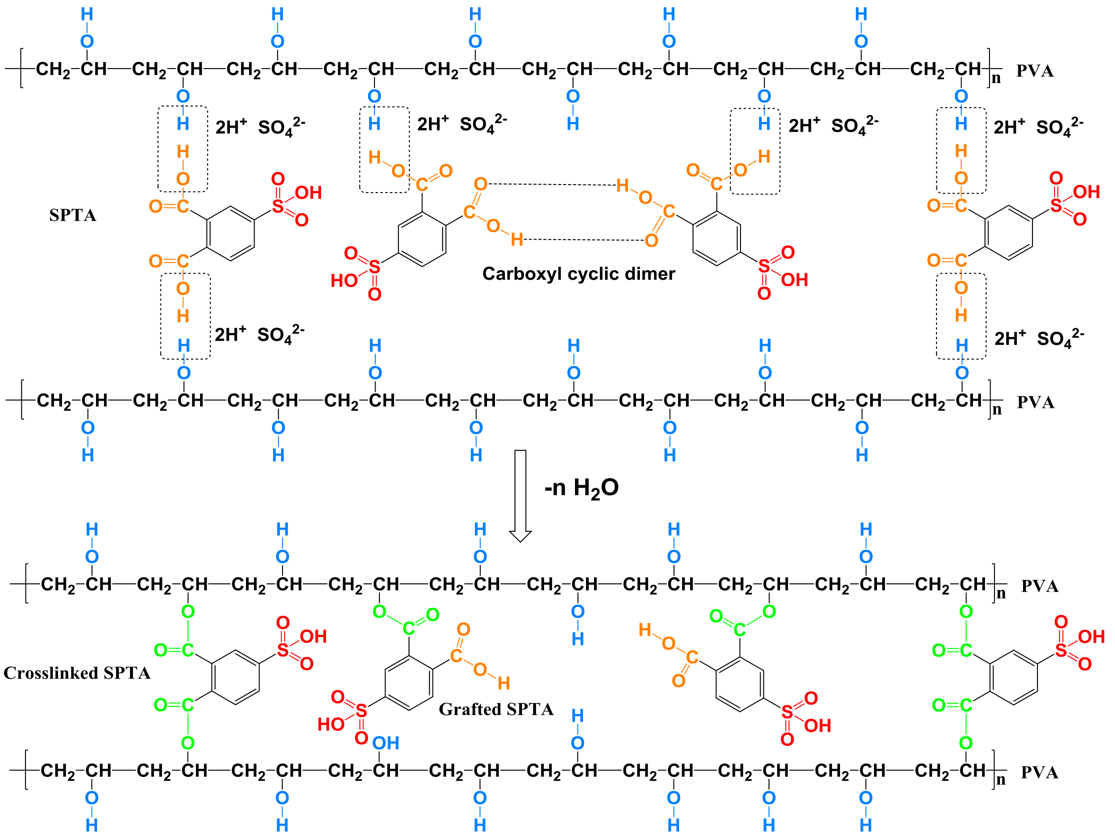
PVA
2H+ SO4 2-
SPTA
Carboxyl cyclic dimer
-n H2O
Crosslinked SPTA
Grafted SPTA

(b)

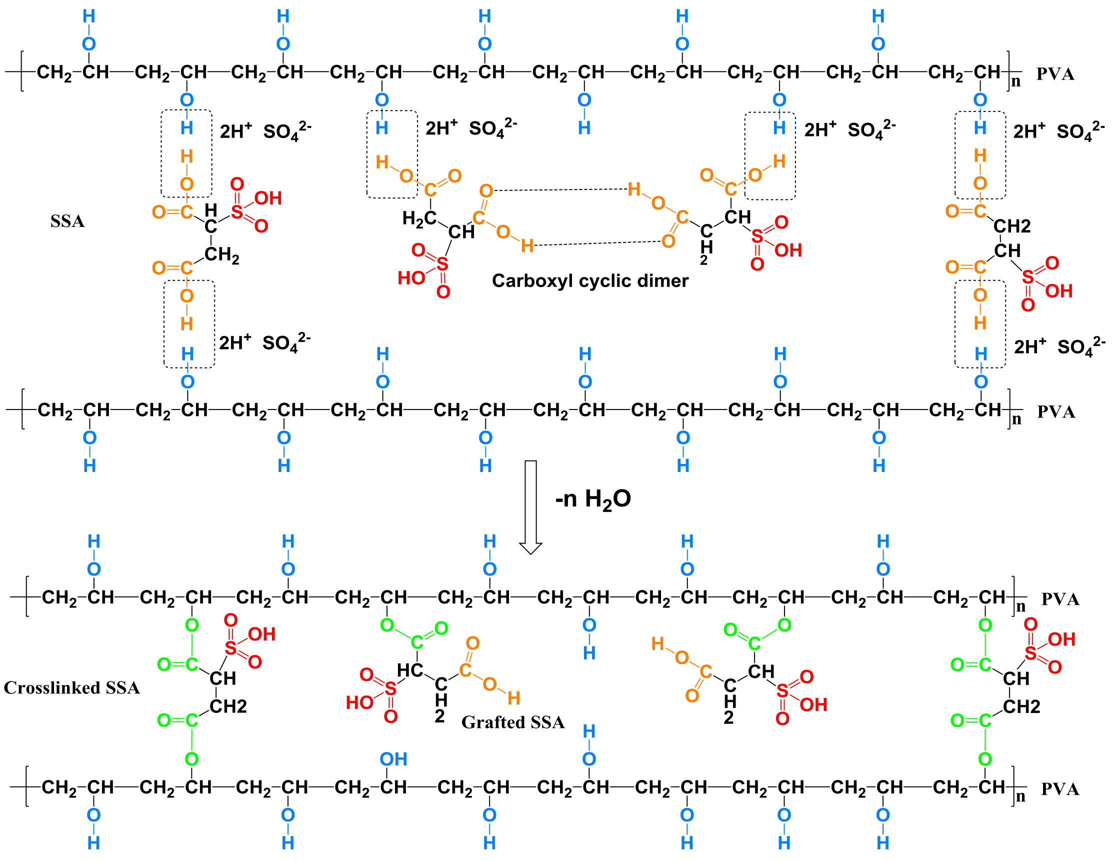

(c)

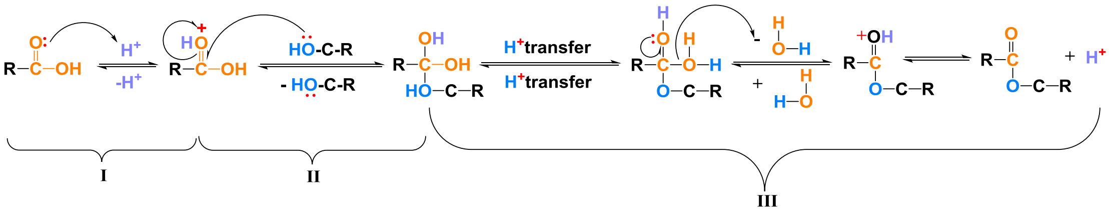

(d)

Supplementary Figure 7: The crosslinking/grafting reaction diagram and proposed molecular structures of the crosslinked PVA-based polymer net-works: (a) PVA/P(AA-AMPS), (b) PVA/SPTA and (c) PVA/SSA. (d) The reaction mechanism of esterification (addition-elimination mechanism).

Acid catalyst optimization

To crosslink the PVA films, -COOH groups of the crosslinkers reacted with -OH groups in PVA via esterification in a solid state. In our previous work [11], the esterification reaction was carried out without acid catalyst. The conversion of esterification was low since the weight loss of the crosslinked PVA were close to 50 wt. % after the drip-washing test. Although increasing the crosslinking temperature could achieve higher esterification conversion, the crosslinking temperature was set at 100 ℃ to prevent PVA from decomposition [11]. In this study, both H_2SO_4 (a non-volatile acid) and HCl were used to test their catalytic efficiency to the PVA/P(AA-AMPS) (85/15 w/w) system. Two PVA/P(AA-AMPS) solutions were prepared at pH=1 by adding H_2SO_4 and HCl, respectively. After drying and subsequently crosslinking the films at 100 ℃ for 15 min, the hydrostabilities of the two films were determined using **drip washing test and weight losses (Supplementary Equation 5)**. The H_2SO_4 catalyzed film had much lower weight loss (2.31 wt.%) than the film catalyzed by HCl (weight loss: 19.10 wt. %) after soaking in an 80 ℃ water bath for 72 h. We also dissolved the PVA/P(AA-AMPS)/acid films without crosslinking treatment in DI water. The pH value of the H_2SO_4 containing system returned to 1, while the HCl containing solution showed a higher pH of 3.27 indicating that most of HCl vaporized during the film formation process. Therefore, H_2SO_4 was a better catalyst to the esterification reaction because of its non-volatility.

Preparation protocols for PVA-based films

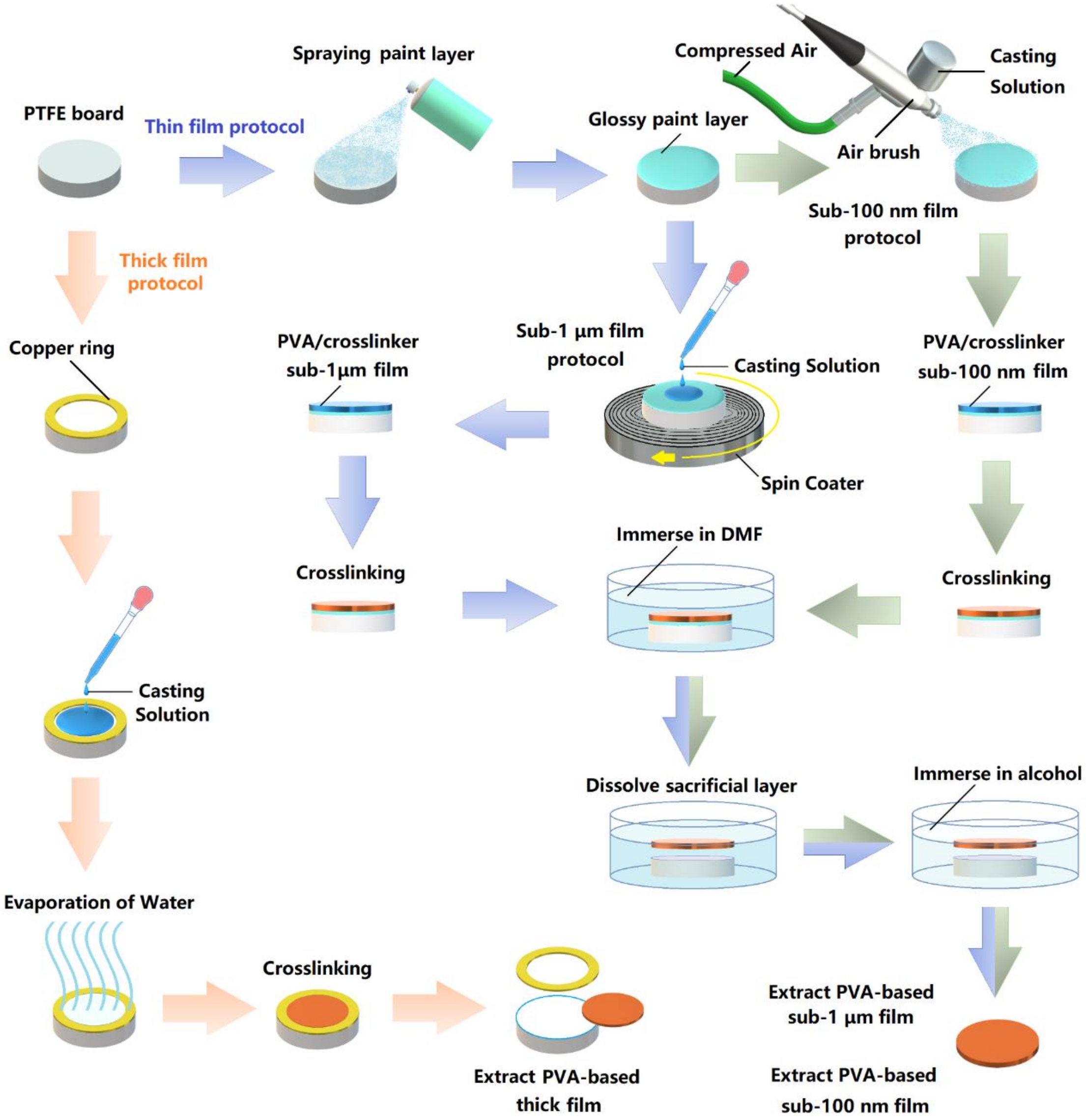

Supplementary Figure 8: The preparation protocols for thick films, sub-1 μm and sub-100 nm PVA-based free-standing films.

Determination of thickness by AFM

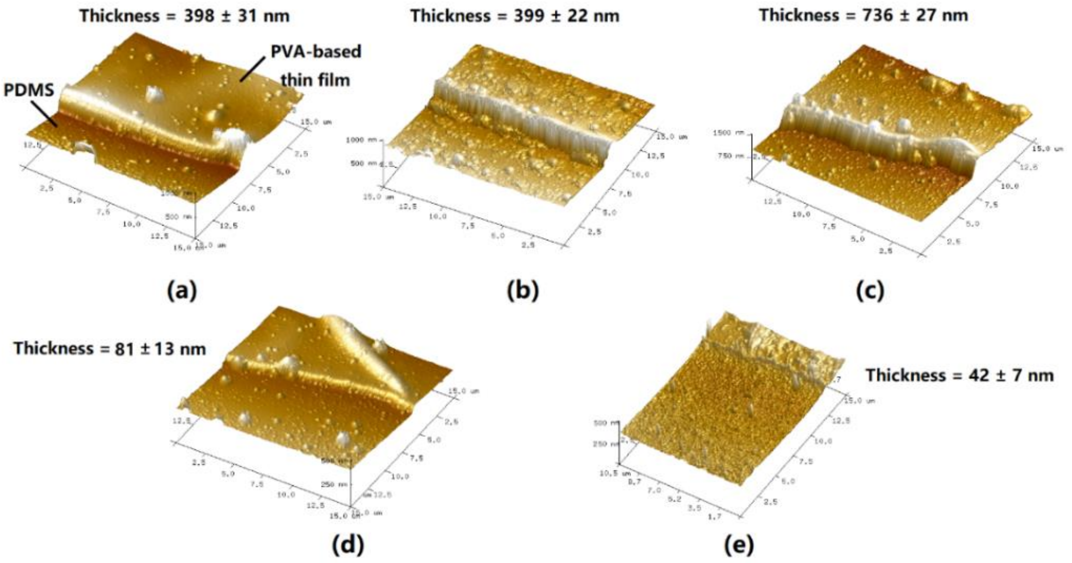

Supplementary Figure 9: Morphology and thickness measurement of the PVA-based free-standing thin films with different thickness: (a) 398±31 nm, (b) 399±22 nm, (c) 736±27 nm, (d) 81±13 nm, (e) 42±7 nm.

Supplementary note 5: The crosslinked PVA thin films were first transferred onto a PDMS substrate and then were observed using AFM (**Supplementary Figure 9**). Thicknesses of thin films were measured by the peak-to-valley method[13] by a NanoScope Analysis 1.80 AFM data analysis software.

Hydrostabilities, swelling degree and crosslinking density

Supplementary Equations 5 and **6** were used to calculate the hydrostabilities and swelling degree (SD) of crosslinked PVA-based films:

$$weight\ loss = \left(1 - \frac{weight\ of\ dry\ films\ after\ drip\ washing}{weight\ of\ dry\ films\ before\ drip\ washing}\right) \times 100\% \qquad (5)$$

$$swelling\ degree\ (SD) = \left(\frac{m_S - m_D}{m_D}\right) \times 100\% \qquad (6)$$

where m_s was mass of the swollen film (g) and m_D was mass of the dried film (g). The measured weight losses are shown in **Supplementary Figure 10a**, and the calculated swelling degree are listed in **Supplementary Table 4.**

Crosslinking density ($\upsilon, \mathrm{mol\ m^{-3}}$) was defined by the moles of polymer segments between the adjacent crosslinking sites [24,25] and calculated using **Supplementary Equation 7**:

$$\upsilon = \rho_p / 2M_c \qquad (7)$$

Supplementary Equation 8, deduced from Flory-Rehner relation[24-28], was used to calculate M_c in **Supplementary Equation 7**

$$M_c = -\frac{\rho_p V_s(\phi^{1/3} - 0.5\phi)}{(ln[1-\phi] + \phi + \chi\phi^2)} \qquad (8)$$

where M_c was the molecular weight (kg mol^{-1}) between crosslinking sites; ρ_p was the density of the crosslinked PVA-based films (kg m^{-3}); V_s was the molar volume of the solvent (m^3 mol^{-1}); ϕ was the volume fraction of polymer in a water-swelled sample and could be calculated by **Supplementary Equation 9** [28]:

$$\phi = \left[(W_d)\rho_p^{-1}\right] / \left[(W_d)\rho_p^{-1} + (W_s)\rho_s^{-1}\right] \qquad (9)$$

where W_d was the weight of the dry crosslinked PVA-based film (kg); W_s was the weight of absorbed water in swollen PVA-based film (kg) and ρ_s was the density (kg m^{-3}) of solvent (water); χ in **Supplementary Equation 8** was the polymer-solvent interaction parameter calculated by **Supplementary Equation 10** [26,29]

$$\chi = 0.44 + 0.18\phi \qquad (10)$$

Densities of the crosslinked PVA-based films were measured using a Mettler PM2000 electronic weighing balance through the displacement method according to ASTM D792-08[30]. Calculated crosslinking density are listed in **Supplementary Table 4.**

Supplementary note 6: The MD simulation results (**Supplementary Figures 3** and **5**) suggested that P(AA-AMPS) and P(AA-SS) were better crosslinkers to PVA because of their good miscibility. In this section, we would verify the conclusion by inspecting the hydrostabilities, swelling degrees, crosslinking densities of the PVA films crosslinked by SSA, SPTA and P(AA-AMPS). P(AA-SS) was not used since it was not commercially available.

The crosslinked thick (52 ± 5 μm) and thin (0.3 - 0.4 μm) films were prepared using the PVA casting solutions at pH=1 with different crosslinkers as listed in **Supplementary Table 4.** Note that, although the weight compositions of varied crosslinkers were different, the mole ratios of -OH: -COOH in the PVA/crosslinker solutions were the same.

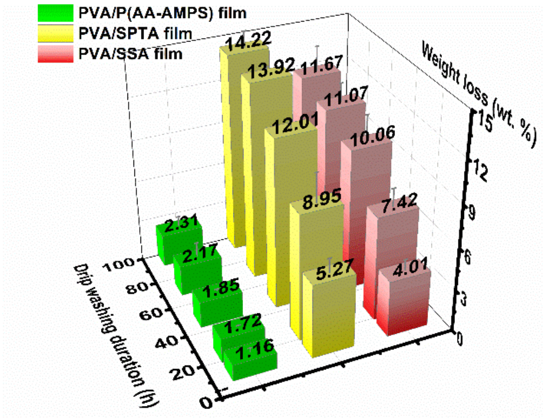

(a)

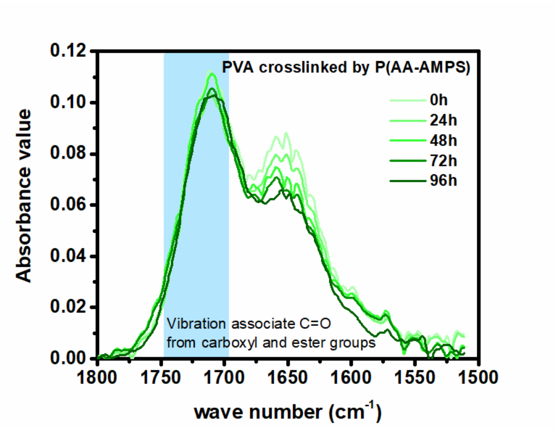

(b)

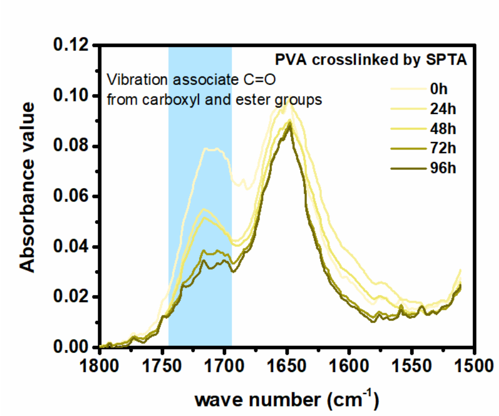

(c)

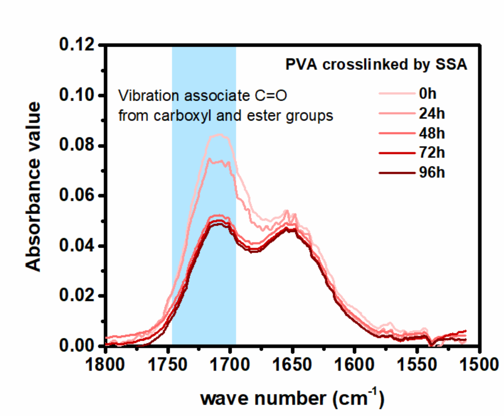

(d)

Supplementary Figure 10: (a) Weight losses of thick PVA-based films during drip-washing test at 80℃ for 4 days, and FTIR spectra change of PVA-based films crosslinked by (b) P(AA-AMPS), (c) SPTA and (d) SSA in drip-washing test. (lines with double cap are error bars, mean ± s.d. for n = 3)

Supplementary Table 4. Compositions and crosslinking density of prepared thick PVA-based films

Crosslinker	Original mass fraction of crosslinker, ω_C (wt. %)	Original mole ratio of (-OH):(-COOH)	Thick films Thickness (μm)	Swelling degree, [a] (%)	Crosslinking density [b] ($\mathbf{mol\ m^{-3}}$)
Pristine PVA	0	-	62 ± 4	-	-
SSA	27.3	6:1	52 ± 5	142.9 ± 7.1	1019.6 ± 101.7
SPTA	31.8	6:1	46 ± 4	163.9 ± 8.3	810.8 ± 76.8
P(AA-AMPS)	30.9	6:1	47 ± 6	128.9 ± 4.4	1291.2 ± 84.1

[a]: Outcomes from swelling tests, calculated by **Supplementary Equation 6**;

[b]: Calculation results from **Supplementary Equation 7**;

-: Pristine PVA film dissolved in aqueous solution.

Supplementary note 7: Hydrostabilities of films were assessed by measuring their weight losses during the drip-washing tests in DI water at 80 ℃. As shown in **Supplementary Figure 10a**, weight losses were fast in the first 12 h, and then became slow, finally stopped after 72 h, indicating a completion of leaching of soluble compounds from crosslinked PVA films[11]. The P(AA-AMPS)

crosslinked PVA film had the best hydrostability since it exhibited the lowest weight loss of 2.31 wt.% compared with that of the SSA and SPTA crosslinked ones of 11.67 wt. % and 14.22 wt. %, respectively. In addition, the peak intensities of the carboxyl and ester groups (1690-1745 cm^{-1} [11]) of SPTA and SSA crosslinked PVA films decreased during the drip-washing tests (**Supplementary Figure 10 c** and **d**), indicating a significantly loss in crosslinkers. While the peak intensities of the P(AA-AMPS) crosslinked films had no obviously change (**Supplementary Figure 10b**). Moreover, the P(AA-AMPS) crosslinked PVA had the highest crosslinking density of 1291.2 ± 84.1 $mol\,m^{-3}$ (**Supplementary Table 4**). In a word, the P(AA-AMPS) crosslinked PVA had the best hydrostability and the highest crosslinking density.

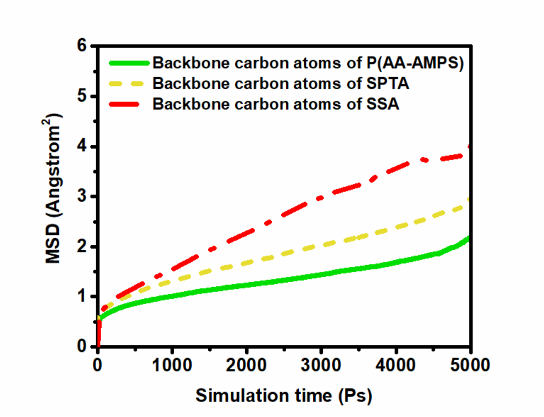

(a)

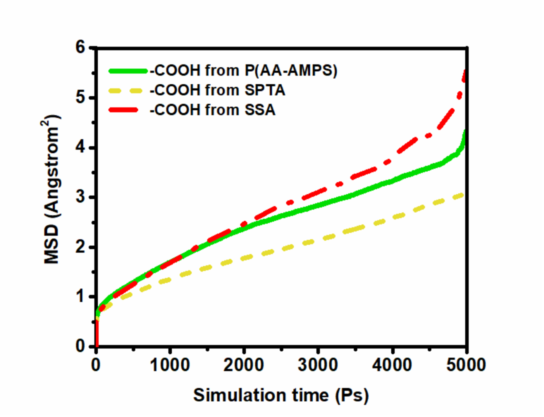

(b)

Supplementary Figure 11: Mean-square displacement (MSD) of (a) backbone carbon and (b) -COOH atoms for SSA, SPTA and P(AA-AMPS) in simulative blend cells, at 373.15 K, 0.01 MPa.

Supplementary note 8: The acid catalyzed esterification reaction could be explained by the addition-elimination mechanism as shown in **Supplementary Figure 7d**. The reaction involved three steps [31]: (I) protonation of carboxylic acid group, (II) formation of intermediates by the attack of the nucleophilic -OH to the protonated carbonyl group, and (III) the generation of ester and water by the decomposition of the intermediate. To obtain high esterification conversion, -COOH groups should have high mobility to reach -OH groups. To compare the mobility of -COOH groups of the P(AA-AMPS), SSA, and SPTA crosslinkers at the crosslinking temperature of 373.15 K, the mean-square displacement (MSD) curves, the slope of which reflected the mobility of selected atoms, were generated by the MD trajectory. **Supplementary Figure 11a** shows that the backbone mobility decreases in an order of SSA>SPTA>P(AA-AMPS). This was due to that the long polymer chain of P(AA-AMPS) restricted its mobility and the rigid benzene ring limited the rotation freedom of SPTA. **Supplementary Figure 11b** shows that -COOH groups in P(AA-AMPS) have higher mobility than SPTA. This could be explained by the long flexible backbone of P(AA-AMPS) and the large side groups in the AMPS block enlarged inter-chain spacing so that weakened inter-chain interactions and decreased the intersegmental friction [32]. Note that, although the -COOH groups of SSA had better mobility than P(AA-AMPS), the formation of cyclic dimers of SSA and relatively poor compatibility with PVA might still led to lower esterification conversion than the PVA/P(AA-AMPS) system.

Mechanical properties of thick PVA-based films

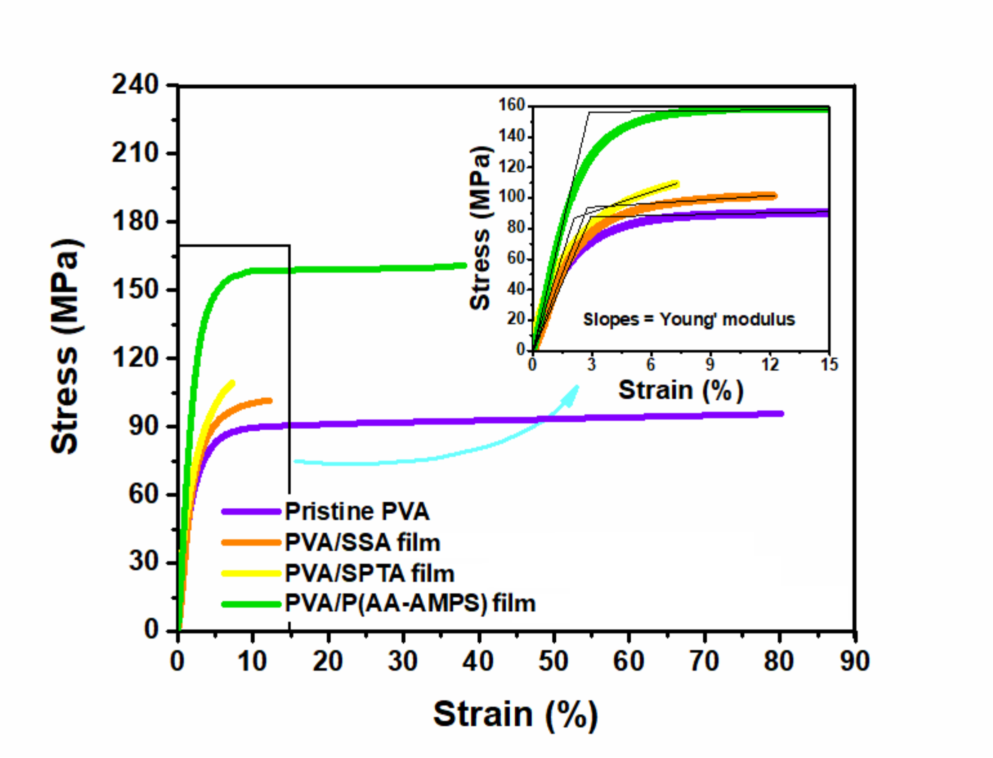

Supplementary Figure 12: The stress-strain curves for thick PVA-based films measured using a DMA equipment.

Supplementary Table 5. Mechanical properties of thick PVA-based films

Sample name	Young's modulus from the tensile test, (MPa)	Tensile strength, (MPa)	Tensile elongation, (%)
Pristine PVA	3746 ±93	96 ±7	79.09 ±5.67
PVA/SSA	4179 ±134	106 ±15	10.18 ±3.11
PVA/SPTA	4560 ±76	109 ±13	4.25 ±1.62
PVA/P(AA-AMPS)	4995 ±132	175 ±28	35.48 ±4.73

Supplementary note 9: Polymer crosslinking typically increased Young's modulus, tensile strength, but decreases ductility. As shown in **Supplementary Figure 12** and **Supplementary Table 5**, both the Young's modulus and tensile strength increased in an order of PVA < PVA/SSA<PVA/SPTA<PVA/P(AA-AMPS). The higher stiffness and tensile strength of the P(AA-AMPS) crosslinked PVA were in accordance with its higher crosslinking density than SSA and SPTA as shown in **Supplementary Table 4**. Although all crosslinked PVA showed decreased ductility, the elongation of break to the P(AA-AMPS) crosslinked PVA (35.48 ± 0.10 %) was much better than those crosslinked by SSA (10.18 ± 0.12 %) and SPTA (4.25 ± 0.33 %). This was due to that the long-chain crosslinker formed polymer coils in the crosslinking networks which could be stretched along with the polymer matrix.

Wrinkling and cracking tests for swollen PVA-based thin films

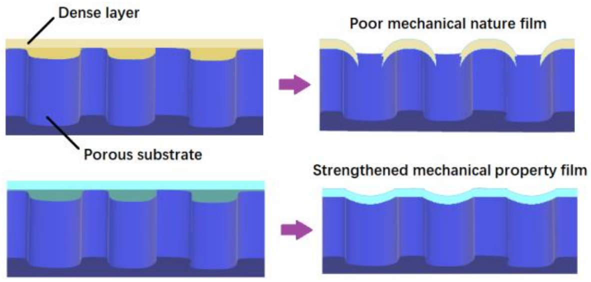

(a)

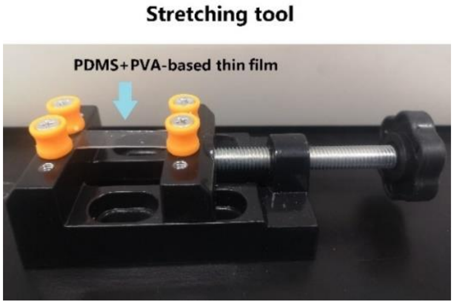

(b)

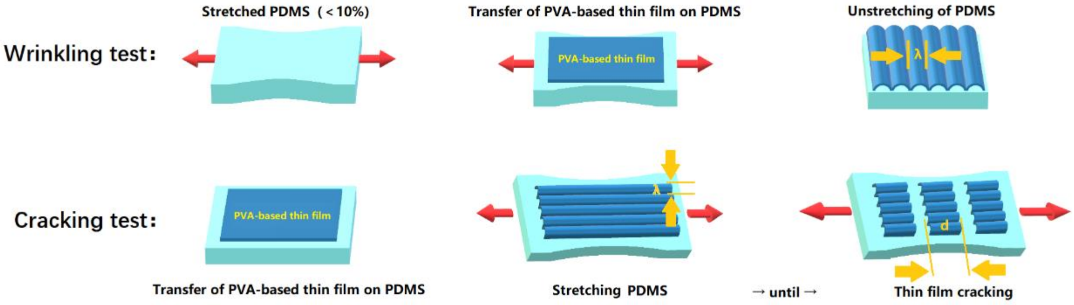

(c)

Supplementary Figure 13: (a) Schematic diagrams of the dense layer's deformation on porous substrate, (b) photograph of the stretching tool used as an elongation holder in wrinkling and cracking test, (c) schematic presentation of wrinkling and cracking test for swollen PVA-based thin films.

Supplementary note 10: Water flux of PV desalination composite membranes was affected by the resistances of both the porous substrate and the dense top layer [33]. High permeable substrates typically had large surface pores. This required that the top layer had good enough mechanical property to avoid rupture as shown in **Supplementary Figure 13a**. Mechanical properties including Young's modulus, fracture strength and onset fracture strain of the water swollen films with thickness below 1 μm were determined from the strain-induced wrinkling[13,34] and cracking[34,35] tests.

The Young's modulus (E_f, MPa) of the thin film could be calculated based on the wrinkling wavelength (λ, μm) using **Supplementary Equation 11**[13,35]:

$$E_f = \frac{3E_s(1-v_f^2)}{(1-v_s^2)}\left(\frac{\lambda}{2\pi h_f}\right)^3 \quad \textbf{(11)}$$

where h_f was the film thickness (μm), and ν was the Poisson's ratio (subscripts f and s denoted the thin film and substrate, respectively). The PDMS substrates with a thickness of 1.3-1.6 mm were prepared by casting a liquid silicon rubber (RTV silicone rubber 705, NANDA, China) on a PTFE plate and then cured at ambient condition for 24 h. Young's modulus (E_s, MPa) of the PDMS substrates (1.82 ± 0.21 MPa) was measured using a DMA equipment. The Poisson's ratios of PDMS (v_s) and PVA (v_f) were set at 0.49 [13] and 0.44 [36] respectively.

The Wrinkle patterns of PVA-based films were shown in **Supplementary Figure 14** and the values of E_f (MPa) were listed in **Supplementary Table 6**. As listed in **Supplementary Table 5**, the Young's modulus of the dry free-standing crosslinked PVA thick films were in the range of 4179 MPa to 4995

MPa. While the Young's modulus (E_f) of the water-swollen PVA-based thin films determined by wrinkling test were in a range of 923 to 2876 MPa (**Supplementary Table 6**). The 1.7-5.4 times lower stiffness of the thin films were caused by the water plasticization. Nevertheless, the P(AA-AMPS) crosslinked PVA thin film showed 3 times higher Young's modulus (2876 ± 147 MPa) than the thin films crosslinked by SSA and SPTA (923 ±179 MPa and 994 ±115 MPa, respectively) in wet state. Therefore, the P(AA-AMPS) crosslinked thin film could bear a high trans-membrane pressure when it was coated on a porous substrate [34].

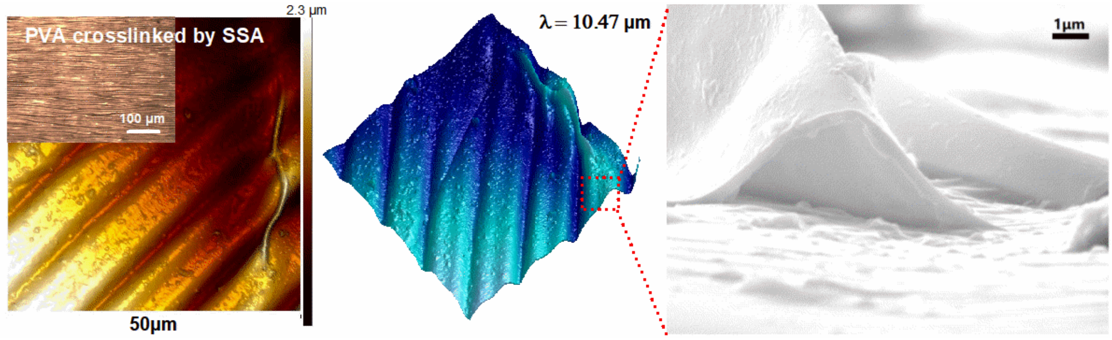

(a)

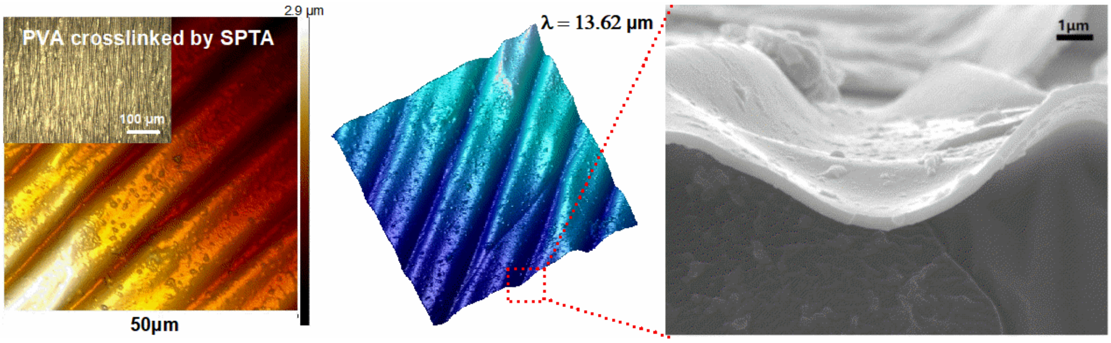

(b)

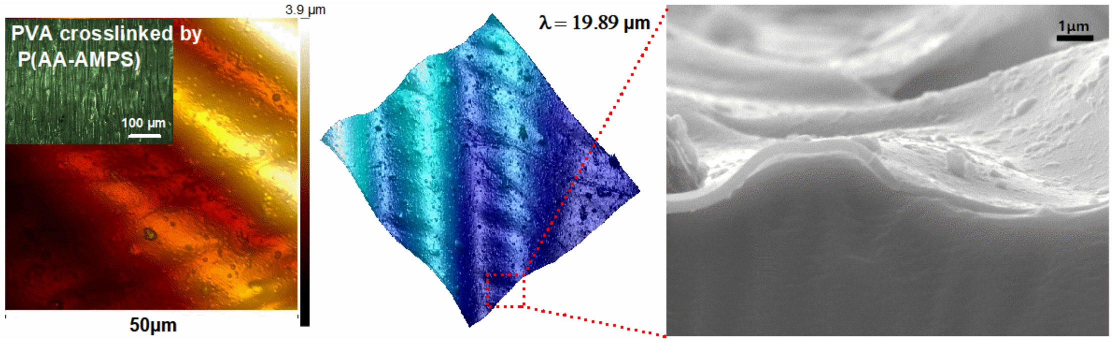

(c)

Supplementary Figure 14: Wrinkle patterns were observed under OM and AFM, the thickness of PVA-based thin films were determined by the cross-sectional FE-SEM images. PVA-based thin films were crosslinked by (a) SSA, (b) SPTA and (c) P(AA-AMPS) at 100 ℃ for 15 min.

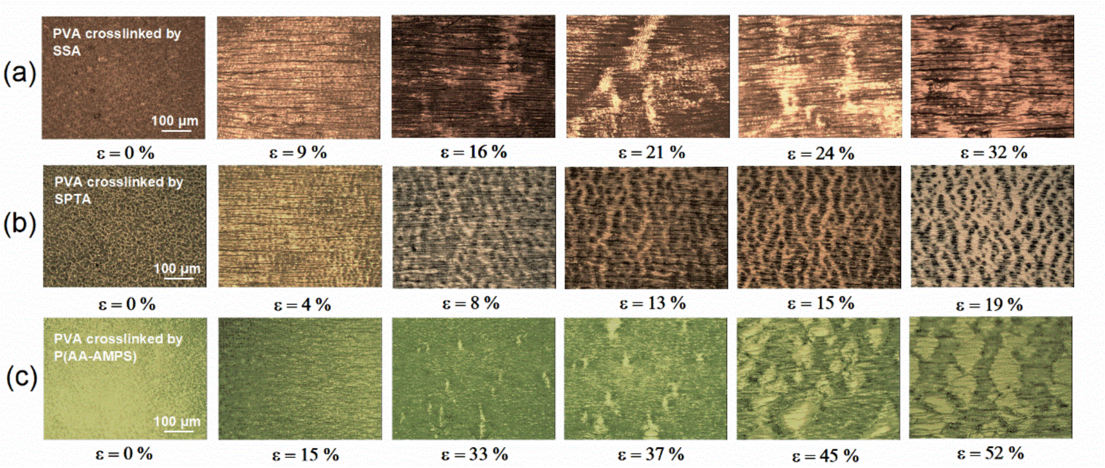

Supplementary Figure 15: The OM images taken at different applied strains (ε, %) for the PVA thin films crosslinked by (a) SSA, (b) SPTA and (c) P(AA-AMPS). The cracks' regions are in light color and the dark regions represent films.

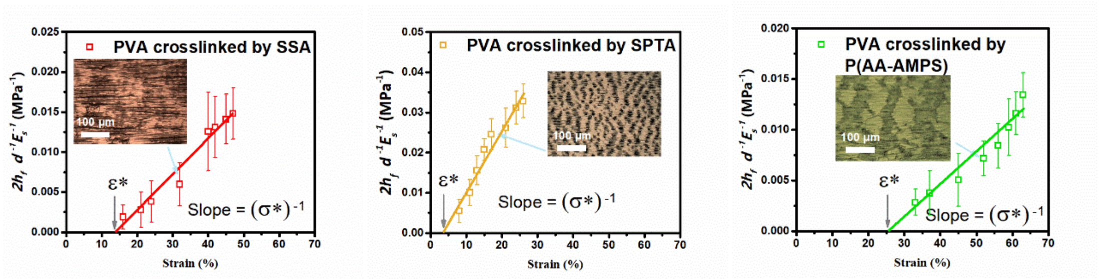

(a) (b) (c)

Supplementary Figure 16: Relations of the crack densities ($2h_f\ d^{-1}\ {E_s}^{-1}$) with the applied strain (ε) for the water-swollen PVA thin films crosslinked by (a) SSA, (b) SPTA and (c) P(AA-AMPS). The solid fitting lines are based on the linear strain-dependent crack density model as described by **Supplementary Equation 12**[35]. (lines with double cap are error bars, mean ± s.d. for n = 3)

Supplementary note 11: Fracture resistance of a composite membrane could be assessed by determining the onset fracture strength (σ^*) and onset fracture strain (ε^*) of the thin top layer[34,35]. The cracking testing procedure was illustrated in the bottom images of **Supplementary Figure 13c**. Specifically, a water-swollen thin film was placed on a PDMS substrate and slowly stretched. The film first buckled periodically at the lateral direction because of the shrinkage of the PDMS substrate and then cracked when further stretched the sample. As shown in **Supplementary Figures 15a (**ε=9%**),b (**ε=4%**), and c(**ε=15%**)**, the periodic wrinkling patterns appeared parallel to the stretching direction [34,35]. At higher strains, the film began to crack orthogonal to the strain direction. Distances between adjacent cracks, denoted as the crack spacing (d, μm), were similar and inversely proportional to strain [35]. By plotting the average crack spacing (d, μm) as a function of strain (ε, %), both the onset fracture strength (σ^*, MPa) and the onset fracture strain (ε*, %) of the films could be quantitative analyzed by **Supplementary Equation 12**[34,35]:

$$\varepsilon(\sigma^*)^{-1} = \frac{2h_f}{dE_s} \qquad \textbf{(12)}$$

where ε was the applied strain (%) recorded during the cracking test. The average crack spacing (d, μm) was measured by the OM image, as shown in **Supplementary Figure 15**. **Supplementary Figure 16** shows the relation of the crack density ($2h_f\ d^{-1}\ {E_s}^{-1}$, MPa^{-1}) to applied strain (ε, %) for the crosslinked PVA thin films. Slopes of the fitting lines ($(\sigma^*)^{-1}$, MPa^{-1}) were used to calculate the onset fracture strength (σ^*, MPa), and the intercepts to the x-axis referred to the onset fracture strains (ε*, %). Values of the onset fracture strain (ε*, %) and fracture strength (σ*, MPa) were listed in **Supplementary Table 6**.

Supplementary Table 6. Mechanical properties of the water-swollen crosslinked PVA thin films

Crosslinker	Wavelength of the wrinkling pattern, **λ** (μm)	Thickness of thin film, $\boldsymbol{h_f}$ (nm)	Young's modulus of swollen thin film, $\boldsymbol{E_f}$ (MPa)	Onset Fracture strength, $\boldsymbol{\sigma^*}$ (MPa)	Onset fracture strain, $\boldsymbol{\varepsilon^*}$ (%)
SSA	13.62 ±0.21	398 ±31	923 ±179	21.83 ±1.06	14.41 ±2.11
SPTA	10.47 ±0.15	295 ±15	994 ±115	6.65 ±2.84	3.06 ±1.76
P(AA-AMPS)	19.89 ±0.76	399 ±22	2876 ±147	31.25 ±4.97	26.25 ±3.57

Supplementary note 12: According to **Supplementary Table 6**, the P(AA-AMPS) crosslinked PVA thin film had the highest onset fracture strength (31.25 ±4.97 MPa) and onset fracture strain (26.25 ± 3.57 %) among three crosslinked films. Therefore, P(AA-AMPS) was selected to crosslink PVA in concerns of its best crosslinking efficiency and mechanical properties.

Loading capacity of crosslinker in P(AA-AMPS)/PVA films

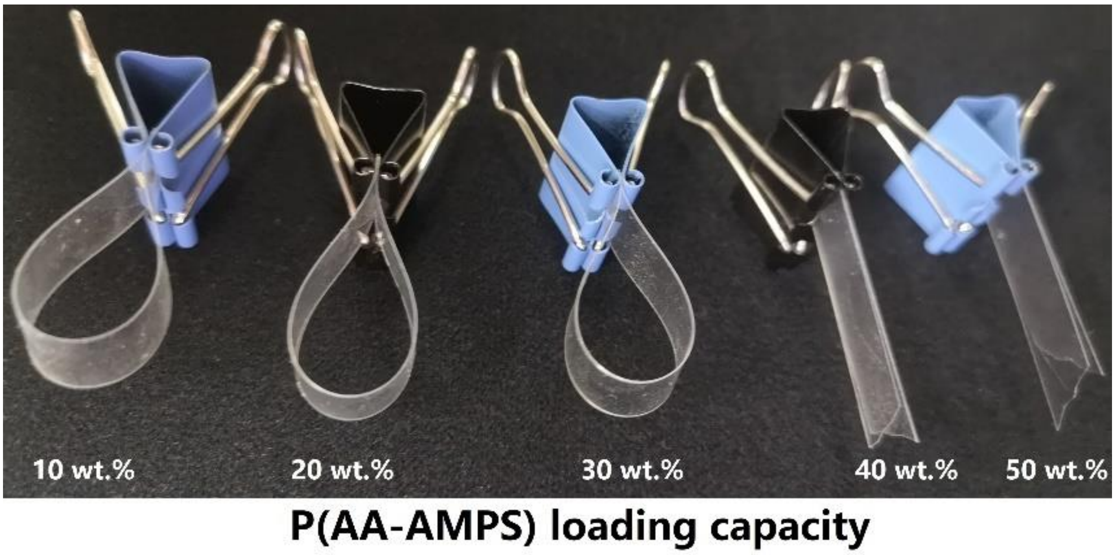

Supplementary Figure 17: The bending capacity of the dry PVA/P(AA-AMPS) films at different P(AA-AMPS) loadings.

Supplementary note 13: A series of crosslinked PVA free standing thick films were prepared with different P(AA-AMPS) concentrations from 10 wt. % to 50 wt. %. As the P(AA-AMPS) concentration increased, the crosslinked films became more and more brittle as shown in **Supplementary Figure 17**. As the P(AA-AMPS) concentration reached to 40 wt.%, the film was easily broken. Therefore, the loading capacities of P(AA-AMPS) in the crosslinking systems were set in a range of 10 to 30 wt. %.

FTIR, TGA, WXRD, and TEM analysis on crosslinked P(AA-AMPS)/PVA films

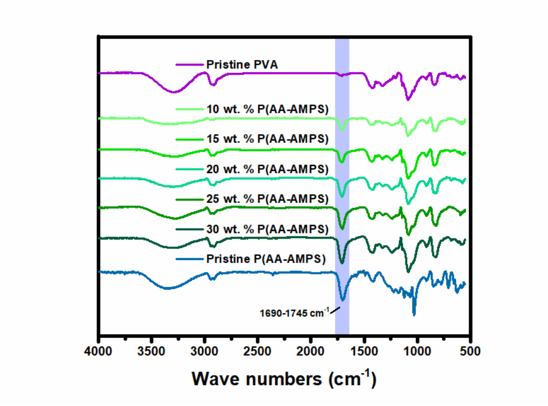

(a)

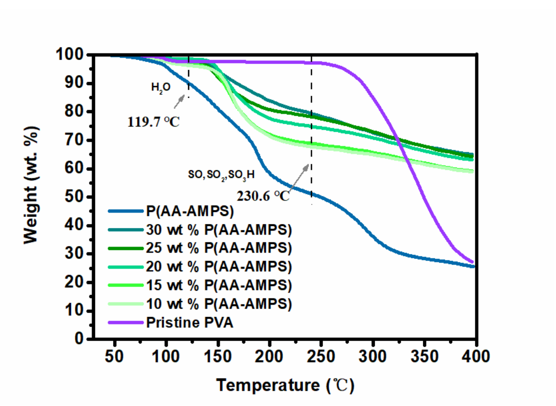

(b)

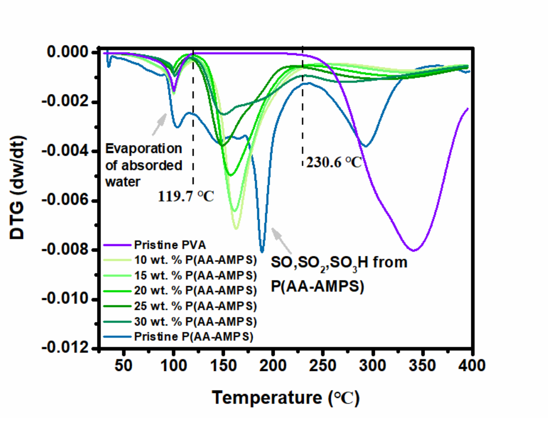

(c)

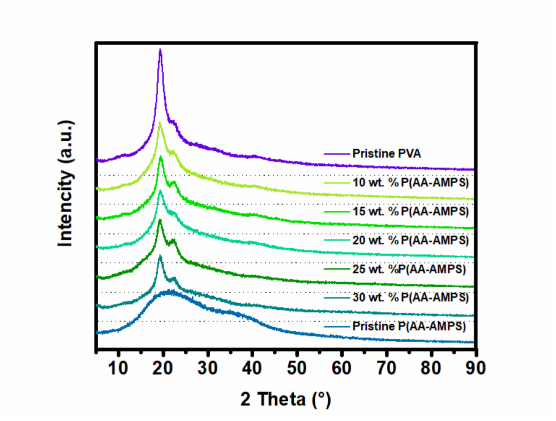

(d)

Supplementary Figure 18: (a) FTIR spectra, (b) TGA, (c) DTG and (d) WAXD curves of the crosslinked PVA/P(AA-AMPS) films compared with pristine PVA and P(AA-AMPS).

Supplementary note 14: Supplementary Figure 18a shows the normalized FTIR spectra of the PVA and crosslinked PVA films. The peak at 1690-1745 cm^{-1} represented the C=O groups of the ester groups and demonstrated the occurrence of esterification. Thermal stabilities of PVA, P(AA-AMPS) and crosslinked PVA/P(AA-AMPS) were evaluated by TGA analysis (**Supplementary Figures 18 b** and **c**). TGA and the 1st derivative (DTG) curves of PVA showed two degradation stages, while the P(AA-AMPS) and crosslinked PVA had three degradation stages. For all specimens, the first weight loss stage between 31 ℃ and 119.7 ℃ was associated with the loss of absorbed water [37]. Since P(AA-AMPS) had sulfonic acid groups that could form strong hydrogen bonding with H_2O[11], the weight loss of P(AA-AMPS) in this region was the highest. The second weight loss stage between 119.7 ℃ and 230.6 ℃ was attributed to the decomposition of SO_2, SO and $-SO_3H$ from the functional groups of P(AA-AMPS) [37]. The pure PVA had no $-SO_3H$ groups and did not show weight loss at this temperature range. The third weight loss stage between 230.6 and 400 ℃ was associated to the decomposition of polymer backbone. Weight loss of the crosslinked PVA decreased as the concentrations of P(AA-AMPS) increased, indicating high crosslinking density improved thermal stability. Since the crosslinked PVA started to decompose at 119.7 ℃, the PV membranes were stable at all testing temperatures of 35-75 ℃ in this work.

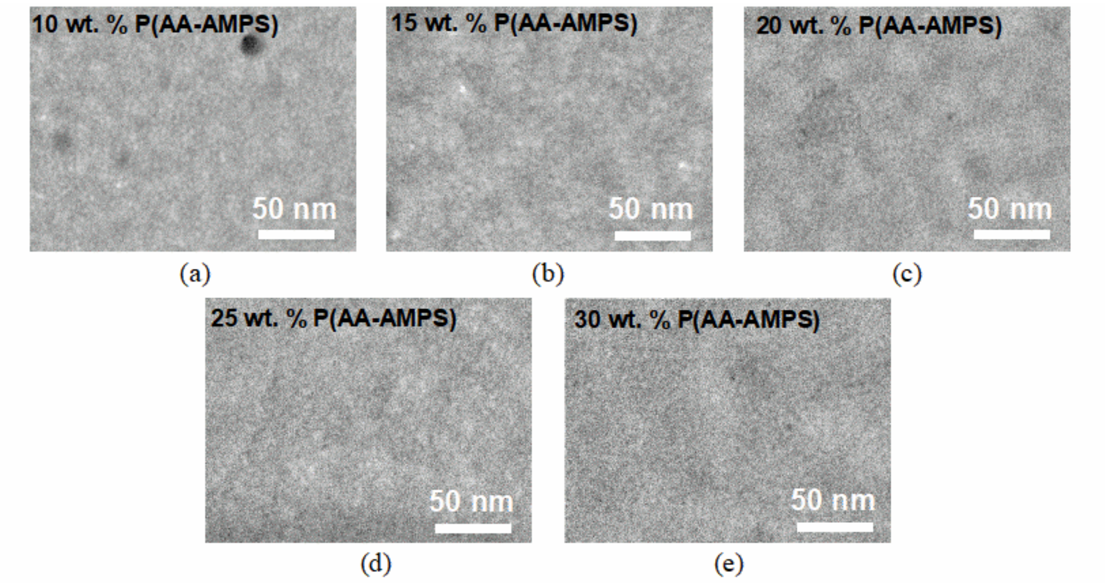

Supplementary Figure 19: High-magnification TEM images of the crosslinked PVA films at a P(AA-AMPS) concentration of (a) 10 wt. %, (b) 15 wt. %, (c) 20 wt. %, (d) 25 wt. %, (e) 30 wt. %.

Supplementary note 15: The WAXD patterns shown in **Supplementary Figure 18d** showed that P(AA-AMPS) was amorphous but PVA had crystalline regions as indicated by the sharp peak at 20 °. The peak intensities decreased with the increases in the crosslinker concentration. It was because high crosslinking structure reduced the crystallinity. High-magnification TEM images of the sub-100 nm PVA/P(AA-AMPS) thin films were shown in **Supplementary Figure 19**. The gray/white pots represented PVA and the small dark dots represented P(AA-AMPS). The uniformly distribution of P(AA-AMPS) demonstrated the good miscibility of the PVA/P(AA-AMPS) system.

Pervaporation desalination test

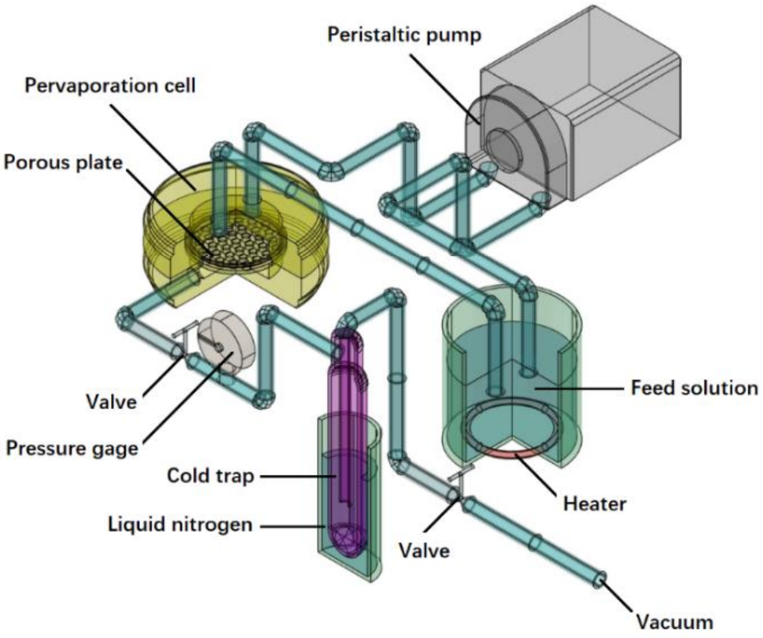

(a)

Supplementary Figure 20: The diagrammatic sketch of pervaporation set-up.

Supplementary note 16: Desalination performance of the PVA-based composite membranes were measured by a bespoke pervaporation set-up [11], as drawn in **Supplementary Figure 20**. In the pervaporation cell, a piece of composite membrane with an effective transport area of 3.28 cm^2 was placed on top of a porous metal plate. A feed solution (NaCl aqueous solution) was pumped into the cell using a Langer® peristaltic pump at 400 r min^{-1} (Re ≈ 4,700) to alleviate the temperature and concentration polarizations. A further increase in rotation rate did not result in any difference in membrane flux. Pressure at the permeate side was maintained at 100 Pa. Water flux (J: Kg m^{-2} h^{-1}) was determined using **Supplementary Equation 13**:

$$J = \frac{M}{S_1 \cdot t_1} \quad (\mathbf{13})$$

where M (kg) was the permeate mass collected every 10 min for 5 times at least in a liquid nitrogen cold trap; S_1 was the effective membrane area (m^2); and t_1 was the experimental time (h). The NaCl rejections (R_{NaCl}, %) were determined by **Supplementary Equation 14**:

$$R_{NaCl} = \frac{C_{feed} - C_{permeate}}{C_{feed}} \times 100\% \quad (\mathbf{14})$$

where C_{feed}, $C_{permeate}$ were the salt concentrations of the feed and permeate solutions. C_{feed} were set as 1.5 wt. %, 3.5 wt. %, 10 wt. % or 20 wt. %, whilst $C_{permeate}$ was determined in an indirect way. After each pervaporation test, the permeate side of composite membranes were washed with DI water. A conductivity meter (Con 110, Thermo Fish OAKTON, Singapore) was used to measure the NaCl concentration of the washing solution ($C_{permeate}$). The long-term desalination and anti-fouling properties of PVA/nanofiber composite membranes were exam by treating a 3.5 wt. % NaCl solution with 0.5 wt. % Tween 20 or sodium dodecyl benzene sulfonate (SDBS) or sodium alginate (bio foulant) acting as an organic foulant.

Preparation of the CPVC porous substrate

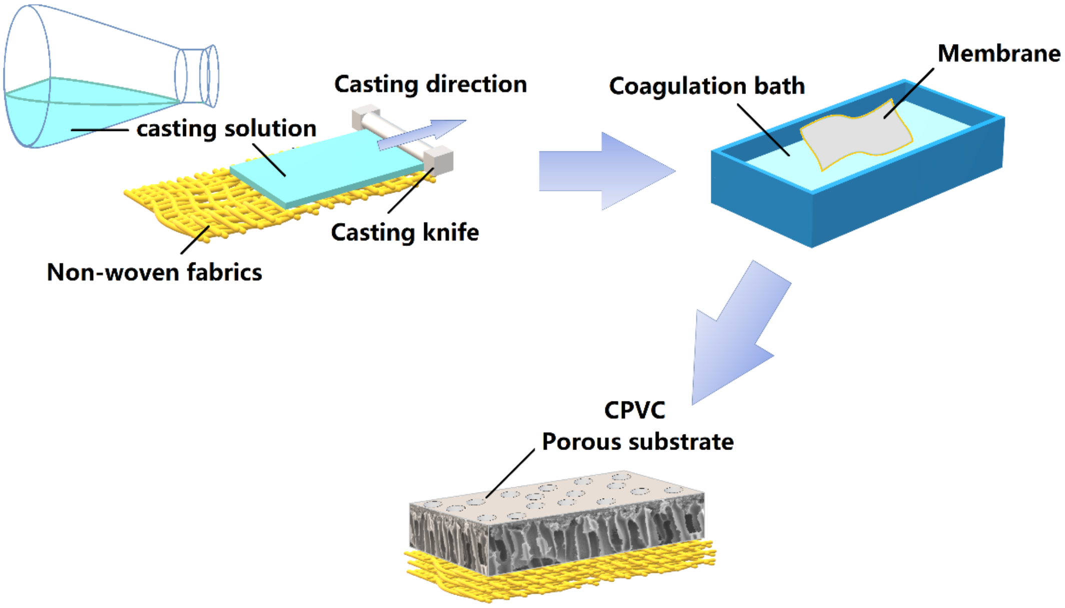

Supplementary Figure 21: Preparation protocol of CPVC ultrafiltration membranes.

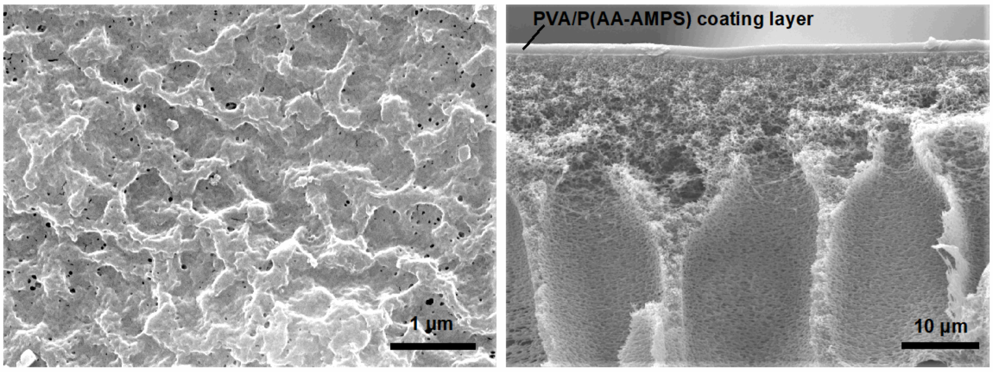

(a) (b)

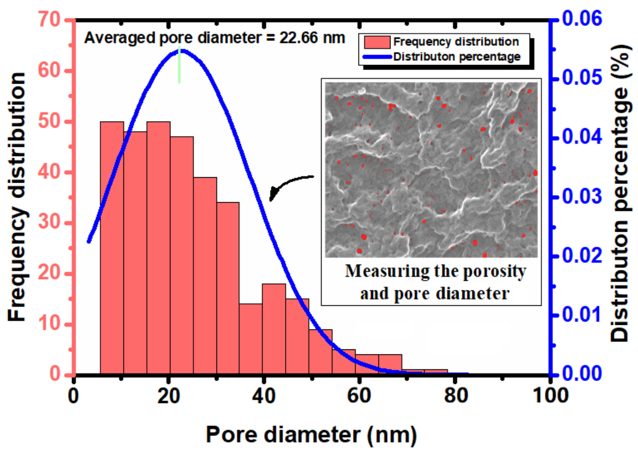

(c)

Supplementary Figure 22: (a) Surface and (b) cross-sectional images of CPVC porous substrate, and (c) pore diameter distribution estimated using analysis software (Image J, Math2 Market GmbH, German) based on the FE-SEM image of the CPVC membrane surface.

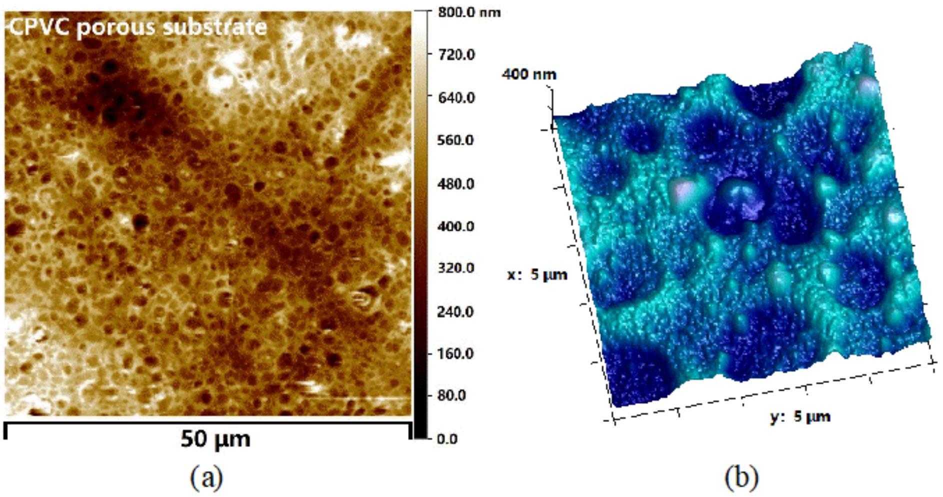

Supplementary Figure 23: The AFM images of the CPVC membrane. (a) 2D surface morphology (50×50 μm^2), (b) 3D surface morphology (5×5 μm^2).

Supplementary note 17: A schematic diagram of the composite membrane fabrication process is shown in **Supplementary Figure 21**. A CPVC dope consisting of CPVC/SMA/PVP/DMAc at a weight ratio of 16/4/4/76 was cast on top of a polyester fabric with a film thickness of 150 μm and then immersed in a deionized water bath at 24 ± 1 ℃ to form the CPVC membrane. The water bath was replaced every 12 h for 3 days to remove the residual organic solvent. After that, the wet CPVC membrane was air-dried at room temperature. The average pore size (22.66 nm) and porosity (7.19%) of the membrane surface were determined using the image J software based on the SEM pictures shown in **Supplementary Figures 22a** and **c.** Cross-sectional image of the CPVC membranes showed finger-like macro-voids beneath a sponge-like porous top layer as shown in **Supplementary Figure 22b**. The roughness of the membrane, measured using AFM, was 400 nm as shown in **Supplementary Figure 23**.

Preparation of the PVA/CPVC composite membranes

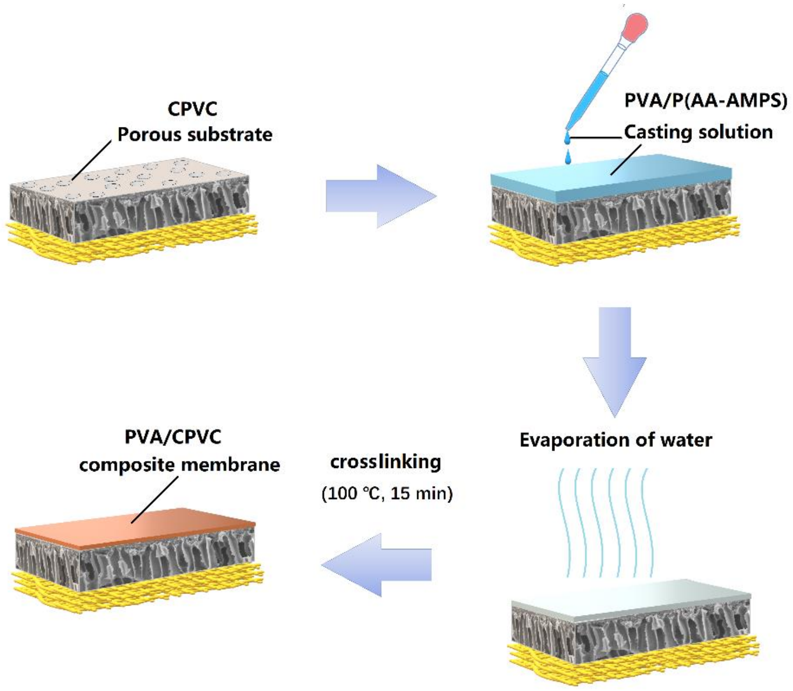

Supplementary Figure 24: Preparation protocol of PVA/CPVC composite membranes.

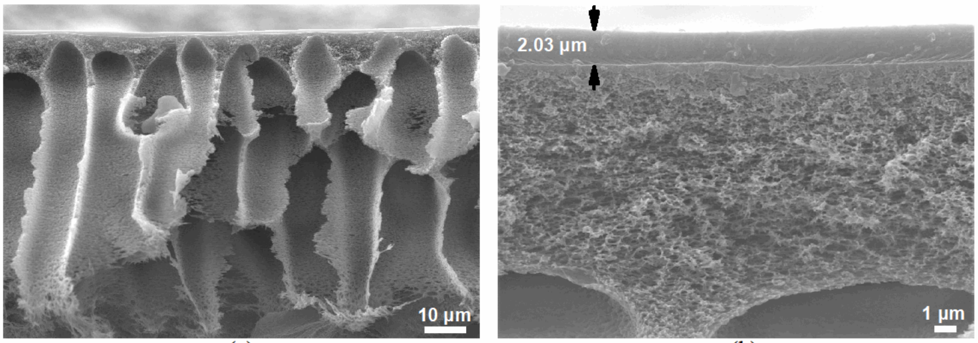

Supplementary Figure 25: Cross-sectional FE-SEM images of (a) CPVC porous substrate and (b) PVA/P(AA-AMPS) coating layer.

Supplementary Table 7. The compositions and coating layer thicknesses of the composite membranes.

Composite membrane code	PVA/P(AA-AMPS) weight ratio (wt. %: wt. %)	Thickness of coating layer (μm)
PVA-10-CPVC	90:10	2.09 ±0.17
PVA-15-CPVC	85:15	1.94 ±0.31
PVA-20-CPVC	80:20	2.13 ±0.17
PVA-25-CPVC	75:25	1.88 ±0.22
PVA-30-CPVC	70:30	2.03 ±0.19

Supplementary note 18: PVA/CPVC composite membranes were prepared using a drip-coating

method as shown in **Supplementary Figure 24**. A detailed description could be found in **Supplementary Reference** [38]. Concentration and pH value of all PVA solutions were 2.5 wt. % and 1, respectively. The P(AA-AMPS) contents were listed in **Supplementary Table 7**. To maintain a constant thickness of the PVA layer, the dosage of the PVA solution was kept at 20 μL per square centimeter by a micropipette. The composite membranes were dried at 25 ℃ for 24 h, crosslinked in a muffle furnace at 100 ℃, and soaked in water at room temperature for 48 h to remove the soluble components and residual acid (H_2SO_4). The crosslinked membranes were labeled as PVA-wt. % of P(AA-AMPS) -CPVC. For example, PVA-10-CPVC represented a PVA/CPVC membrane crosslinked by 10 wt.% P(AA-AMPS). Thicknesses of the PVA and CPVC layers were 2 ± 0.2 μm and 85 ± 10 μm, respectively, as shown in **Supplementary Figure 25**.

Optimization of the crosslinking duration

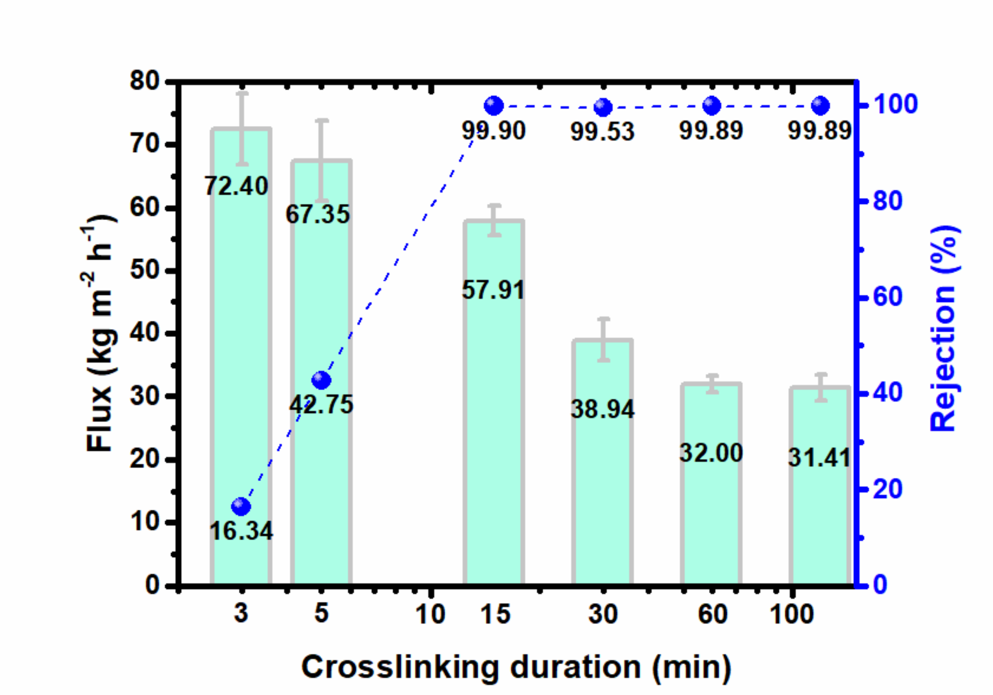

Supplementary Figure 26: Effect of crosslinking duration on the PV desalination performance for the PVA-30-CPVC composite membrane at 70 ℃ using a 3.5 wt. % NaCl feed solution. (lines with double cap are error bars, mean ± s.d. for n = 5)

Supplementary note 19: High speed crosslinking is preferred for the large-scale membrane manufacturing. To determine the shortest crosslinking time of the PVA/P(AA-AMPS) system, the PVA-30-CPVC composite membranes were crosslinked at 100 ℃ for 3-120 min. As shown in **Supplementary Figure 26**, membranes crosslinked for 3 and 5 min had high water fluxes of 72.4 ± 6.1 kg m^{-2} h^{-1} and 67.3 ± 7.2 kg m^{-2} h^{-1}, respectively. But salt rejections were very low (16.34 % and 42.75 %), which was caused by the poor hydrostabilities of the partially crosslinked PVA layers. As the crosslinking time increased to 15 min, high salt rejection of 99.90 % was achieved. This indicated that the crosslinked PVA layer was defect-free. Further increasing crosslinking time only decreased water flux because of the higher crosslinking density. Therefore, 15 min was selected as the optimal crosslinking time.

Effect of the P(AA-AMPS) concentration on membrane water flux

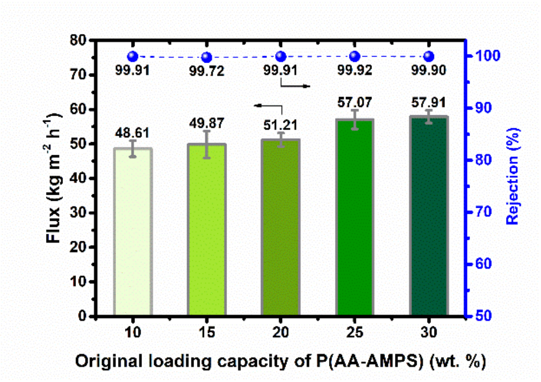

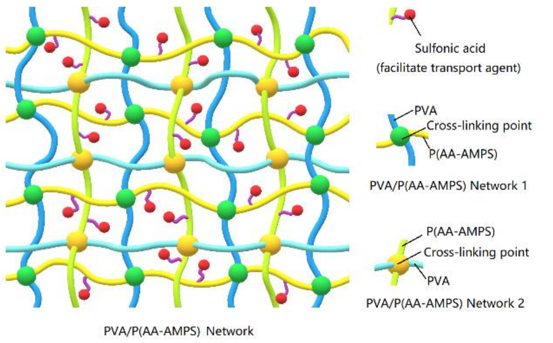

(a) (b)

Supplementary Figure 27: (a) the desalination properties of the PV composite membranes containing different loadings of P(AA-AMPS) crosslinkers (the feed was a 3.5 wt. % NaCl solution at 70 ℃). (b) A schematic network structure for the PVA/P(AA-AMPS) films. (lines with double cap are error bars, mean ±s.d. for n = 3)

Supplementary note 20: A series of PV composite membranes having a similar PVA layer thickness of 2 ± 0.2 μm but different P(AA-AMPS) contents (10 wt. % to 30 wt. %) were prepared. Their desalination properties were tested at 70 ℃ by using a 3.5 wt. % NaCl solution as feed. **Supplementary Figure 27a** shows that all composite membranes have similar salt rejections of 99.87 ± 0.10 %. Water fluxes increased from 48.61 ±2.33 to 57.91 ±1.76 kg m^{-2} h^{-1} as the P(AA-AMPS) contents increased. The increased water fluxes should be attributed to the higher concentrations of sulfonic acid groups (as shown in **Supplementary Figure 27b**) that facilitated the transport of water molecules [37,39]. This phenomenon were also observed in our previously paper for the SPTA crosslinked PVA [39]. Since the PVA-30-CPVC exhibited the best desalination property, the PVA/P(AA-AMPS) (7/3 w/w) coating solution was used for preparing PV composite membranes in the following sections.

Effect of the feed temperature on PV desalination performance

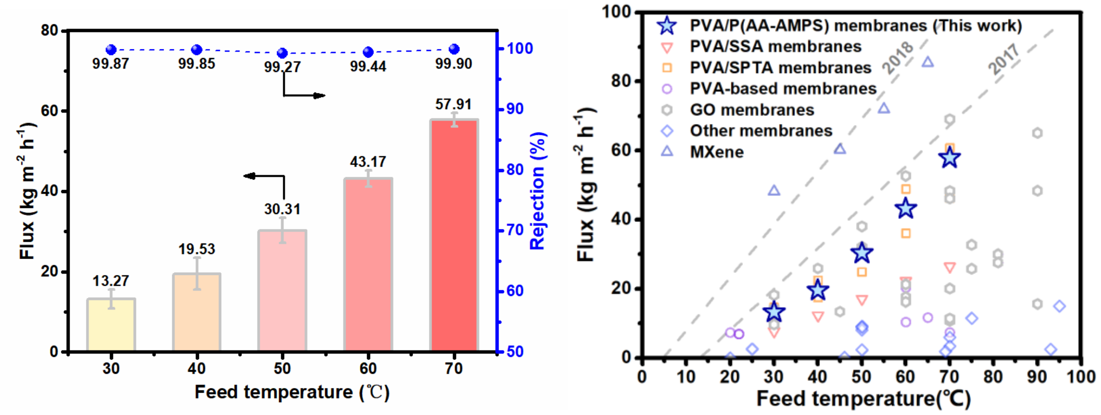

(a) (b)

Supplementary Figure 28: (a) the PV desalination performances of the PVA-30-CPVC at different temperatures for 3.5 wt. % NaCl feed solution. (b) Compare the desalination properties of PVA/CPVC composite membranes with literature data (detailed information is listed in **Supplementary Table 9**, lines with double cap are error bars, mean ±s.d. for n = 5)

Supplementary note 21: Supplementary Figure 28a shows the water fluxes and salt rejections of the PVA-30-CPVC membranes to 3.5 wt. % NaCl solution at the feed temperatures from 30 - 70 ℃. The salt rejections were all above 99.27 % and the water fluxes increased from 11.3 ±2.3 to 57.9 ±1.5 kg m^{-2} h^{-1}. The results were reasonable since high temperature increased the water vapor pressure at the feed side and enhanced the water diffusivity in the crosslinked PVA layer. The high salt rejections suggested that the PVA dense layer was stable in the whole testing temperature range. However, as shown in **Supplementary Figure 28b** and **Supplementary Table 8**, water fluxes of the PVA-30-CPVC membrane were similar to our previous reported SPTA crosslinked PVA/PAN composite membrane but significantly lower than the MXene based PV membrane [40]. This could be caused by the much thicker PVA layer (2μm) than the MXene layer (60 nm). In the following sections, we would show how to increase water flux by optimizing porous substrate structure and decreasing the coating layer thickness.

Preparation of PVA/alumina composite membranes

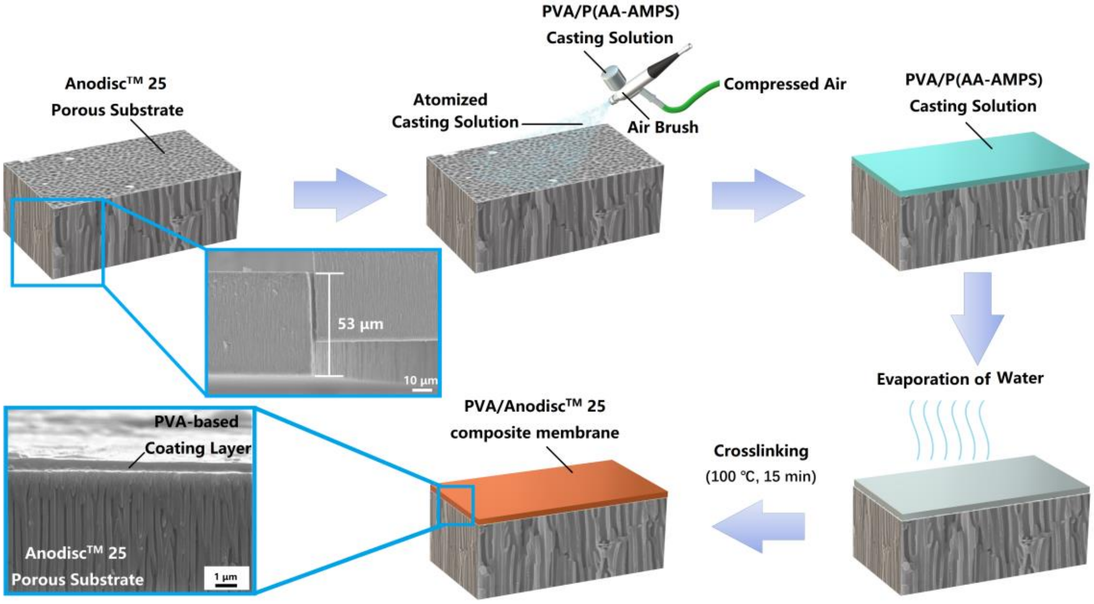

(a)

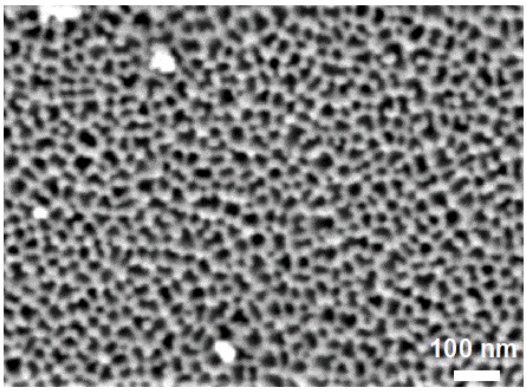

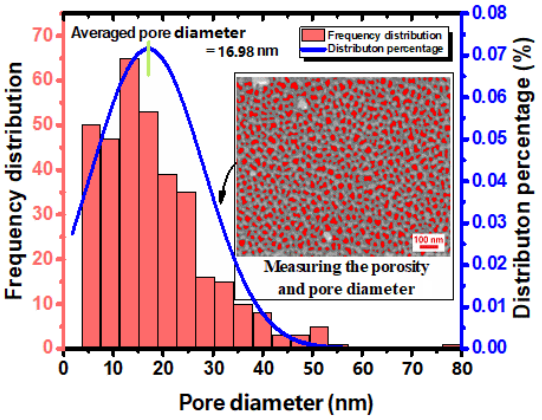

(b) (c)

Supplementary Figure 29: (a) the preparation process of PVA/ alumina composite membranes; (b) the surface FE-SEM images and (c) pore size distribution of the alumina membrane (the software used to determine pore sizes were Image J, Math2 Market GmbH, German).

Supplementary note 22: Porous alumina membrane (Anodisc™ 25, Whatman™, Germany) has been widely adopted as the substrate for preparing composite membranes [41,42]. As shown in **Supplementary Figure 29** and **Supplementary Figure 30**, the alumina membrane had smaller surface pore size (diameter = 16.98 nm) but higher surface porosity (47.10%) than those of the CPVC membrane (22.66 nm and 7.19%). More importantly, surface roughness of the alumina membrane (50 nm) was much lower than that of the CPVC membrane (400 nm). The smoother surface and small pore size of the alumina substrate were preferred for coating defect-free thin film.

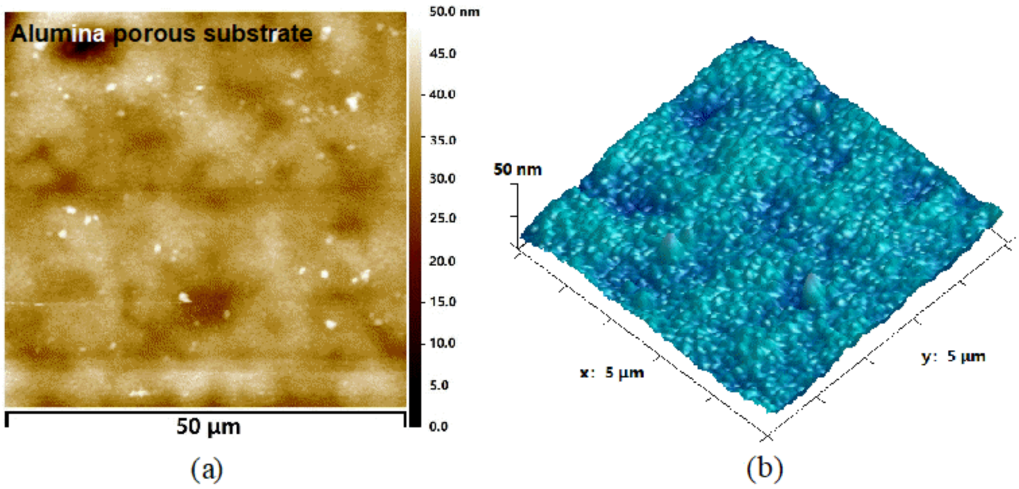

Supplementary Figure 30: The AFM images of the alumina membrane. (a) 2D surface morphology (50×50 μm^2), (b) 3D surface morphology (5×5 μm^2).

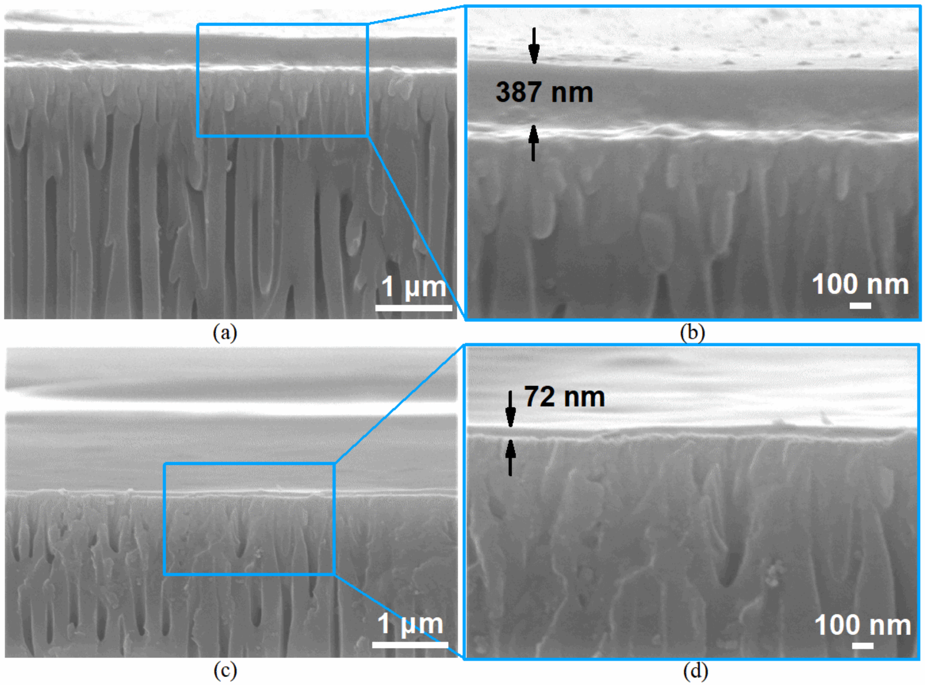

Supplementary Figure 31: Cross-sectional images of the PVA/alumina composite membranes: (a) and (b) 387 nm thick coating layers by spraying for 8 s; (c) and (d) 72 nm thick coating layer by spraying for 4 s.

Supplementary note 23: To prepare the PVA/alumina composite membranes, an alumina membrane (53 μm thick) was carefully stuck on a PTFE board by scotch tape. A 0.4 wt. % PVA/P(AA-AMPS) (7:3 w/w) solution (PH=1), was sprayed perpendicularly to the surface of the alumina membrane at a distance of 15 cm. The spraying times were controlled at 4s or 8s, corresponding to the coating layer thicknesses of 72 ±15 nm and 387 ±32 nm, respectively, as shown in **Supplementary Figure 31**. After spray coating, the composite membranes were heated at 100 ℃ for 15 mins for crosslink. At last, the membranes were soaked in water at room temperature for 48 h to remove any soluble component and residual acid (catalyst, H_2SO_4) before further tests.

PV desalination performance of the PVA/alumina composite membranes

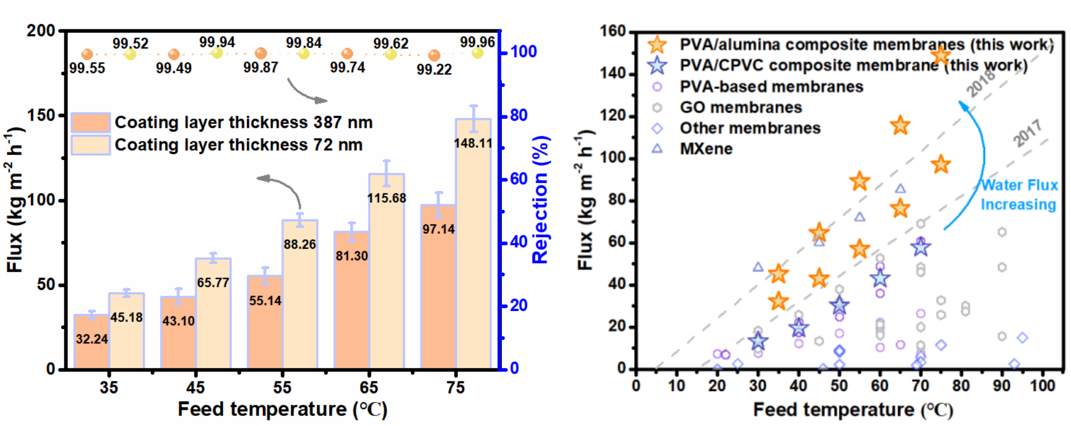

(a) (b)

Supplementary Figure 32: (a) the desalination performance of the PVA/alumina composite membranes at different temperatures for 3.5 wt. % NaCl feed solution. (b) Comparison of the PV desalination performance of PVA/alumina composite membranes to literature data (the detailed information is listed in **Supplementary Table 9**, lines with double cap are error bars, mean ±s.d. for n = 5)

Supplementary note 24: PV desalination performances of the PVA/alumina composite membranes were evaluated in terms of water flux (kg m^{-2} h^{-1}) and salt rejection (%) using a 3.5 wt. % NaCl feed of different temperatures (35 ℃ to 75 ℃) and the results were shown in **Supplementary Figure 32a**. NaCl rejections of the two composite membranes were all higher than 99.22 %, indicating that the thin PVA/P(AA-AMPS) coating layers were defect-free. As the PVA layer thicknesses reduced from 387 nm to 72 nm, water fluxes increased from 97.14 ±7.29 to 148.11 ±7.71 kg m^{-2} h^{-1} at 75 ℃. Apparently, the 5 to 30 times thinner PVA/P(AA-AMPS) layers to the PVA/CPVC membranes (2 μm) led to the 1.7 to 2.5 times higher water fluxes. Water fluxes of the PVA/alumina membranes already exceeded the MXene membrane (**Supplementary Figure 32b**).

Electrospinning of PAN nanofiber substrates

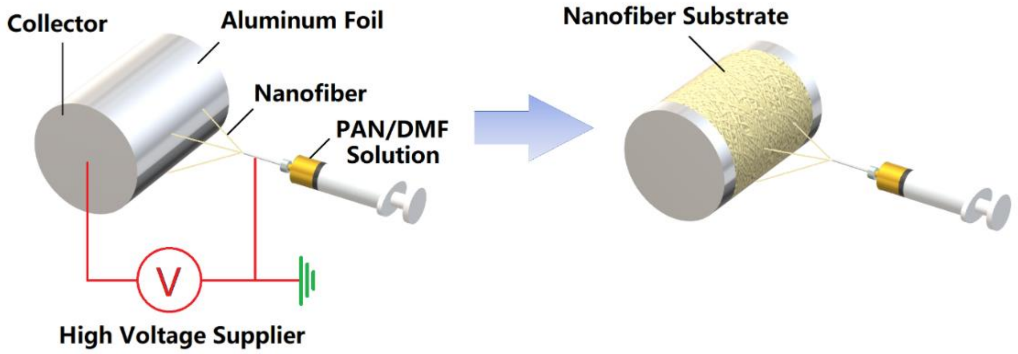

Supplementary Figure 33: Preparation of the PAN nanofiber mats

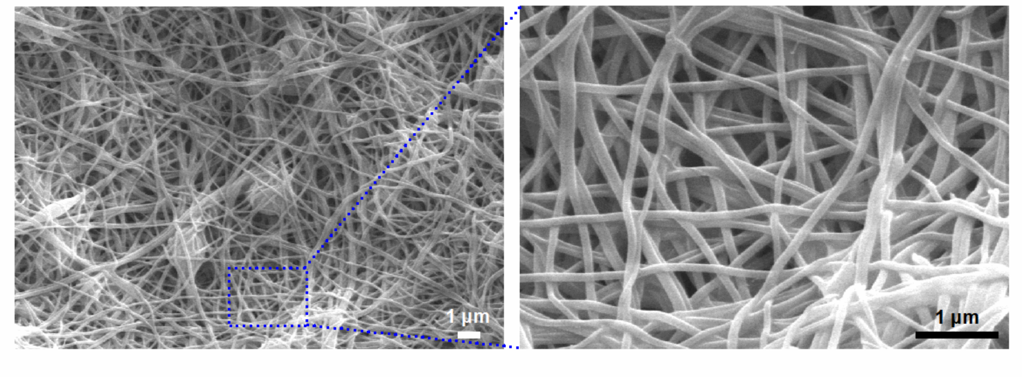

(a)

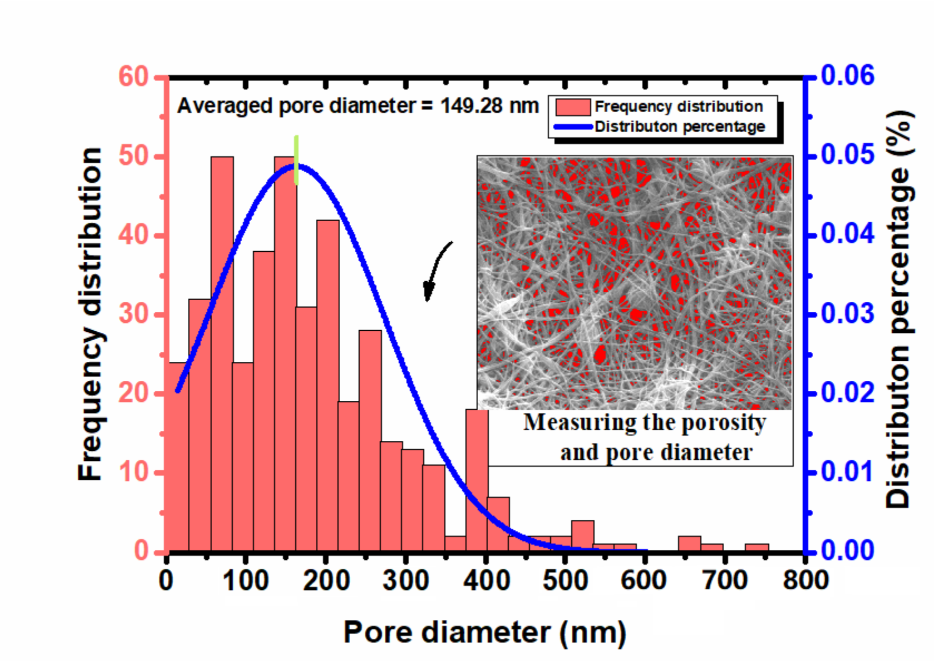

(b)

Supplementary Figure 34: (a) Surface FE-SEM images and (b) pore size distribution of the PAN nanofiber substrate.

Supplementary note 25: The PAN nanofiber was deposited on a glossy aluminum foil by

electrospinning and used as the porous substrate (as shown in **Supplementary Figure 33**). Specifically, 8 g PAN was dissolved in 92 g DMF and stirred at 50 ℃ for 24 h to obtain a homogeneous solution. Then an 8 mL PAN/DMF solution was filled in a syringe equipped with a 0.7 mm spinneret. The applied electric voltage and the solution feed rate was 24 kV and 10 μL min^{-1}. The spinneret had a translational oscillatory motion perpendicular to the collector rotation direction (oscillation distance was 30 cm) driven by a step motor. A rotating collector (diameter: 10 cm, width: 30 cm) covered by a glossy aluminum foil was used to collect the PAN nanofiber at a rotating speed of 180 rpm. The distance between the spinneret and the collector was 17 cm. After the 8 mL PAN/DMF solution was consumed, the PAN nanofiber substrate with an average thickness of 20.9 ± 3.7 μm was obtained. Surface morphology and pore size distribution of PAN nanofibers are shown in **Supplementary Figure 34**. The average pore size and surface porosity were 149.3 nm and 32.74 %, respectively.

Preparation of PVA/PAN nanofiber composite membranes

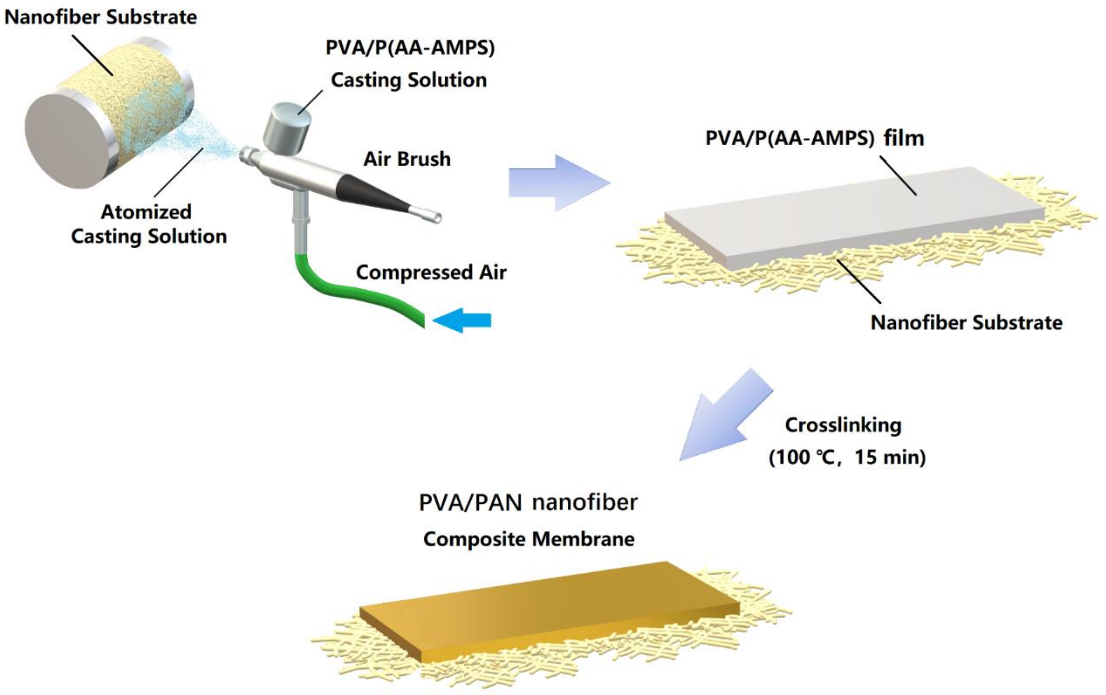

Supplementary Figure 35: Preparation protocol of the PVA/PAN nanofiber composite membrane

Supplementary note 26: Since the PAN nanofibers were thin (100-150 nm) and soft, conventional coating methods such as knife coating or drip coating would easily destroy the nanofibers. Therefore, the top layers were coated by air spraying using a similar method introduced in the **Supplementary Figure 29** except that the concentration of the coating solutions increased to 0.75 wt.% to increase the viscosity and prevent intrusion of the solution into the substrate. **Supplementary Table 4** lists the compositions of the coating solutions. The PAN nanofiber substrate was rotated at a speed of 180 rpm. The airbrush was in a translational oscillatory motion (speed: 6 cm s^{-1}) perpendicular to the collector rotation direction (oscillation distance was 30 cm) driven by a step motor (**Supplementary Figure 35**). The total spray times were varied from 10 s to 40 s to provide the uniformly coating layers with different thicknesses. After coating, the composite membranes were crosslinked at 100 ℃ for 15 min and soaked in water at room temperature for 48 h to remove soluble components.

Compression capacity of the water-swollen coating layers on PAN nanofibers mats

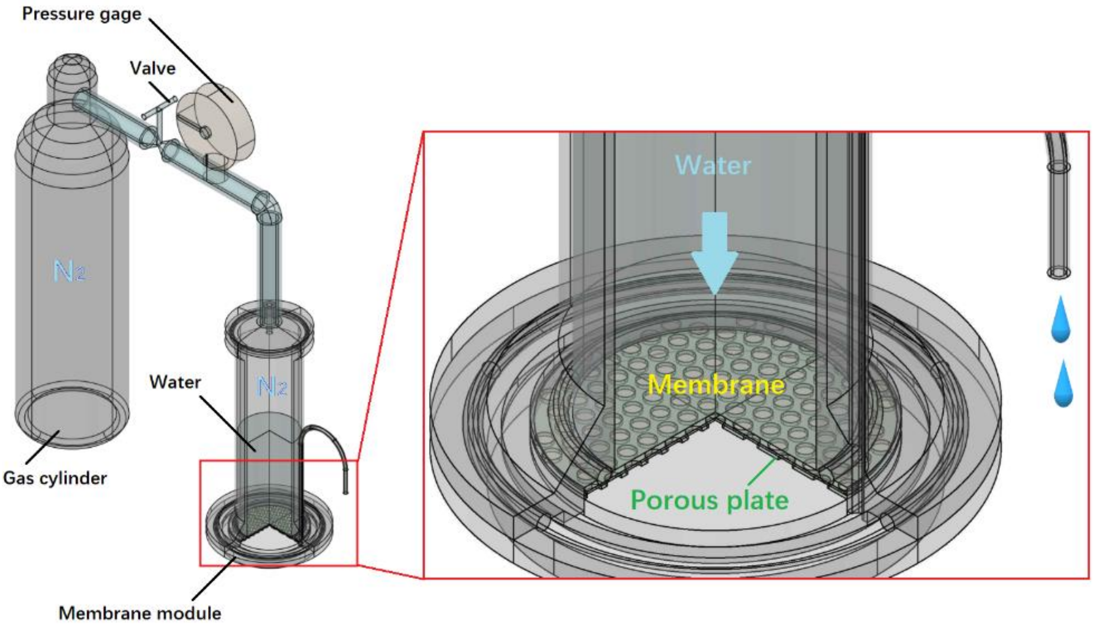

(c)

Supplementary Figure 36: The diagrammatic sketch of (c) ultrafiltration devices.

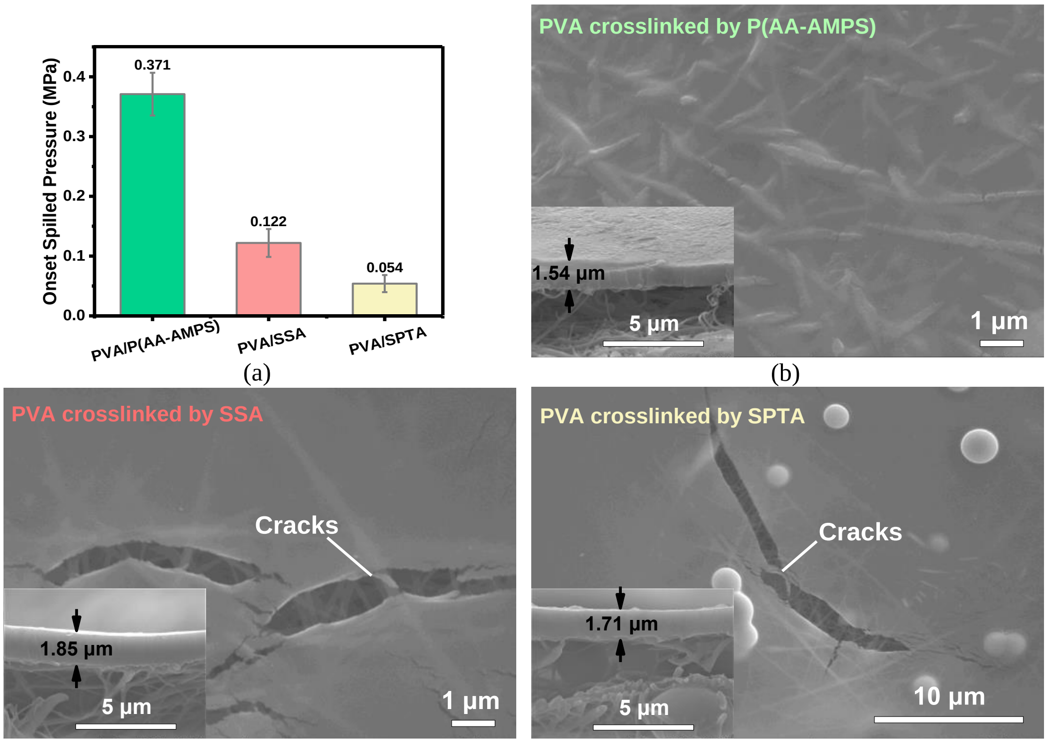

Supplementary Figure 37: (a) Onset spilled pressure of PVA/PAN nanofiber composite membranes, the coating layers were crosslinked at 100 ℃ for 15 min by SSA, SPTA and P(AA-AMPS), spray time was 20 s, compositions of PVA/crosslinker spraying solutions are listed in **Supplementary Table 4**. Surface topography of coating layers crosslinked by (b) P(AA-AMPS), (c) SSA and (d) SPTA under PV operational pressure (0.1 MPa), the insert SEM images are the thickness of PVA-based coating layer. (lines with double cap are error bars, mean ± s.d. for n = 3)

Supplementary note 27: A dead-end ultrafiltration device (HP4750, STERLITECH corporation, USA) was used to measuring the compression capability of the water-swollen coating layer on PAN nanofiber mats (**Supplementary Figure 36**). Composite membranes were placed on top of a porous metal plate. 5 cm high water was placed on top of the composite membrane and then the pressure of the membrane upstream was gradually increased by 0.005 MPa per 5 min until water passed to the permeate side. The onset spilled pressures were recorded to determine the compressive capacity of coating layers. The effective membrane area was 11.36 cm^2 and the tests were operated at ambient temperature.

To investigate the compression capacity, three PVA/PAN nanofiber composite membranes with PVA-based thin films crosslinked by SSA, SPTA and P(AA-AMPS) were firstly fabricated. Then these films were placed in a dead-end ultrafiltration device to measure the onset water spilled pressures (as depicted in **Supplementary Figure 36**). Higher onset spilled pressure indicated better compressive ability and stronger mechanical nature for coating layer. As can be seen from **Supplementary Figure 37a**, swollen coating layer crosslinked by P(AA-AMPS) had the best compression capacity (onset spilled pressure = 0.371 ±0.036 MPa), and without any cracks when the loading pressure reached up to 0.1 MPa (**Supplementary Figure 37b**). The lower onset spilled pressures for coating layer crosslinked by SSA and SPTA were 0.122 ±0.023 and 0.054 ±0.014 MPa, respectively. And, when the loading pressure reached up to PV desalination operational pressure (0.1 MPa), coating layers crosslinked by SSA and SPTA broken up and cracked (**Supplementary Figures 37c** and **d**). This means PVA-based thin films crosslinked by SSA and SPTA cannot cover PAN nanofiber substrate under PV operational pressure (0.1 MPa) without any deformation. Therefore, PVA-based film crosslinked by P(AA-AMPS) is the most suitable coating layer for fabricating PVA/PAN nanofiber composite membranes for its strengthened mechanical nature by crosslinking.

Effects of coating layer thicknesses and feed temperatures on PV desalination performance

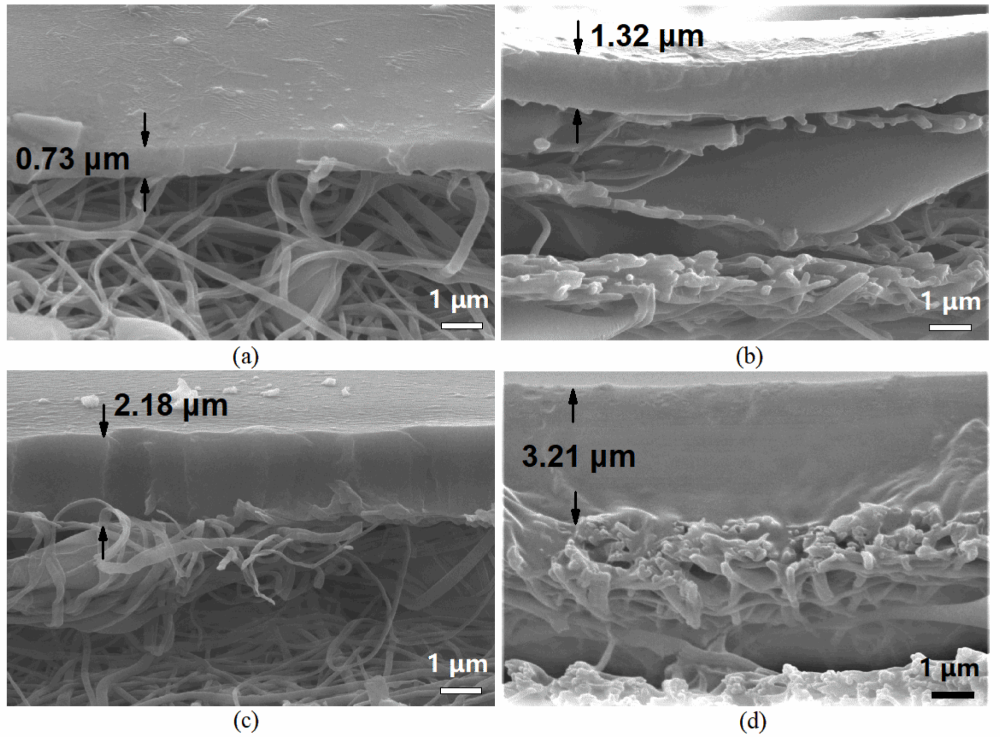

Supplementary Figure 38: Cross-sectional images of PVA/PAN nanofiber composite membrane and the thickness of PVA/P(AA-AMPS) coating layer with different total spray time: (a) 10 s, (b) 20 s, (c) 30 s, (d) 40s.

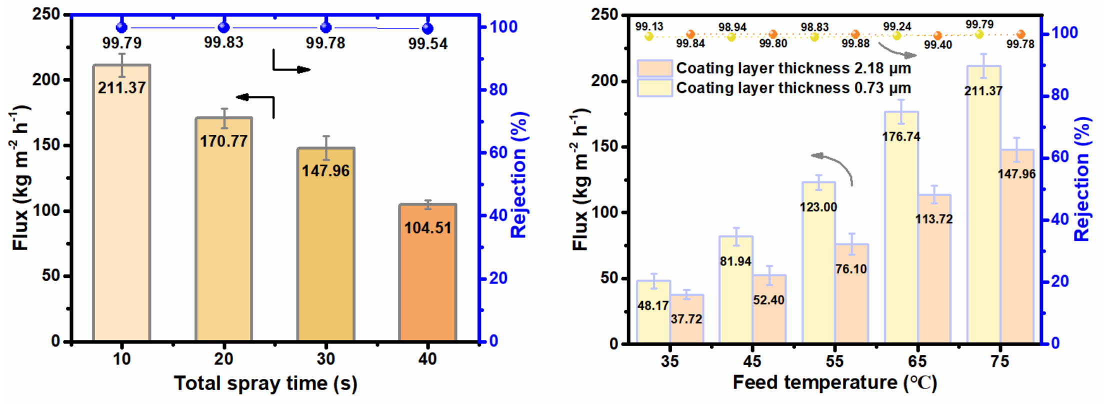

(a) (b)

Supplementary Figure 39: (a) PV desalination performance of the PVA/PAN nanofiber composite membranes with different coating layer thicknesses. (b) Effects of feed temperature on the PV desalination properties. 3.5 wt. % NaCl as feed solution. (lines with double cap are error bars, mean ±s.d. for n = 5)

Supplementary note 28: As shown in **Supplementary Figure 38**, thicknesses of the PVA layers increased from 0.73±0.03 to 3.21± 0.23 μm, as the spray coating time increased from 10 to 40 s. While water fluxes of the PVA/ nanofiber composite membranes decreased from 211.37 ±11.27 to 104.51 ± 3.28 kg m^{-2} h^{-1} kg m^{-2} h^{-1} (**Supplementary Figure 39a**) at 75 ℃ with the salt rejections higher than

99.79 %, indicated the coating layers were defect-free. Note that, at the similar PVA/P(AA-AMPS) layer thickness of 2 μm, water flux of the PVA/nanofiber membrane (141.18 ± 9.11 kg m^{-2} h^{-1}) was 2.4 times higher than that of the PVA/CPVC membrane (57.91 ± 1.83 kg m^{-2} h^{-1}). Obviously, the dramatically increment in water flux was resulted from the lower resistance of the PAN nanofiber substrate [43,44]. **Supplementary Figure 39b** shows the effect of feed temperature on water flux. The trend was similar to the PVA/CPVC and PVA/alumina composite membranes and could be explained by the higher feed side vapor pressure (stronger driving force), faster water diffusivity and accelerated facilitate transport behavior.

Effect of substrates resistance on PV desalination performance

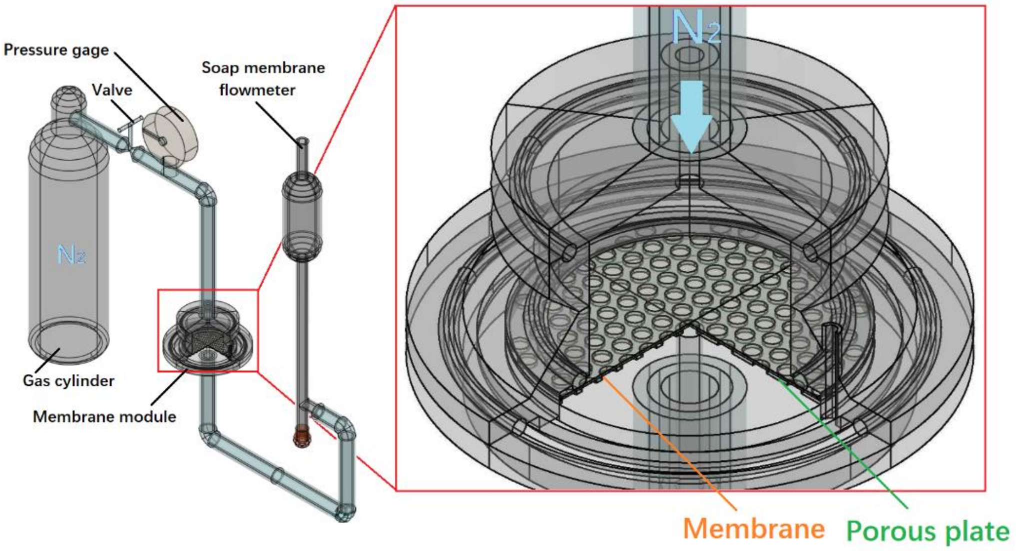

Supplementary Figure 40: The diagrammatic sketch of (b) gas permeation cell,

Supplementary note 29: To determine the resistance of the porous substrates to water vapor permeation, a gas permeation cell (CAT.NO.XX4404700, MIL.LIPORE CORP., Japan, **Supplementary Figure 40**) was used to correlate the relation between pressure build-up and gas flux. N_2 was used as feed gas because it was easier to be operated than water vapor [33]. A detailed experimental procedure could be found in **Supplementary Reference** [45]. N_2 flux (Q: L m^{-2} h^{-1}) was calculated using **Supplementary Equation 15**:

$$Q = \frac{V}{S_2 \cdot t_2} \quad (15)$$

where V was the volume of the permeated gas (L); S_2 was the effective membrane area (m^2), and t_2 was the permeation time (h). The resistance could be estimated by calculating the slope of Q versus the trans-membrane pressures.

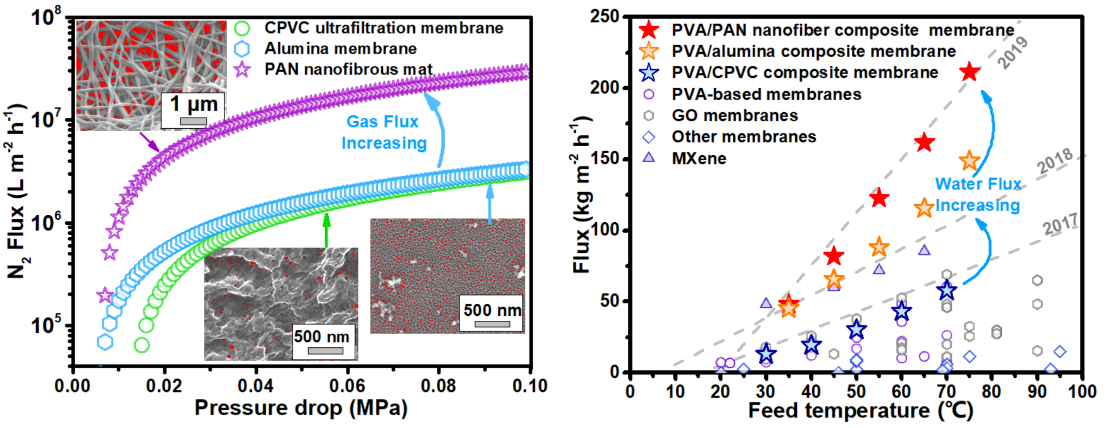

(a) (b)

Supplementary Figure 41: (a) Relationships between gas fluxes and pressure drops of the porous substrates used in this work; and (b) comparison of desalination performances of the PV membranes prepared by different groups using NaCl solutions as feed.

Supplementary note 30: To compare the substrates' resistances, gas permeance of the CPVC, alumina membrane, and PAN nanofiber substrates were measured using the method introduced in **Supplementary Reference**[33]. The Poiseuille equation was used to correlate pressure drop through a membrane with gas flux as given below [46]:

$$\Delta P = \frac{8\psi\eta RTh_mJ_p}{r^2\epsilon M_{gas}P} \quad \textbf{(16)}$$

ΔP: pressure drop cross porous substrate (Pa),
Ψ: pore tortuosity along thickness,
η: gas viscosity (Pa s),
R: gas constant (J mol^{-1} K^{-1}),
T: temperature (K),
h_m: porous substrate thickness (m),
J_p: mass flux through membrane (kg m^{-2} s^{-1}),
r: membrane pore radius (m),
ϵ: membrane porosity,
M_{gas}: gas molecular weight (kg mol^{-1}),
P: average gas pressure within membrane (Pa).

According to **Supplementary Equation 16**, the pressure drop was more sensitive to pore size than other parameters. An increase in pore radius would lead to a dramatically decrease in pressure drop. **Supplementary Figure 41a** shows that PAN nanofiber substrate has the lowest resistance that agrees well with its largest surface pore size (149 nm) among three substrates. For CPVC and alumina substrates, although there was big difference in their surface porosities (7.19 % vs. 47.1 %), their resistances to gas permeate were very similar because of the similar pore sizes (11.33 nm and 8.49 nm).

In **Supplementary Figure 41b**, water fluxes of the composite membranes fabricated in this work were compared with other high-performance PV desalination membranes. The fluxes of PVA/CPVC composite membranes were comparable to the reported data. However, the water fluxes of PVA/alumina and PVA/nanofiber composite membranes are 1.7 and 2.5 times higher than the highest reported water flux of MXene/PAN composite membranes at 65 ℃. The dramatically improved water fluxes could be attributed to three factors: first, the P(AA-AMPS) crosslinked PVA had water facilitate transport groups (sulfonic acids) that accelerated water permeation (the PVA/alumina composite membrane had similar PVA layer thicknesss of 72 nm to the MXene/PAN membrane (60 nm) but showed 1.7 times higher water flux); second, the PAN nanofiber substrate had much lower resistance so that the PVA/nanofiber composite membrane had 2.5 times higher water flux than MXene/PAN membrane although the PVA layer thickness (0.7 μm) was 11 times thicker; third, the P(AA-AMPS) crosslinker provided good mechanical strength of the crosslinked PVA thin film so that the PVA/nanofiber membranes maintained the coating layer integrity under vacuum.

Comparison the desalination properties between PV desalination membranes and MD

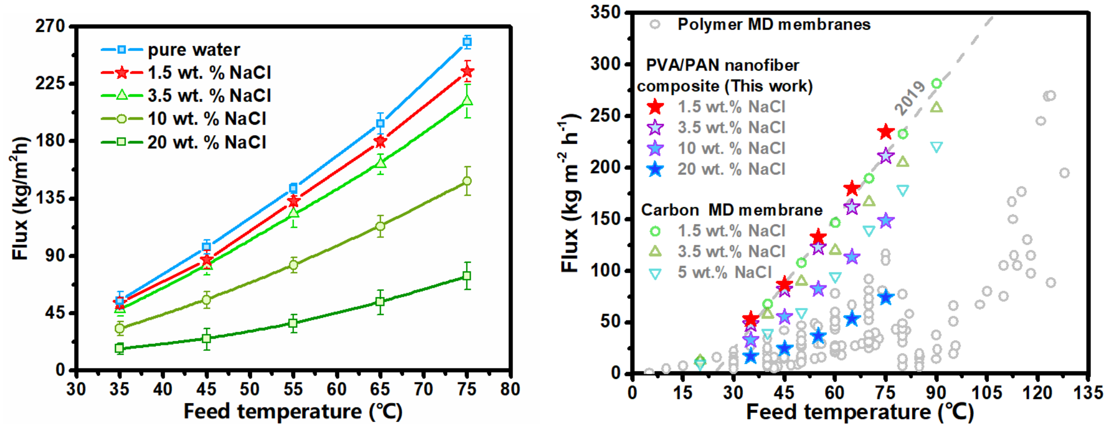

(a) (b)

Supplementary Figure 42: (a) water fluxes of the PVA/PAN nanofiber composite membranes at different salt concentrations and temperatures; and (b) Comparing the water fluxes of PV desalination composite membranes to MD data from **Supplementary Reference** [47-57]. (lines with double cap are error bars, mean ±s.d. for n = 5)

Supplementary note 31: To our best knowledge, only the inorganic MD membrane synthesized by nano-porous carbon showed higher water flux of 282.7 kg m^{-2} h^{-1} when desalinating a 1.5 wt. % NaCl solution at 90 ℃ [57]. However, MD membranes suffered from serious fouling problems such as rapid scaling and pore blocking especially when the feed solution had high salt concentration and organic foulant [58]. To investigate the desalination properties of our PV membranes under high salt concentrations, the water fluxes and salt rejections at the feed temperatures of 35 -75 ℃ and NaCl concentrations of 3.5 - 20 wt. % were measured. As shown in **Supplementary Figure 42a**, although the water fluxes decreased with the NaCl concentration, a high-water flux of 74.09 ±10.68 kg m^{-2} h^{-1} was still obtained when treated a 20 wt. % NaCl solution at 75 ℃. Desalination performance of the PV membranes were compared with the MD membranes as shown in **Supplementary Figure 42b**. At a temperature range of 30-75 ℃, the PVA/nanofiber composite membranes had higher water fluxes than all MD membranes.

Long-term and anti-fouling performance of PVA/PAN nanofiber composite membranes

Supplementary Table 8. The characteristics of foulants in this work.

Name	Type	Chemical structure
Sodium alginate (SA)	Bio foulant	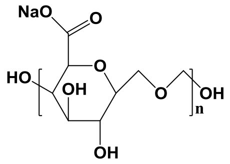
Tween 20	Nonionic surfactant	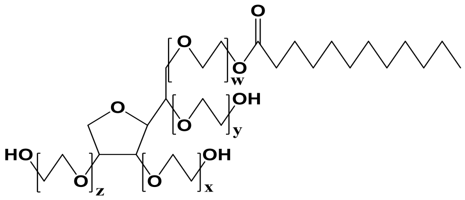
Sodium dodecyl benzene sulfonate (SDBS)	Anionic surfactant	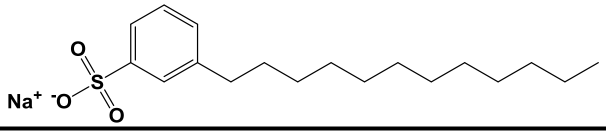

Supplementary note 32: Sodium alginate (SA) is a kind of natural organic matter (NOM) and is considered to be the main foulant in membrane water treatment[59]. Sodium dodecyl benzene sulfonate (SDBS) and Tween 20 are not NOM, they are surfactants and chemical cleaners for proteins[60]. Proteins widely exist in raw sea water, and membranes fouled by proteins can be cleaned by surfactants such as SDBS or Tween 20 [61]. On the other hand, anionic/nonionic surfactants are always used to test the anti-wettability of hydrophobicity MD membranes[62]. So, the anti-surfactants fouling properties should be considered as an important membrane performance for membrane processes without strict pretreatments. Therefore, we choose SDBS and Tween 20 act as model foulants to test the anti-fouling performance of PVA/P(AA-AMPS) membrane.

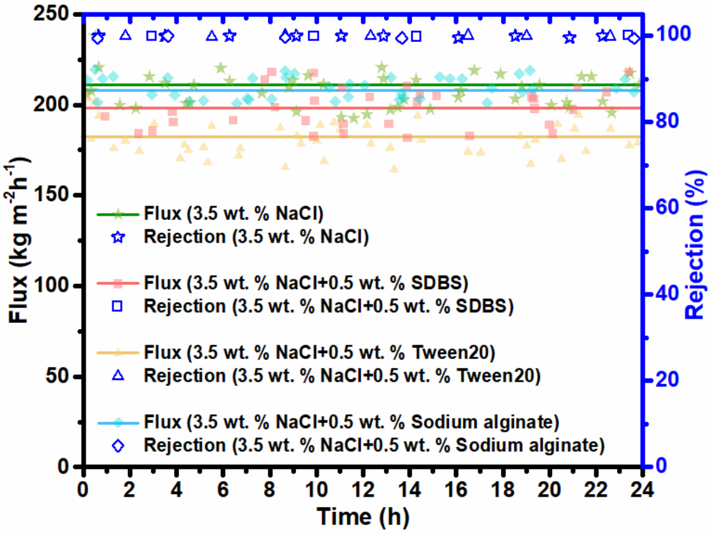

Supplementary Figure 43: the long-term desalination properties of the PVA/nanofiber composite membrane when treating a 3.5 wt. % NaCl solution and a 3.5 wt. % NaCl solution with 0.5 wt. % Tween 20 or sodium dodecyl benzene sulfonate (SDBS) or sodium alginate acting as an organic foulant in feed solution.

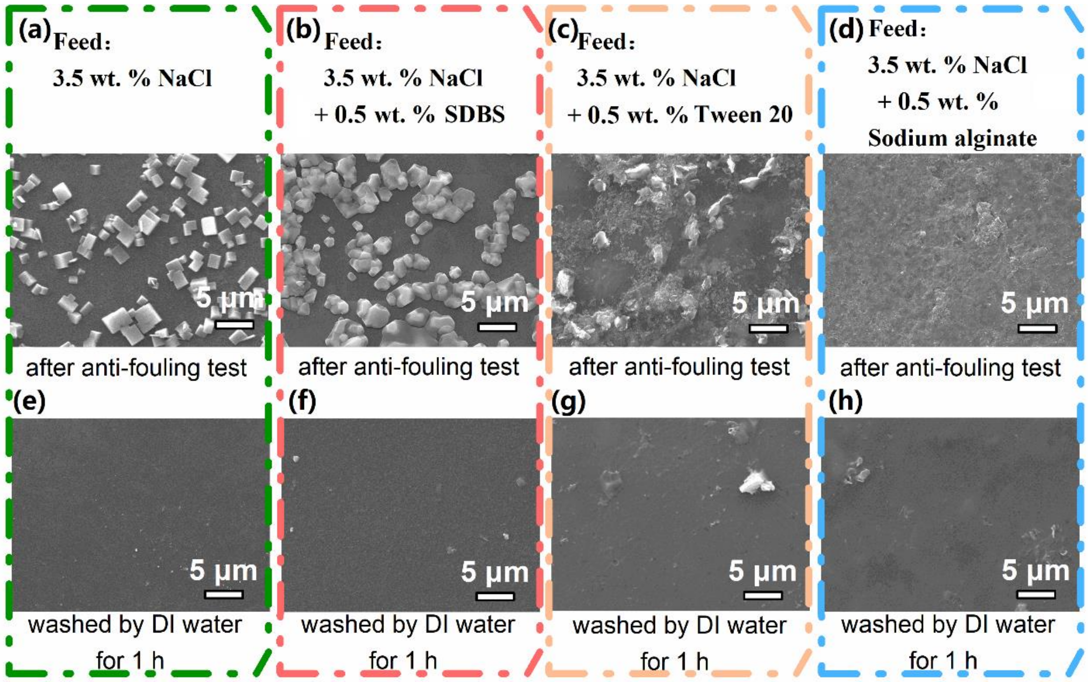

Supplementary Figure 44: Surface SEM images of PVA/P(AA-AMPS) coating layers after antifouling tests (a-d) and being washed by DI water for 1 h (e-h).

Supplementary note 33: Supplementary Figure 43 shows the long-term desalination performance and anti-fouling property of the composite membranes. At 75 ℃, approximately 1.67 liters of freshwater was collected from a 3.5 wt. % NaCl solution after 24 h using a PVA/nanofiber membrane with an effective area of 3.28×10^{-4} m^2, which gave a water flux of 211.37 ± 11.27 kg m^{-2} h^{-1}. Whilst, the NaCl rejection was maintained above 99.53 %. When the 3.5 wt. % NaCl feed solution contained 0.5 wt. % SDBS or Tween 20 or sodium alginate as a pollutant[62], a higher water flux than 181.67 ± 12.44 kg m^{-2} h^{-1} with salt rejection above 99.65% were maintained in the 24 h experimental duration. This result indicated good anti-fouling property of the dense PVA layer that prevented the pore-blocking of the substrates by the organic fouling and salt scaling. The 15 % decrease in water flux from 211.37 ± 11.27 kg m^{-2} h^{-1} to 181.67 ± 12.44 kg m^{-2} h^{-1} could be attributed to the possible cake layer formed on the PVA layer surface, as shown in **Supplementary Figures 44 a-d**. Although the cake layers formed on coating layer surface is inevitable when feed solutions containing hydrophilic polymers, they can be easily washed off by DI water for 1 h, as shown in **Supplementary Figures 44 e-h**.

PV desalination performance from supplementary references

Supplementary Table 9. The desalination data of the PV membranes in literatures

Membrane	NaCl (ppm)	Feed temperature (℃)	Thickness (μm)	Flux (kg/(m2 h))	Rejection (%)	Reference
Cellulose triacetate	100,000	50	10	2.3	99	63
Cellulose acetate	40,000-140,000	70	20-25	5.97-3.45	99.7	64
Polyester	35,000	20	750	0.0071	99.84	65
poly(vinyl alcohol)/polyacrylonitrile	5000	20	0.62	9.04	99.5	66
poly(vinyl alcohol)/MA	30,000	70	0.1	7.4	99.9	67
Clinoptilolite	100	93		2.5	95.8	68
Clinoptilolite-phosphate	1400	95		15	95	69
Carbon template silica	40,000	25	0.21	2.6	99.9	70
Polyether amide	35,000	46-82	40	0.2	99.9	71
NaA zeolite membrane	35,000	69		1.9	99.9	72
graphene oxide /polyacrylonitrile	35,000	70		46.1	99.8	73
graphene oxide /polyacrylonitrile	35,000	90		65.1	99.8	73
Zeolite (ZSM-5)	3,000-10,000	75	3.3	11.5	99	74
poly(vinyl alcohol) / MA / inorganic silica	2,000	22	5	6.93	99.5	75
poly(vinyl alcohol) / MA / inorganic silica	0-50,000	65	20	11.7	99.9	76
graphene oxide/chitosan	50,000	81	10-13	30.0	99.99	77
graphene oxide/chitosan	100,000	81	10-13	27.6	99.99	77
graphene oxide/chitosan	50,000	75	10-13	25.8	99.99	77
graphene oxide/chitosan	35,000	60	10-13	17.7	99.99	77
graphene oxide/chitosan	100,000	60	10-13	16.2	99.99	77
PVA/silica	2,000	60	0.22±0.03	20.4	99.9	78
PVA/silica	30,000	60	0.22±0.03	10.4	99.9	78
ZIF-7/a-Al2O3 disk	35,000	50	20	9.2	99.5	79
ZIF-8/a-Al2O3 disk	35,000	50	20	8.1	99.8	79
ZIF-90/a-Al2O3 disk	35,000	50	20	8.9	99.7	79
MXene/PAN	35,000	30	0.06	48.2	99.5	40
MXene/PAN	35,000	65	0.06	85.4	99.5	40
graphene oxide/ceramic	35,000	90	0.4	48.4	99.7	80
S-PVA/PSF	35,000	70	1.12	60.8	99.8	33
poly(vinyl alcohol)/polyacrylonitrile	35,000	30	-	14.3	99.8	39
S-PVA/PAN	35,000	70	0.8	46.3	99.5	39
poly(vinyl alcohol)/polyacrylonitrile	35,000	30	0.8	14.1	99.7	39
graphene oxide /a-Al2O3	35,000	90	1.6	20.1	99.9	81
graphene oxide /a-Al2O3	35,000	90	1.6	10.7	99.8	81
graphene oxide /a-Al2O3	35,000	90	1.6	11.4	99.9	81
graphene oxide/polyimide	35,000	90	-	15.6	99.8	82
graphene oxide /PAN	35,000	70	0.08	69.1	99.9	43
PVA/SSA/PVDF	35,000	70	4.9	27.9	99.8	83
PVA/Mil-53(Al)/PVDF	100,000	80	-	18.3	99.9	58

Program code of molecular dynamics simulation

```
#!perl
use strict;
use Getopt::Long;
use MaterialsScript qw(:all);

use strict;
use MaterialsScript qw(:all);
#open the multiframe trajectory structure file or die
my $doc = $Documents{"./***.xtd"};
if (!$doc) {die "no document";}
my $trajectory = $doc->Trajectory;
if ($trajectory->NumFrames>1) {
	print "Found ".$trajectory->NumFrames." frames in the trajectory\n";
	# Open new xmol trajectory file
	my $xmolFile=Documents->New("trj.txt");

	#get atoms in the structure
#	my $atoms = $doc->Atoms;
	my $atoms = $doc->DisplayRange->Atoms;
	my $Natoms=@$atoms;
	# loops over the frames
	my $framebegin=1;
	my $frameend=$trajectory->NumFrames;
#	my $frameend=1;
	for (my $frame=$framebegin; $frame<=$frameend; ++$frame){
		$trajectory->CurrentFrame = $frame;
		#write header xyz
		$xmolFile->Append(sprintf "%i \n", $Natoms);
		$xmolFile->Append(sprintf "%s %i \n", "Frame",$frame);
		foreach my $atom (@$atoms) {
			# write atom symbol and x-y-z- coordinates
			$xmolFile->Append(sprintf "%s %f    %f    %f \n",$atom->ElementSymbol, $atom->X,
$atom->Y,
$atom->Z);
		}
	}
	#close trajectory file
	$xmolFile->Close;
}
else {
     print "The " . $doc->Name . " is not a multiframe trajectory file \n";
}
```

Supplementary References

1 Uhnáková, A., Pokluda, J., Machová, A. & Hora, P. 3D atomistic simulation of fatigue behavior of a ductile crack in bcc iron loaded in mode II. *Computational Materials Science* **61**, 12-19, doi:10.1016/j.commatsci.2012.03.045 (2012).

2 Dong, X., Liu, Q., Cui, L., Yu, Y. & Zhang, M. Molecular simulation and experimental study on propylene dehumidification through a PVA–PAA blend membrane. *J. Mater. Chem. A* **2**, 16687-16696, doi:10.1039/c4ta03687e (2014).

3 Li, D., Panchal, K., Mafi, R. & Xi, L. An Atomistic Evaluation of the Compatibility and Plasticization Efficacy of Phthalates in Poly(vinyl chloride). *Macromolecules* **51**, 6997-7012, doi:10.1021/acs.macromol.8b00756 (2018).

4 Noorjahan, A. & Choi, P. Thermodynamic properties of poly(vinyl alcohol) with different tacticities estimated from molecular dynamics simulation. *Polymer* **54**, 4212-4219, doi:10.1016/j.polymer.2013.05.073 (2013).

5 Sun, H. Compass an ab initio force-field optimized for condensed-phase applications overview with details on alkane and benzene compounds. *J. Phys. Chem. B* **102**, 7338-7364 (1998).

6 Frenkel, D., Smit, B. & Ratner, M. A. Understanding Molecular Simulation: From Algorithms to Applications. *Physics Today* **50**, 66-66, doi:10.1063/1.881812 (1997).

7 MEILLER, M. F. A scaled conjugate gradient algorithm for fast supervised learning. *Neural Networks* **6**, 525-533 (1993).

8 Karlsson, G. E., Johansson, T. S., Gedde, U. W. & Hedenqvist, M. S. Physical properties of dense amorphous poly(vinyl alcohol) as revealed by molecular dynamics simulation. *Journal of Macromolecular Science, Part B* **41**, 185-206, doi:10.1081/mb-120003080 (2006).

9 Shinoda, W., Shiga, M. & Mikami, M. Rapid estimation of elastic constants by molecular dynamics simulation under constant stress. *Physical Review B* **69**, doi:10.1103/PhysRevB.69.134103 (2004).

10 Mavrantzas, V. G., Boone, T. D., Zervopoulou, E. & Theodorou, D. N. End-bridging Monte Carlo a fast algorithm for atomistic simulation of condensed phases of long polymer chains. *Macromolecules* **32**, 5072-5096 (1999).

11 Xue, Y. l., Lau, C. H., Cao, B. & Li, P. Elucidating the impact of polymer crosslinking and fixed carrier on enhanced water transport during desalination using pervaporation membranes. *J. Membr. Sci.* **575**, 135-146, doi:10.1016/j.memsci.2019.01.012 (2019).

12 Belmares, M. *et al.* Hildebrand and Hansen solubility parameters from molecular dynamics with applications to electronic nose polymer sensors. *J Comput Chem* **25**, 1814-1826, doi:10.1002/jcc.20098 (2004).

13 Karan, S., Jiang, Z. & Livingston, A. G. Sub–10 nm polyamide nanofilms with ultrafast solvent transport for molecular separation. *Science* **348**, 1347-1351 (2015).

14 JE, M. *Polymer Data Handbook*. (Oxford University Press, 1999).

15 Zhang, Q. G., Liu, Q. L., Chen, Y., Wu, J. Y. & Zhu, A. M. Microstructure dependent diffusion of water–ethanol in swollen poly(vinyl alcohol): A molecular dynamics simulation study. *Chem. Eng. Sci.* **64**, 334-340, doi:10.1016/j.ces.2008.10.028 (2009).

16 Florio, G. M., Zwier, T. S., Myshakin, E. M., Jordan, K. D. & Sibert, E. L. Theoretical modeling of the OH stretch infrared spectrum of carboxylic acid dimers based on first-principles anharmonic couplings. *The Journal of Chemical Physics* **118**, 1735-1746,

doi:10.1063/1.1530573 (2003).

17 Boek, E. S. & Briels, W. J. Molecular dynamics simulations of aqueous urea solutions: Study of dimer stability and solution structure, and calculation of the total nitrogen radial distribution function GN(r). *The Journal of Chemical Physics* **98**, 1422-1427, doi:10.1063/1.464306 (1993).

18 HALGREN, T. A. Merck Molecular Force Field. II. MMFF94 van der Waals and Electrostatic Parameters for Intermolecular Interactions. *Journal of Computational Chemistry* **17**, 520-552 (1996).

19 Takahata, Y. & Chong, D. P. Estimation of Hammett sigma constants of substituted benzenes through accurate density-functional calculation of core-electron binding energy shifts. *International Journal of Quantum Chemistry* **103**, 509-515, doi:10.1002/qua.20533 (2005).

20 G. B. Tolstorozhev *et al.* Infrared Spectroscopy of Hydrogen Bonds in Benzoic Acid Derivatives. *J. Appl. Spectrosc+* **81**, 109-117 (2014).

21 Meganathan, C., Sebastian, S., Kurt, M., Lee, K. W. & Sundaraganesan, N. Molecular structure, spectroscopic (FTIR, FTIR gas phase, FT-Raman) first-order hyperpolarizability and HOMO-LUMO analysis of 4-methoxy-2-methyl benzoic acid. *Journal of Raman Spectroscopy* **41**, 1369-1378, doi:10.1002/jrs.2562 (2010).

22 Bakker, J. M., Mac Aleese, L., von Helden, G. & Meijer, G. The infrared absorption spectrum of the gas phase neutral benzoic acid monomer and dimer. *The Journal of Chemical Physics* **119**, 11180-11185, doi:10.1063/1.1622657 (2003).

23 Halupka, M. & Sander, W. A simple method for the matrix isolation of monomeric and dimeric carboxylic acids. *Spectrochimica Acta Part A* **54**, 495-500 (1998).

24 James, J., Thomas, G. V. & Thomas, S. in *Transport Properties of Polymeric Membranes* (eds Sabu Thomas, Runcy Wilson, Anil Kumar S, & Soney C. George) 159-173 (Elsevier, 2018).

25 Flory, P. J. & Rehner, J. Statistical Mechanics of Cross‐Linked Polymer Networks II. Swelling. *The Journal of Chemical Physics* **11**, 521-526, doi:10.1063/1.1723792 (1943).

26 A.R.R. Menon, A.I. Aigbodion, C.K.S. Pillai, N.M. Mathew & Bhagawan, S. S. Processability characteristics and physico-mechanical properties of natural rubber modified with cashewnut shell liquid and cashewnut shell liquid–formaldehyde resin. *European Polymer Journal* **38**, 163-168 (2002).

27 Deng, L. *et al.* Oxidative crosslinking of copolyimides at sub-Tg temperatures to enhance resistance against CO2-induced plasticization. *Journal of Membrane Science* **583**, 40-48, doi:10.1016/j.memsci.2019.04.002 (2019).

28 Garg, P., Singh, R. P. & Choudhary, V. Selective polydimethylsiloxane/polyimide blended IPN pervaporation membrane for methanol/toluene azeotrope separation. *Separation and Purification Technology* **76**, 407-418, doi:10.1016/j.seppur.2010.11.012 (2011).

29 Lin, S., Yuan, C., Ke, A. & Quan, Z. Electrical response characterization of PVA–P(AA/AMPS) IPN hydrogels in aqueous Na2SO4 solution. *Sensors and Actuators B: Chemical* **134**, 281-286, doi:10.1016/j.snb.2008.04.045 (2008).

30 Materials., A. S. f. T. a. (2008).

31 Liu, Y., Lotero, E. & Goodwinjr, J. A comparison of the esterification of acetic acid with methanol using heterogeneous versus homogeneous acid catalysis. *Journal of Catalysis* **242**, 278-286, doi:10.1016/j.jcat.2006.05.026 (2006).

32 Cadogan, D. F. & Howick, C. J. in *Ullmann's Encyclopedia of Industrial Chemistry* (2000).

33 Li, Q., Cao, B. & Li, P. Fabrication of High Performance Pervaporation Desalination Composite Membranes by Optimizing the Support Layer Structures. *Industrial & Engineering Chemistry Research* **57**, 11178-11185, doi:10.1021/acs.iecr.8b02505 (2018).

34 Chung, J. Y., Chastek, T. Q., Fasolka, M. J., Ro, H. W. & Stafford, C. M. Quantifying Residual Stress in Nanoscale Thin Polymer Films via Surface Wrinkling. *ACS Nano* **3(4)**, 844-852 (2009).

35 Chung, J. Y., Lee, J. H., Beers, K. L. & Stafford, C. M. Stiffness, strength, and ductility of nanoscale thin films and membranes: a combined wrinkling-cracking methodology. *Nano Lett* **11**, 3361-3365, doi:10.1021/nl201764b (2011).

36 Urayama, K., Takigawa, T. & Masuda, T. Poisson's Ratio of Poly(viny1 alcohol) Gels. *Macromolecules* **26**, 3092-3096 (1993).

37 Tsai, C.-E., Lin, C.-W. & Hwang, B.-J. A novel crosslinking strategy for preparing poly(vinyl alcohol)-based proton-conducting membranes with high sulfonation. *J. Power. Sources.* **195**, 2166-2173, doi:10.1016/j.jpowsour.2009.10.055 (2010).

38 Meng, J., Li, P. & Cao, B. High-Flux Direct-Contact Pervaporation Membranes for Desalination. *ACS Appl Mater Interfaces* **11**, 28461-28468, doi:10.1021/acsami.9b08078 (2019).

39 Liang, B., Li, Q., Cao, B. & Li, P. Water permeance, permeability and desalination properties of the sulfonic acid functionalized composite pervaporation membranes. *Desalination* **433**, 132-140, doi:10.1016/j.desal.2018.01.028 (2018).

40 Liu, G. *et al.* Ultrathin two-dimensional MXene membrane for pervaporation desalination. *Journal of Membrane Science* **548**, 548-558, doi:10.1016/j.memsci.2017.11.065 (2018).

41 Choi, B. G., Hong, J., Hong, W. H., Hammond, P. T. & Park, H. Facilitated ion transport in all-solid-state flexible supercapacitors. *ACS Nano* **5**, 7205-7213, doi:10.1021/nn202020w (2011).

42 Jimenez-Solomon, M. F., Song, Q., Jelfs, K. E., Munoz-Ibanez, M. & Livingston, A. G. Polymer nanofilms with enhanced microporosity by interfacial polymerization. *Nat Mater* **15**, 760-767, doi:10.1038/nmat4638 (2016).

43 Cheng, C. *et al.* Robust construction of a graphene oxide barrier layer on a nanofibrous substrate assisted by the flexible poly(vinylalcohol) for efficient pervaporation desalination. *J. Mater. Chem. A* **5**, 3558-3568, doi:10.1039/c6ta09443k (2017).

44 Wijmans, J. G. & Hao, P. Influence of the porous support on diffusion in composite membranes. *Journal of Membrane Science* **494**, 78-85, doi:10.1016/j.memsci.2015.07.047 (2015).

45 Liu, X., Cao, B. & Li, P. Effects of Spinning Temperature on the Morphology and Performance of Poly(ether sulfone) Gas Separation Hollow Fiber Membranes. *Industrial & Engineering Chemistry Research* **57**, 329-338, doi:10.1021/acs.iecr.7b03990 (2017).

46 SCHOFIELD, R. W., FANE, A. G. & FELL, C. J. D. Gas and vapour transport through microporous membranes. I. Knudsen-Poiseuille transition. *Journal of Membrane Science*, 159-171 (1990).

47 Lawson, K. W. & Lloyd, D. R. Membrane distillation. II. Direct contact MD. *Journal of Membrane Science* **120**, 123-133 (1996).

48 Hsu, S. T., Cheng, K. T. & Chiou, J. S. Seawater desalination by direct contact membrane distillation. *Desalination* **143**, 279-287 (2002).

49 Yuna, Y., Ma, R., Zhang, W., Fane, A. G. & Li, J. Direct contact membrane distillation mechanism for high concentration NaCl solutions. *Desalination* **188**, 251-262,

doi:10.1016/j.desal.2005.04.123 (2006).

50 SCHOFIELD, R. W., FANE, A. G., FELL, C. J. D. & MACOUN, R. Factors affecting flux in membrane distillation. *Desalination* **77**, 279-294 (1990).

51 Singh, D. & Sirkar, K. K. Desalination of brine and produced water by direct contact membrane distillation at high temperatures and pressures. *Journal of Membrane Science* **389**, 380-388, doi:10.1016/j.memsci.2011.11.003 (2012).

52 Adnan, S., Hoang, M., Wang, H. & Xie, Z. Commercial PTFE membranes for membrane distillation application: Effect of microstructure and support material. *Desalination* **284**, 297-308, doi:10.1016/j.desal.2011.09.015 (2012).

53 Bhadra, M., Roy, S. & Mitra, S. Flux enhancement in direct contact membrane distillation by implementing carbon nanotube immobilized PTFE membrane. *Separation and Purification Technology* **161**, 136-143, doi:10.1016/j.seppur.2016.01.046 (2016).

54 Singh, D. & Sirkar, K. K. Performance of PVDF flat membranes and hollow fibers in desalination by direct contact membrane distillation at high temperatures. *Separation and Purification Technology* **187**, 264-273, doi:10.1016/j.seppur.2017.06.012 (2017).

55 Singh, D. & Sirkar, K. K. High temperature direct contact membrane distillation based desalination using PTFE hollow fibers. *Chemical Engineering Science* **116**, 824-833, doi:10.1016/j.ces.2014.05.042 (2014).

56 Li, K., Hou, D., Fu, C., Wang, K. & Wang, J. Fabrication of PVDF nanofibrous hydrophobic composite membranes reinforced with fabric substrates via electrospinning for membrane distillation desalination. *J Environ Sci (China)* **75**, 277-288, doi:10.1016/j.jes.2018.04.002 (2019).

57 Chen, W. *et al.* High-flux water desalination with interfacial salt sieving effect in nanoporous carbon composite membranes. *Nat Nanotechnol* **13**, 345-350, doi:10.1038/s41565-018-0067-5 (2018).

58 Liang, W. *et al.* Linking defects, hierarchical porosity generation and desalination performance in metal-organic frameworks. *Chem Sci* **9**, 3508-3516, doi:10.1039/c7sc05175a (2018).

59 Hashino, M. *et al.* Effect of membrane surface morphology on membrane fouling with sodium alginate. *Journal of Membrane Science* **366**, 258-265, doi:https://doi.org/10.1016/j.memsci.2010.10.014 (2011).

60 Aghajani, M., Rahimpour, A., Amani, H. & Taherzadeh, M. J. Rhamnolipid as new bio-agent for cleaning of ultrafiltration membrane fouled by whey. *Eng. Life Sci.* **18**, 272–280, doi:10.1002/elsc.201700070 (2018).

61 Lee, H., Hong, M., Han, S., Shim, J. & Moon, S. Analysis of fouling potential in the electrodialysis process in the presence of an anionic surfactant foulant. *Journal of Membrane Science* **325**, 719-726, doi:10.1016/j.memsci.2008.08.045 (2008).

62 Lin, P.-J., Yang, M.-C., Li, Y.-L. & Chen, J.-H. Prevention of surfactant wetting with agarose hydrogel layer for direct contact membrane distillation used in dyeing wastewater treatment. *Journal of Membrane Science* **475**, 511-520, doi:10.1016/j.memsci.2014.11.001 (2015).

63 Huth, E., Muthu, S., Ruff, L. & Brant, J. A. Feasibility assessment of pervaporation for desalinating high-salinity brines. *Journal of Water Reuse and Desalination* **4**, 109-124, doi:10.2166/wrd.2014.038 (2014).

64 Naim, M., Elewa, M., El-Shafei, A. & Moneer, A. Desalination of simulated seawater by purge-air pervaporation using an innovative fabricated membrane. *Water Sci Technol* **72**,

785-793, doi:10.2166/wst.2015.277 (2015).

65 Sule, M. *et al.* Salt rejection and water flux through a tubular pervaporative polymer membrane designed for irrigation applications. *Environ Technol* **34**, 1329-1339, doi:10.1080/09593330.2012.746736 (2013).

66 Liang, B., Pan, K., Li, L., Giannelis, E. P. & Cao, B. High performance hydrophilic pervaporation composite membranes for water desalination. *Desalination* **347**, 199-206, doi:10.1016/j.desal.2014.05.021 (2014).

67 Chaudhri, S. G., Rajai, B. H. & Singh, P. S. Preparation of ultra-thin poly(vinyl alcohol) membranes supported on polysulfone hollow fiber and their application for production of pure water from seawater. *Desalination* **367**, 272-284, doi:10.1016/j.desal.2015.04.016 (2015).

68 Swenson, P., Tanchuk, B., Gupta, A., An, W. & Kuznicki, S. M. Pervaporative desalination of water using natural zeolite membranes. *Desalination* **285**, 68-72, doi:10.1016/j.desal.2011.09.035 (2012).

69 An, W. *et al.* Natural zeolite clinoptilolite-phosphate composite Membranes for water desalination by pervaporation. *Journal of Membrane Science* **470**, 431-438, doi:10.1016/j.memsci.2014.07.054 (2014).

70 SINGH, P. S. *et al.* Cetyltrimethylammonium bromide-silica membrane for seawater desalination through pervaporation. *materials research bulletin* **38**, 1-8 (2015).

71 Zwijnenberg, H., Koops, G. & Wessling, M. Solar driven membrane pervaporation for desalination processes. *Journal of Membrane Science* **250**, 235-246, doi:10.1016/j.memsci.2004.10.029 (2005).

72 Cho, C. H., Oh, K. Y., Kim, S. K., Yeo, J. G. & Sharma, P. Pervaporative seawater desalination using NaA zeolite membrane: Mechanisms of high water flux and high salt rejection. *Journal of Membrane Science* **371**, 226-238, doi:10.1016/j.memsci.2011.01.049 (2011).

73 Liang, B. *et al.* High performance graphene oxide/polyacrylonitrile composite pervaporation membranes for desalination applications. *J. Mater. Chem. A* **3**, 5140-5147, doi:10.1039/c4ta06573e (2015).

74 Drobek, M. *et al.* Long term pervaporation desalination of tubular MFI zeolite membranes. *Journal of Membrane Science* **415-416**, 816-823, doi:10.1016/j.memsci.2012.05.074 (2012).

75 Xie, Z. *et al.* Sol–gel derived poly(vinyl alcohol)/maleic acid/silica hybrid membrane for desalination by pervaporation. *Journal of Membrane Science* **383**, 96-103, doi:10.1016/j.memsci.2011.08.036 (2011).

76 Xie, Z., Ng, D., Hoang, M., Duong, T. & Gray, S. Separation of aqueous salt solution by pervaporation through hybrid organic–inorganic membrane: Effect of operating conditions. *Desalination* **273**, 220-225, doi:10.1016/j.desal.2010.10.026 (2011).

77 Qian, X., Li, N., Wang, Q. & Ji, S. Chitosan/graphene oxide mixed matrix membrane with enhanced water permeability for high-salinity water desalination by pervaporation. *Desalination* **438**, 83-96, doi:10.1016/j.desal.2018.03.031 (2018).

78 Chaudhri, S. G., Chaudhari, J. C. & Singh, P. S. Fabrication of efficient pervaporation desalination membrane by reinforcement of poly(vinyl alcohol)-silica film on porous polysulfone hollow fiber. *Journal of Applied Polymer Science* **135**, doi:10.1002/app.45718 (2018).

79 Zhu, Y. *et al.* Synthesis and seawater desalination of molecular sieving zeolitic imidazolate

framework membranes. *Desalination* **385**, 75-82, doi:10.1016/j.desal.2016.02.005 (2016).

80 Xu, K., Feng, B., Zhou, C. & Huang, A. Synthesis of highly stable graphene oxide membranes on polydopamine functionalized supports for seawater desalination. *Chemical Engineering Science* **146**, 159-165, doi:10.1016/j.ces.2016.03.003 (2016).

81 Qian, Y., Zhou, C. & Huang, A. Cross-linking modification with diamine monomers to enhance desalination performance of graphene oxide membranes. *Carbon* **136**, 28-37, doi:10.1016/j.carbon.2018.04.062 (2018).

82 Huang, A. & Feng, B. Synthesis of novel graphene oxide-polyimide hollow fiber membranes for seawater desalination. *Journal of Membrane Science* **548**, 59-65, doi:10.1016/j.memsci.2017.11.016 (2018).

83 Zhang, R., Liang, B., Qu, T., Cao, B. & Li, P. High-performance sulfosuccinic acid cross-linked PVA composite pervaporation membrane for desalination. *Environ Technol*, 1-9, doi:10.1080/09593330.2017.1388852 (2017).
